# Characterisation of *Candida* within the Mycobiome/Microbiome of the Lower Respiratory Tract of ICU Patients

**DOI:** 10.1371/journal.pone.0155033

**Published:** 2016-05-20

**Authors:** Robert Krause, Bettina Halwachs, Gerhard G. Thallinger, Ingeborg Klymiuk, Gregor Gorkiewicz, Martin Hoenigl, Jürgen Prattes, Thomas Valentin, Katharina Heidrich, Walter Buzina, Helmut J. F. Salzer, Jasmin Rabensteiner, Florian Prüller, Reinhard B. Raggam, Andreas Meinitzer, Christine Moissl-Eichinger, Christoph Högenauer, Franz Quehenberger, Karl Kashofer, Ines Zollner-Schwetz

**Affiliations:** 1 Section of Infectious Diseases and Tropical Medicine, Department of Internal Medicine, Medical University of Graz, Graz, Austria; 2 Bioinformatics, Institute for Knowledge Discovery, University of Technology, Graz, Austria and OMICS Center Graz, Graz, Austria; 3 BioTechMed-Graz, Graz, Austria; 4 Institute of Pathology, Medical University of Graz, Graz, Austria; 5 Institute of Molecular Biotechnology, University of Technology, Graz, Austria; 6 Center for Medical Research, Medical University of Graz, Graz, Austria; 7 Institute of Hygiene, Microbiology and Environmental Medicine, Medical University of Graz, Graz, Austria; 8 Department of Medicine I, University Hospital Carl Gustav Carus, Technische Universität Dresden, Dresden, Germany; 9 Division of Clinical Infectious Diseases, Research Center Borstel, Leibnitz Center for Medicine and Biosciences, Borstel, Germany; 10 Clinical Institute of Medical and Chemical Laboratory Diagnostics, Medical University of Graz, Graz, Austria; 11 Division of Angiology, Department of Internal Medicine, Medical University of Graz, Graz, Austria; 12 Theodor Escherich Laboratory for Microbiome Research, Division of Gastroenterology and Hepatology, Department of Internal Medicine, Medical University of Graz, Graz, Austria; 13 Institute for Medical Informatics, Statistics, and Documentation, Medical University of Graz, Graz, Austria; Leibniz Institute for Natural Products Research and Infection Biology- Hans Knoell Institute, GERMANY

## Abstract

Whether the presence of *Candida spp*. in lower respiratory tract (LRT) secretions is a marker of underlying disease, intensive care unit (ICU) treatment and antibiotic therapy or contributes to poor clinical outcome is unclear. We investigated healthy controls, patients with proposed risk factors for *Candida* growth in LRT (antibiotic therapy, ICU treatment with and without antibiotic therapy), ICU patients with pneumonia and antibiotic therapy and candidemic patients (for comparison of truly invasive and colonizing *Candida spp*.). Fungal patterns were determined by conventional culture based microbiology combined with molecular approaches (next generation sequencing, multilocus sequence typing) for description of fungal and concommitant bacterial microbiota in LRT, and host and fungal biomarkes were investigated. Admission to and treatment on ICUs shifted LRT fungal microbiota to *Candida spp*. dominated fungal profiles but antibiotic therapy did not. Compared to controls, *Candida* was part of fungal microbiota in LRT of ICU patients without pneumonia with and without antibiotic therapy (63% and 50% of total fungal genera) and of ICU patients with pneumonia with antibiotic therapy (73%) (p<0.05). No case of invasive candidiasis originating from *Candida* in the LRT was detected. There was no common bacterial microbiota profile associated or dissociated with *Candida spp*. in LRT. Colonizing and invasive *Candida* strains (from candidemic patients) did not match to certain clades withdrawing the presence of a particular pathogenic and invasive clade. The presence of *Candida spp*. in the LRT rather reflected rapidly occurring LRT dysbiosis driven by ICU related factors than was associated with invasive candidiasis.

## Introduction

The role of *Candida species* in the lower respiratory tract (LRT) has been under discussion for more than half a century [1]. In critically ill intubated and mechanically ventilated patients *Candida spp*. are frequently isolated from LRT secretions such as endotracheal aspirates or bronchoalveolar lavages (BALs) with uncertain significance. In autopsy studies invasive pulmonary *Candida* infection was considered to be very rare [2] or even absent [3,4]. On the other hand, recent studies demonstrated that the presence of *Candida spp*. in the LRT was associated with worse clinical outcome [5,6]. It is unclear from these studies whether *Candida spp*. colonization of LRT secretions is a marker of underlying disease severity, intensive care unit (ICU) treatment and antibiotic therapy or in fact contributes to poor clinical outcomes.

As shown previously, culture-based approaches are inadequate to completely understand the interactions of the host and the microbiome, and culture independent molecular assays are more efficient in describing microbial communities [7]. Using such techniques, differences between ICU patients with pneumonia compared to ICU patients without pneumonia (considered as controls) have been described [8]. However, the clinical significance of detected microorganisms and their role in the etiology of pneumonia could not be elucidated as findings were not compared to subjects with healthy respiratory tract and to subjects with certain risk factors considered to influence fungal and bacterial microbiota [7–9]. Beside other risk factors, antibiotic therapy and treatment on ICUs have been described as risk factors for *Candida* colonization and invasive candidiasis but the influence of antibiotic therapy on *Candida* within the LRT has not been investigated [10]. Recently, biomarkers were used to assess *Candida* pathogenicity, including (1–3)-ß-D Glucan test with high negative predictive value for invasive candidiasis or IL-17A and kynurenine levels showing high sensitivity for invasive candidiasis illustrated by ROC analysis [11,12].

To examine the presence of *Candida* in the LRT embedded within fungal and bacterial microbiota we investigated healthy controls, patients with proposed risk factors for *Candida* growth in LRT (antibiotic therapy, ICU treatment with and without antibiotic therapy), and intubated and mechanically ventilated ICU patients with pneumonia and antibiotic therapy. Furthermore, for comparison of truly invasive *Candida spp*. and colonizing *Candida spp*. candidemic patients were also included. Results of fungal patterns including data from conventional microbiology combined with molecular approaches (next generation sequencing, multilocus sequence typing) for description of fungal and concommitant bacterial microbiota as well as host (kynurenine) and fungal biomarkes ((1–3)-ß-D Glucan) are reported.

## Material and Methods

### 1. Patients

The study protocol of this observational study was approved by the local ethics committee, Medical University of Graz (approval number 19–322 ex 07/08). Adult patients >18 years of age were prospectively recruited at the Medical University of Graz and, after giving written informed consent, were assigned to one of groups 1–4 according to prospectively established inclusion and exclusion criteria as listed below. Unconscious intubated and mechanically ventilated patients were assigned to patient groups 2 or 3b according to the inclusion and exclusion criteria, had study related tests and were asked for study participation after their arousal according to approval of the ethical committee. Demographic, clinical and laboratory data were extracted from charts and computerized databases. Assessment of simplified acute physiology score (SAPS) changed from SAPS 2 to SAPS 3 during the study period due to modifications in the routinely used ICU clinical database. The study was registered at clinicaltrials.gov (clinicaltrials.gov identifier: NCT00786903).

Group 1a consisted of healthy adult patients with healthy respiratory tract (i.e. no known respiratory tract disease or complaints or x-ray indicative of respiratory tract diseases) undergoing elective plastic surgery and not receiving antibiotic therapy other than optional single shot surgical prophylaxis as clinically indicated. Surgical prophylaxis consisted of single administration of an antibiotic according to local guidelines 30–60 minutes prior to skin incision. Exclusion criteria were clinical or radiological or laboratory evidence of current infectious disease (temperature >38°C, elevated CRP >5mg/dl, leukocytosis >11400/μl, elevated neutrophiles >78%); antifungal therapy within 8 weeks prior to inclusion; history of pulmonary disease (e.g. COPD, asthma bronchiale, sarcoidosis, interstitial lung disease, malignant diseases of the lung); immunosuppressive therapy (e.g. glucocorticoids, methotrexate, azathioprin, etc); malignant hematological or oncological diseases; HIV positivity. To gain access to the lower respiratory tract without invasive procedures solely for study purpose the local ethics committee allowed to include these healthy patients while undergoing general anaesthesia and intubation due to elective surgical procedures (e.g. plastic surgery). This group served as the control group and was used for comparative data analysis (e.g. influence of antibiotic therapy on indigenous bacterial and fungal flora/microbiota within the LRT; comparison of *Candida spp*. colonization between healthy individuals and critical ill patients described below).

Group 1b consisted of adults with healthy respiratory tract (i.e. no known respiratory tract disease or complaints or x-ray indicative of respiratory tract diseases) undergoing elective surgery and receiving antibacterial therapy ≥2 days for treatment of extrapulmonary infectious diseases. Exclusion criteria for group 1b were antifungal therapy within 8 weeks prior to inclusion; history of pulmonary disease (e.g. COPD, asthma bronchiale, sarcoidosis, interstitial lung disease, malignant diseases of the lung); immunosuppressive therapy (e.g. glucocorticoids, methotrexate, azathioprin, etc); malignant hematological or oncological diseases; HIV positivity. Samples from LRT were obtained similar to group 1a. This group was used to assess the influence of antibiotic therapy on indigenous bacterial and fungal flora/microbiota of the LRT.

Group 2a consisted of non-neutropenic intubated and mechanically ventilated patients from the medical ICU without community-acquired (CAP), aspiration (ASP), nosocomial (NAP) or ventilator-associated pneumonia (VAP) according to previous published case definitions and without antibiotic therapy [13–15]. Exclusion criteria were history of pulmonary disease (e.g. COPD, asthma bronchiale, sarcoidosis, interstitial lung disease, malignant diseases of the lung), antifungal therapy within 8 weeks prior to inclusion; immunosuppressive therapy (e.g. glucocorticoids, methotrexate, azathioprin, etc); malignant hematological or oncological diseases; HIV positivity.

Group 2b patients consisted of non-neutropenic intubated and mechanically ventilated patients from the medical ICU without CAP, ASP, NAP or VAP according to previous published case definitions but with antibiotic therapy for ≥2 days for treatment of extrapulmonary infections [13–15]. Exclusion criteria were history of pulmonary disease (e.g. COPD, asthma bronchiale, sarcoidosis, interstitial lung disease, malignant diseases of the lung), antifungal therapy within 8 weeks prior to inclusion; immunosuppressive therapy (e.g. glucocorticoids, methotrexate, azathioprin, etc); malignant hematological or oncological diseases; HIV positivity.

Group 2a and 2b were used to assess the influence of ICU related factors on bacterial and fungal flora/microbiota within the LRT and served as comparator groups for comparison with intubated and mechanically ventilated patients with pneumonia.

Group 3b consisted of non-neutropenic intubated and mechanically ventilated ICU patients with CAP, ASP, NAP or VAP according to recently published case definitions and antibiotic therapy [13–15]. X-rays of the lungs were examined by blinded investigators. Exclusion criteria were antifungal therapy within 8 weeks prior to inclusion; immunosuppressive therapy (e.g. glucocorticoids, methotrexate, azathioprin, etc); malignant hematological or oncological diseases; HIV positivity. There was no group 3a (“b”refers to antibiotic therapy according to the other groups).

Group 4 consisted of patients with documented candidemia (i.e. clinical signs of systemic inflammatory response syndrome with *Candida spp*. in blood cultures). Exclusion criteria were immunosuppressive therapy (e.g. glucocorticoids, methotrexate, azathioprin, etc); neutropenia, HIV positivity. This group constituted the proven invasive *Candida* infection group. Data from this group were used for comparison reasons.

### 2. Sampling

#### 2.1. Group 1a and 1b

At the end of the elective surgical procedure and during termination of general anaesthesia endobronchial secretion (EBS) was obtained through the endobronchial tube (to avoid contamination of samples from oral or pharyngeal bacterial and fungal microbiota during sampling) by the anaesthesiologist and the study physician, captured in a sterile cup and immediately brought to the inhouse microbiology laboratory. An oral swab was obtained prior to sampling EBS. In accordance to recent literature showing similar community compositions by 16s rRNA sequencing between EBS and BAL from single individuals and according to the local ethical approval (not allowing lavage in group 1) BAL was not performed in group 1 patients [16].

#### 2.2. Group 2a and 2b

Samples from the LRT were obtained by bronchoscopy through endotracheal tubes (to avoid contamination of samples from oral or pharyngeal bacterial and fungal microbiota during sampling) and BAL (20 ml normal saline) of the right lung. Oral swabs were obtained prior to bronchoscopy.

#### 2.3. Group 3b

Samples from the LRT were obtained within 24 hours of clinically (including x-ray) established diagnosis of CAP, ASP, NAP or VAP by bronchoscopy (to avoid contamination of samples from oral or pharyngeal bacterial and fungal microbiota during sampling) and BAL (20 ml normal saline) through endotracheal tubes and directed to pulmonary infitrates suggestive of CAP, ASP, NAP or VAP. In case of infiltrates in both lungs samples from both sites were obtained and processed separately. BAL was captured in a sterile cup and used for routine and study purpose. An oral swab was performed prior to bronchoscopy. A second bronchoscopy and BAL was performed 4–7 days after the first sampling in patients still intubated and mechanically ventilated. Samples for calculation of *Candida* colonization index were obtained concomitantly to bronchoscopy.

#### 2.4. Group 4

Routine blood cultures obtained from patients in our Department of Internal Medicine and routinely processed in our inhouse laboratory were observed for *Candida* positivity. *Candida spp*. were subsequently identified by routine microbiological methods including MALDI-TOF-mass-spectrometry, stored at -70°C and processed as described below.

Respiratory samples from all patients were immediately brought to the inhouse microbiology laboratory, aliquoted, processed and remaining samples stored at -70°C until further analysis (e.g. microbiome analysis). Serum samples were obtained from all patients simultaneously with microbiological sampling, were centrifugated and stored at -70°C until further analysis. Three pairs of blood cultures were obtained concomitantly to blood samples mentioned above in patients with clinical and laboratory signs of systemic inflammatory response syndrome (i.e. tachycardia, leucocytosis, tachypnea, and temperature >38°C or <36°C), those were all patients from groups 1b, 2b, 3b and 4. *Candida* cultures for calculation of the *Candida* colonization index (CI) were performed in patient groups 2 and 3b. Swabs from wounds, catheter insertion sites, perineal region, oropharynx, as well as BAL and urine were cultured for calculation of CI as described previously [10].

#### 2.5. Comparison of sampling techniques (EBS vs. BAL)

Sampling techniques (collection of endobronchial secretion, EBS, versus BAL) and sterile saline used for BAL were compared in additional five intubated and mechanically ventilated ICU patients. Prior to routinely scheduled bronchoscopy (and BAL) endobronchial secretion was sampled and collected in a sterile cup. The bronchoscope was then flushed with 10ml of a given bottle of sterile saline used for subsequent BAL and the fluid was collected in a sterile cup. Then BAL was obtained by bronchoscopy and the sample collected in a sterile cup. Consequently, we obtained three sterile cups with fluid samples for bacterial and fungal microbiota comparison of EBS, BAL and sterile saline used for BAL corresponding to one patient. BAL was additionally processed and cultured in the routine laboratory if necessary for routine purpose.

### 3. Processing of fluid samples, swabs and blood cultures

#### 3.1. Quantitative *Candida* cultures and bacterial cultures

Quantitative *Candida* cultures and bacterial cultures from respiratory samples were performed by routine microbiological procedures. Briefly, samples from LRT were vortexed and 100 μL transferred onto a *Candida* CHROMagar (Becton Dickinson, Heidelberg, Germany), chocolate agar, blood agar, McConkey agar and was plated evenly with a sterile swab. After incubation at 37°C for 48 h in ambient air, the colonies were counted and classified as *C*. *albicans*, *C*. *glabrata*, *C*. *krusei*, *C*. *tropicalis*, or other *Candida spp*., according to the color of the colonies and were further identified by MALDI-TOF-mass-spectrometry. Quantitative bacterial cultures were performed by routine microbiological procedures.

#### 3.2. Blood cultures

Three pairs of blood culture bottles were drawn as recommended by local blood culture instructions and incubated at 37°C in an automatic blood culture detection system (BACTEC^®^ 9240 or BACTEC FX^®^, Becton Dickinson, Heidelberg, Germany) for 5 days that allows continuous monitoring of blood cultures for microbial growth. Broth from positive blood culture bottles was Gram-stained and subcultured on specific agars depending on the Gram stain results. Fungi and/or bacteria were identified by routine microbiological procedures including API (bioMérieux, Marcy-l’Etoile, France), and peptid nucleic acid (PNA) FISH tests allowing quick identification of certain microorganisms within 20–90 minutes. *Candida* isolates were also identified by MALDI-TOF-mass-spectrometry. Isolated strains were stored at a temperature of -70°C until further analysis (e.g. susceptibility testing, multilocus sequence typing).

#### 3.3. Urine and swab cultures

Cultures from urine were performed by routine microbiological procedures. *Candida* isolates were counted and identified by MALDI-TOF-mass-spectrometry. Cultures from swabs were performed on *Candida* CHROMagar (Becton Dickinson, Heidelberg, Germany) at 37°C for 48 h in ambient air and *Candida* isolates were counted and identified as described above.

#### 3.4. *Candida* colonization index

In patient groups 2 and 3b swabs from wounds, catheter insertion sites, perineal region, oropharynx, as well as BAL and urine were cultured for calculation of *Candida* colonization index as described previously [10].

### 4. Antifungal susceptibility testing of *Candida* species

*Candida* strains were tested against amphotericin B, flucytosin, fluconazole, voriconazole, posaconazole, anidulafungin, caspofungin and micafungin using E-test strips according to the manufacturer’s instructions (bioMérieux, Marcy-l’Etoile, France) and EUCAST guidelines [17]. Putative mutations in hot spots of the FKS gene complex were determined as described previously [18].

### 5. Multilocus sequence typing (MLST)

To determine relatedness of colonizing and invading *Candida albicans* strains randomly selected twenty of 25 oral *Candida albicans* isolates from group 1 as well as randomly selected 22 of 26 blood culture *Candida albicans* isolates from group 4 were investigated by MLST. Two of candidemic patients had oral *Candida albicans* isolates which were also included in the analysis. Three isolates did not provide adequate PCR results and were excluded from analysis (1 oral colonizing and 2 invasive isolates).

#### 5.1. DNA extraction for MLST

DNA extraction was carried out by using the PrepMan^®^ Ultra Sample Preparation Reagent (Applied Biosystems, Foster City, CA). 200 μL of PrepMan Buffer and 1 loop from a pure culture of each sample were mixed thoroughly in 1.5-mL tubes and heated in boiling water for 12 min. After a 3 min centrifugation step at 16,100g, the DNA-containing supernatant was decanted and transferred into a new tube.

#### 5.2. Multilocus sequence typing (MLST)

5.2.1. Primer design and PCR: Seven primer pairs specific for DNA sites encoding housekeeping genes were chosen as recommended previously [19–21]: AAT1a, (Aspartateaminotrasferase), fragment size 373, chromosome 2, Fwd 5’-ACTCAAGCTAGATTTTTGGC- 3’, Rev 5’-CAGCAACATGATTAGCCC- 3’; AAC1 (Acetyl-coenzyme A carboxylase), fragment size 407, chromose R, Fwd 5’-GCAAGAGAAATTTTAATTCAATG- 3’, Rev 5’-TTCATCAACATCATCCAAGTG- 3’; ADP1 (ATP-dependent permease), fragment size 443, chromose 1, Fwd 5’-GAGCCAAGTATGAATGATTTG- 3’, Rev 5’-TTGATCAACAAACCCGATAAT- 3’, PMI1b^a^, (formerly MPI1b) (Mannose phosphate isomerase), fragment size 375, chromosome 2, Fwd 5’-ACCAGAAATGGCCATTGC- 3’, Rev 5’-GCAGCCATGCATTCAATTAT- 3’; SYA1 (Alanyl-RNA synthetase), fragment size 391, chromosome 6, Fwd 5’-AGAAGAATTGTTGCTGTTACTG- 3’, Rev 5’-GTTACCTTTACCACCAGCTTT- 3’, VPS13 (Vacuolar protein sorting protein), fragment size 403, chromosome 4, Fwd 5’-TCGTTGAGAGATATTCGACTT- 3’, Rev 5’-ACGGATGGATCTCCAGTCC- 3’; ZWF1b, (Glucose-6-phosphate dehydrogenase), fragment size 491, chromosome 1, Fwd 5’-GTTTCATTTGATCCTGAAGC- 3’, Rev 5’-GCCATTGATAAGTACCTGGAT- 3’. According to previous literature these genes enable high discriminatory power in *C*. *albicans* MLST investigations [20,21]. Primers were commercially produced by Invitrogen^™^ Custom DNA Oligos (life technologies, Carlsbad, CA). The primer stock solution was diluted with PCR-grade water to obtain a concentration of 10 μM/L. PCR was carried out with total reaction volumes of 50 μL, containing 37 μL water, 5 μL 10x Taq Buffer, 1 μL dNTP Mix, 1 μL Taq DNA Polymerase (5prime, Hilden, Germany), 2 μL of the forward and reverse primer (10 μM) and 2 μL DNA template. PCRs were performed on an Applied Biotechnologies 2720 Thermal Cycler (life technologies, Carlsbad, CA). The duration and temperature of the cycles were chosen as previously described [22].

5.2.2. Purification: The PCR products were purified using the MSB^®^ Spin PCRapace purification system (STRATEC molecular, Berlin, Germany). Each PCR sample was mixed with 250 μL binding buffer and transferred onto the enclosed spin filter. After 3 min centrifugation at 13,400 g, the filter was removed and transferred into a new tube. 50 μL elution buffer were added prior to a further centrifugation step for 1 min at 9,200g. The filter was then discarded, the supernatant contained the purified PCR product.

5.2.3. Cycle sequencing: Cycle sequencing was arranged using the BigDye^®^ Terminator v3.1 Cycle Sequencing Kit (life technologies). The primer stock solution was diluted with PCR-grade water to obtain a concentration of 1.6 μMol/L. Some amplicons, most notably SYA1 products, had low chromatogram signals or a high noise signal. In this case, agarose gel electrophoresis was performed. Whether gel electrophoresis revealed narrow bands, the amount of template was raised. Cycle sequencing was carried out on an Applied Biosystems 2720 Thermal Cycler. An initial step of 94°C for 18 sec was followed by 25 cycles of 94°C for 15 sec, 50°C for 10 sec and 60°C for 2 min with a final extension step at 60°C for 4 min. Further purification steps followed the cycle sequencing. The cycle sequencing products were vortexed in a 1.5-mL tube with 25 μL 98% ethanol (v/v) and 2.5 μL 125 μM EDTA and then centrifuged for 20 minutes at a temperature of 4°C at 16,100 g. After discarding the supernatant, 200 μL of 70% ethanol (v/v) were added and another centrifugation step for 15 min at a temperature of 4°C at 16,100g was done. Thereafter the supernatant was removed and the pellet was dried in an incubator at 37°C for at least 1 h. Then the pellet was resuspended in 15 μL Applied Biosystems^®^ Hi-DiTM Formamide (life technologies) and pipetted onto a 96-well plate. Analysis was performed on the Applied Biosystems^®^ 3130 Genetic Analyzer (life technologies).

5.2.4. MLST Data analysis: Analysis of the raw sequencing data succeeded with Applied Biosystems DNA Sequencing Analysis Software (life technologies, Carsbad, CA). After analysis and post-processing, each forward and reverse sequence pair was assembled with the help of Lasergene^®^ SeqMan Pro software (DNAStar, Madison, WI). Any assembly was searched automatically and manually for single nucleotide polymorphisms (SNPs). The SNPs were badged using the IUPAC nucleotide code according to previous literature [23]. Each consensus sequence was compared with the database on the MLST website to obtain the allele number (ST—sequence type) and the MLST profile number (DST—diploid sequence type). New alleles and DSTs were sent to the curator of the MLST website to obtain the allelic and DST numbers. The 7 matching sequences of each isolate were concatenated and afterwards aligned by means of muscle (http://drive5.com/muscle/) according to previous literature [24,25]. Our strains were added to the *C*. *albicans* MLST database (calbicans.mlst.net) and they are available using the following headings: id = 2275–2315 or Sequencer = Buzina Walter. In order to assign the strains to their clades, an eBURST analysis was performed acording to previous literature [26].

5.2.5. Phylogenetic analysis of MLST data: Evolutionary analyses, using neighbor-joining and Unweighted Pair Group Method with Arithmetic mean (UPGMA method), were conducted in MEGA6 (www.megasoftware.net) [27].

### 6. Next generation sequencing (NGS)

#### 6.1. DNA isolation and PCR amplification

Fifty-four randomly selected LRT samples were submitted to NGS-based fungal and bacterial microbiota analyses comprising 8 specimens from group 1a, 7 from group 1b, 7 from 2a, 6 from 2b and 26 from group 3b. For 10 samples, the ITS PCR did not produce any amplicons and samples were therefore excluded from analyses for fungal microbiota (4 in group 1a, 2 in group 1b, 4 in group 3b). In one sample the 16S rRNA gene PCR did not produce any amplicons and this sample was therefore excluded from analysis of bacterial diversity. For total DNA isolation from 5 mL BAL/EBS samples, Dithiothreitol (DTT) at a final concentration of 0.15% (w/v) was added, samples were incubated until dissolved and centrifuged at 4,000g for 30 min. Cells pellets were homogenized in 250 μL PBS and mixed with 250 μL bacteria lysis buffer in MagnaLyser bead tubes by using the MagnaLyser Instrument (Roche Diagnostics, Mannheim, Germany) according to manufacturer’s instructions. DNA was extracted using the MagNA Pure LC DNA Isolation Kit III (Bacteria, Fungi) with 25 μL lysozyme (100 mg/mL) (Roche Diagnostics, Mannheim, Germany), 20 μL Proteinase K (20 mg/mL) and three consecutive freeze-thaw cycles in a MagNA Pure LC 2.0 Instrument according to manufacturer’s instructions. DNA was quantified by quantitative real time PCR as described previously [28]. 16S rRNA gene hypervariable regions V1-2 (F27-5’-AGAGTTTGATCCTGGCTCAG-3’, R357-5’CTGCTGCCTYCCGTA-3’) [29,30] were amplified from total DNA by PCR using FLX 454 one way read (Lib-L kit, Primer A, Primer B, Roche 454 Life Science, Branford, CT, USA) fusion primers bearing the titanium FLX adaptor (5’-CCATCTCATCCCTGCGTGTCTCCGAC-3’), a key sequence (5’-TCAG-3’), a 10-mer Multiplex Identifier (MID) and the target specific forward sequence (5’-AGAGTTTGATCCTGGCTCAG-3’). The reverse fusion primer sequence was 5’-CCTATCCCCTGTGTGCCTTGGCAGTC-TCAG-CTGCTGCCTYCCGTA-3’. For each sample a mix of 25 μL containing 1 x Fast Start High Fidelity Buffer (Roche Diagnostics, Mannheim, Germany), 2.5 U High Fidelity Enzyme (Roche Diagnostics, Mannheim, Germany), 200μM dNTPs (Roche Diagnostics, Mannheim, Germany), 0.4μM barcoded primers (Eurofins MWG, Ebersberg, Germany), PCR-grade water (Roche Diagnostics, Mannheim, Germany) and 6.5ng genomic DNA. PCR was performed in triplicates. Thermal cycling was of initial denaturation at 95°C for 3 min followed by 35 cycles of denaturation at 95°C for 45 sec, annealing at 56°C for 45 sec and extension at 72°C for 1 min with a final extension of 7 min at 72°C. Amplicons were purified according to standard procedures. Amplicon DNA quantification, pooling and sequencing was performed as recently described [31]. For internal transcribed spacer (ITS) amplification FLX Titanium Fusion primers with the target specific forward ITS1F 5’-CTTGGTCATTTAGAGGAAGTAA-3’ and reverse ITS2R 5’-GCTGCGTTCTTCATCGATGC-3’ sequences were used [32]. For ITS PCR the same PCR mix was run with 50ng total genomic DNA and the cycling conditions as previously described [32]. Subsequent procedure was performed according to the 16S rRNA gene protocol.

#### 6.2. Phylogenetic analysis

To improve overall analysis quality as well as to avoid OTU inflation due to sequencing errors data was de-noised using previously published methods [33]. Additionally, contaminant sequences (such as sequences originating from the human host, or from other non 16S or ITS sources) were detected and removed by the Decontaminator with 80% identity and 15% query coverage. Briefly, amplicons were aligned using BLAT [34] against the greengenes 16S version (09.05.2011) and the UNITE ITS version (15.10.2013) database [35]. respectively. BLAT hits were sorted descending according their percentage of identity and quality coverage. Amplicons which fall below the specified criteria or with no hit at all were identified as contaminating sequence and removed. Furthermore, all sequences shorter than 150 bps containing any ambiguous characters, or not matching to the forward primer (distance>2) or the multiplexing barcodes were discarded. Subsequently, chimeric sequences were identified for both datasets with UCHIME [36] and removed. The remaining sequences after the pre-processing process were assigned to their respective samples by using the 10 bp barcode preceding the primer. In order to perform sample- and group-wide comparisons, operational taxonomic units (OTUs) were generated with two distinct phylotyping pipelines of SnoWMAn (http://SnoWMAn.genome.tugraz.at) [37]. For the 16S dataset OTUs were generated with an extended Ribosomal Database Project (RDP)-Pyrosequencing approach [38], by using the integrated RDP pipeline of SnoWMAn. Briefly, all sequences were pooled and aligned with Infernal (V1.0) using a 16S rRNA secondary structure based model for accurate position alignment of sequences [39]. The aligned sequences were clustered by complete linkage to form OTUs with sequence distances ranging from 0% to 5%. For each OTU a representative sequence was extracted and a taxonomic classification was assigned to it using the RDP Bayesian classifier 2.5 [37]. Finally, the pooled sequences were again separated according to their sample affiliation. Taxonomic classification and biostatistical analyses reported in this paper were performed on the clustering results for 3% distance at a classification confidence threshold of 80%.

Phylogenetic classification of the ITS dataset was done using the advanced BLAT approach of SnoWMAn. Briefly, unique sequences were aligned with BLAT against the UNITE ITS reference database version (15.10.2013). Subsequently, the best BLAT hit was used to assign taxonomic classification to each amplicon. Finally, samples were de-uniqued again and samples which result in the same taxonomic classification were assigned to the same OTU.

#### 6.3. Statistical analysis and vizualization

The analyses were conducted using the statistical and visualization capabilities of SnoWMAn. Species richness [40] was estimated according to Chao1 [41]. Additionally, the abundance-based coverage estimator (ACE) [42], evenness [40], Shannon and Simpson diversity index were calculated. Furthermore, OTUs significantly changing between the different patient groups were assessed by the R Bioconductor package edgeR (version 3.2.4). Briefly, the feature matrices (i.e. OTU counts per sample) were imported into R. All counts were increased by 1 to prevent taking the log from 0 and stored within a DGEList object. To scale the raw library sizes, the calcNormFactors [43] function was applied using the relative log expression (RLE) method [44]. Subsequently, a model matrix according to the experimental design was created. The common dispersion of all biological coefficients of variation (BCV) averaged over all OTUs, as well as the OTU-specific dispersion of the dataset, were calculated (estimateGLMTagwiseDisp, estimateGLMTrendedDisp, respectively). Prior to the likelihood ratio test (glmLRT) [45,46] the read counts for each feature were fit to a negative binomial generalized log-linear model (glmFit). To account for multiple testing and control of the Type I error (FDR), p-values were adjusted using a Benjamini-Hochberg method [47]. Only features with an adjusted p-value less than 0.05 were considered as differentially abundant.

#### 6.4. Association and dissociation of bacterial (16S rRNA gene) and fungal (ITS) communities

Relationships between bacteria and fungi in the samples of the different patient groups were calculated as odds ratio as described previously [48]. Briefly, for all bacteria and fungi pairs a two by two contingency table was created, presenting their presence and absence in the samples. Based on this information the odds ratio for each pair was calculated using the odds ratio function of R (package vcd_1.3–1. R version 3.0.3). The odds ratios above 2 were considered a positive association as this means that the detection of a particular organism with the other organism is twice as likely as with its absence. In contrast, an odds ratio below 0.5 was interpreted as negative association.

#### 6.5. Sequence data availability

Sequence data generated for this work can be accessed via the European Bioinformatics Institute Sequence Read Archive (EBI SRA) under the accession number ERP008535.

### 7. Determination of serum (1–3)-ß-D Glucan Assay

Serum (1–3)-ß-D Glucan test was performed according to recently described methods [49].

### 8. Kynurenine and Tryptophane in LRT samples

Kynurenine and Tryptophane in LRT samples were determined in patient groups 1a, 2b, and 3b as described recently [12].

### 9. Statistical methods of *Candida* colonization, death rates, *Candida* colonization index, kynurenine and tryoptophan

Statistical analysis was performed using R 3.1.1 (www.r-project.org) and the package cherry version 0.5–10. Differences in *Candida* colonization and death rates (intra-hospital, 30-day) between groups in general and in all pairwise comparisons were tested by using the Fisher‘s exact test and corrected multiple pairwise comparisons using the closed testing procedure. Differences in *Candida* colonization index as well as in kynurenine levels, tryptophan levels and kynurenine/tryptophan ratio in LRT samples were tested by Kruskal-Wallis test. The Kruskal-Wallis test and Fisher’s exact test was used in Table 1. A p-value of <0.05 was considered statistically significant.

**Table 1:** Demographic data and clinical characteristics of study patients. ICU patients were all intubated and mechanically ventilated.

	1a, healthy adults (n = 87)	1b, non-ICU, extrapulmonary infection, AB therapy (n = 18)	2a, ICU, no AB therapy (n = 8)	2b, ICU, extrapulmonary infection, AB therapy (n = 23)	3b, ICU, pneumonia, AB therapy (n = 34)^α^	4, candidemia (n = 32)	p-value
age (IQR)	49 (40–57)	58 (49–68)	60 (50–67)	66 (54–78)	60 (50–70)	66 (58–73)	<0.001
male/female	32/55	8/10	4/4	18/5	28/6	20/12	0.03
underlying diseases	0	skin and soft tissue infection 61% (11/18)	CPR* 50% (4/8)	CPR* 52% (12/23)	CPR* 38% (13/34)	infectious disease^$^ 28% (9/32)	-
		cholecystitis with cholecystectomy 28% (5/18)	stroke 37% (3/8)	cardiovascular 22% (5/23)	stroke 9% (3/34)	metabolic disorder 6% (2/32)	-
		osteomyelitis 5.5% (1/18)	intoxication 13% (1/8)	stroke 13% (3/23)	infectious disease^§^ 23% (8/34)	gastrointestinal diseases 19% (6/32)	-
		Diverticulitis with abscess 5.5% (1/18)		infectious disease 13% (3/23)	electrolyte imbalance 3% (1/34)	others 47% (15/32)^&^	-
					gastrointestinal 15% (5/34)		-
					ARDS 9% (3/34)		-
					aspiration 3% (1/34)		-
ICU stay until inclusion/sampling (median; IQR); days	0	0	2 (1.8–2.5)	3 (1.5–5)	2 (1–4)	n.d.	0.15
mechanical ventilation until inclusion (median; IQR); days	0	0	2 (1–2.5)	3 (2–4.5)	2.5 (1–4)	n.d.	0.11
SAPS 2/3 (median, IQR)	n.d.	n.d.	SAPS 2 (5 patients) 62 (23–68)	SAPS 2 (14 patients) 26.5 (19.5–44.25)	SAPS 2 (19 patients) 30 (22.5–43.25)	n.d.	0.52
			SAPS 3 (3 patients) 53 (50–61)	SAPS 3 (9 patients) 60 (59–67)	SAPS 3 (15 patients) 63.5 (55.75–73.75)		0.57
Oxygenation index (IQR)	n.d.	n.d.	528 (355–665)	450 (266–556)	296 (168–579)	n.d.	0.31
Vasopressor/Catecholamine	0	0	5 (63%)	16 (70%)	26 /76%)	10 (31%)	0.0012
Proton pump inhibitor	0	18 (100%)	8 (100%)	22 (96%)	33 (97%)	23 (72%)	0.0034
Sedative(s)	0	0	3 (38%)	18 (78%)	29 (85%)	6 (19%)	<0.001
Morphine	0	0	4 (50%)	18 (78%)	30 (88%)	11 (34%)	<0.001
Enteral nutrition	0	0	6 (75%)	18 (78%)	24 (71%)	32 (100%)	0.0024
Parenteral nutrition	0	0	0 (0%)	1 (4%)	3 (9%)	3 (9%)^β^	0.78
Nasogastric tube	0	0	7 (88%)	23 (100%)	31 (91%)	11 (34%)	0.0024
Central venous catheter	0	0	7 (88%)	22 (96%)	33 (97%)	25 (78%)	0.062
Urinary catheter	0	18 (100%)	8 (100%)	23 (100%)	33 (97%)	26 (81%)	0.029
Antimycotic treatment after study procedures	0	0	0 (0%)	1 (4%)	6 (18%)	31 (97%)^#^	0.001
Intrahospital death	0	0	3 (38%)	9 (39%)	11 (32%)	10 (31%)	0.92
30-day mortality	0	0	3 (38%)	9 (39%)	11 (32%)	10 (31%)	0.92
*Candida* related death (related to total deaths)	0	0	0 (0%)	0 (0%)	0 (0%)	7 (70%)	0.074

IQR = interquartile range

^α^ = by conventional cultures the following pathogens were found in BALs: *E*. *coli* 10^2^CFU/ml (2 patients), *Staphylococcus aureus* 10^1^CFU/ml (1; this patients also had positive Influenza B PCR), *Enterobacter cloacae* 10^5^CFU/ml (1), *Pseudomonas aeruginosa* 10^2^CFU/ml (1),ß-haemolytic streptococci group A 10^3^CFU/ml (1), *Klebsiella sp*. 10^6^CFU/ml (1), *Proteus mirabilis* 10^1^CFU/ml (1), *Candida sp*. 1-10^5^CFU/ml (11), *Candida sp*. with *Staph*. *aureus*, *Serratia sp*., *E*. *coli* 10^6^CFU/ml (1), *Candida sp*. with *Staph*. *aureus* 10^1^CFU/ml (1), *Candida sp*. with *E*. *cloacae* 10^6^CFU/ml (1), 8 patients had negative cultures (1 of these patients had positive Influenza A H1N1 PCR); in the remaining 4 patients bacteria belonging to the oral cavity were cultured.

* = cardiopulmonary resuscitation (CPR) prior to study inclusion

^§^ = 5 patients with extrapulmonary infections prior to or concommitantly to pneumonia, 3 patients without any underlying disease except pneumonia

^$^ = underlying infectious diseases prior to candidemia

^&^ = postsurgical 5, malignant disease 4, bleeding and vascular disease 4, renal failure 1, unspecific clinical deterioration 1

^#^ = 1 patient did not receive antimycotic treatment due to palliative care

^β^ = 3 patients had both enteral and parenteral nutrition

n.d. = not determined

### 10. Ethics Statement

The study protocol was approved by the ethics committee, Medical University of Graz. All conscious patients provided written informed consent prior to study related procedures. Unconscious intubated and mechanically ventilated patients at ICU had study related tests and were asked for study participation after their arousal according to approval of the ethical committee. During preparation of study procedures and protocols the type of consent had been discussed with members of our ethical committee, i.e. to obtain consent from family members or to obtain retrospective consent from unconscious patients after they return to conscious condition and the procedure described above was suggested and approved by our local ethical committee. None of the patients declined to participate after having study related procedures. Samples from group 1 patients (without pneumonia) were taken for study purpose only. In ICU group 2 patients (without pneumonia) bronchoscopy and sampling of LRT was performed for study purpose only. In ICU group 3b patients (with pneumonia) and ICU patients included for sampling technique comparison bronchoscopy was performed as clinically indicated and samples were used for routine and study investigations. The second bronchoscopy in group 3b was routine in nature or solely for study investigations depending on the clinical course of the patients. Blood cultures were routine in nature since these cultures were routinely obtained in patients with SIRS and clinical suspicion of bloodstream infection (all patients from groups 1b, 2b, 3b and 4).

## Results

Two hundred-and-two patients were investigated consisting of 87 patients in group 1a (healthy adults), 18 in group 1b (patients with healthy respiratory tract but with antibiotic therapy for extrapulmonary infection), 8 in group 2a (non-neutropenic intubated and mechanically ventilated ICU patients without antibiotic therapy), 23 in group 2b (non-neutropenic intubated and mechanically ventilated ICU patients with antibiotic therapy for extrapulmonary infection), 34 in group 3b (non-neutropenic intubated and mechanically ventilated ICU patients with antibiotic therapy due to pneumonia), and 32 in group 4 (candidemic patients). Demographic data are depicted in Table 1. None of patients in group 1a and 1b died during the course of the study. There was no difference in intrahospital death and 30-day mortality rate between the ICU groups (38% in group 2a, 39% in group 2b, 32% in group 3b) and the candidemic group 4 (31%) (p = 1 for all comparisons). Two out of eight (25%), 9/23 (39%), and 10/34 (29%) patients in group 2a, 2b, and 3b had positive (1–3)-ß-D Glucan test results. Despite positive (1–3)-ß-D Glucan tests none of ICU patients suffered or deceased from invasive fungal infection as determined by clinical, imaging, laboratory and microbiological investigations (i.e. detection of *Candida spp*. in blood, sterile fluids or tissues).

**Fig 1:**
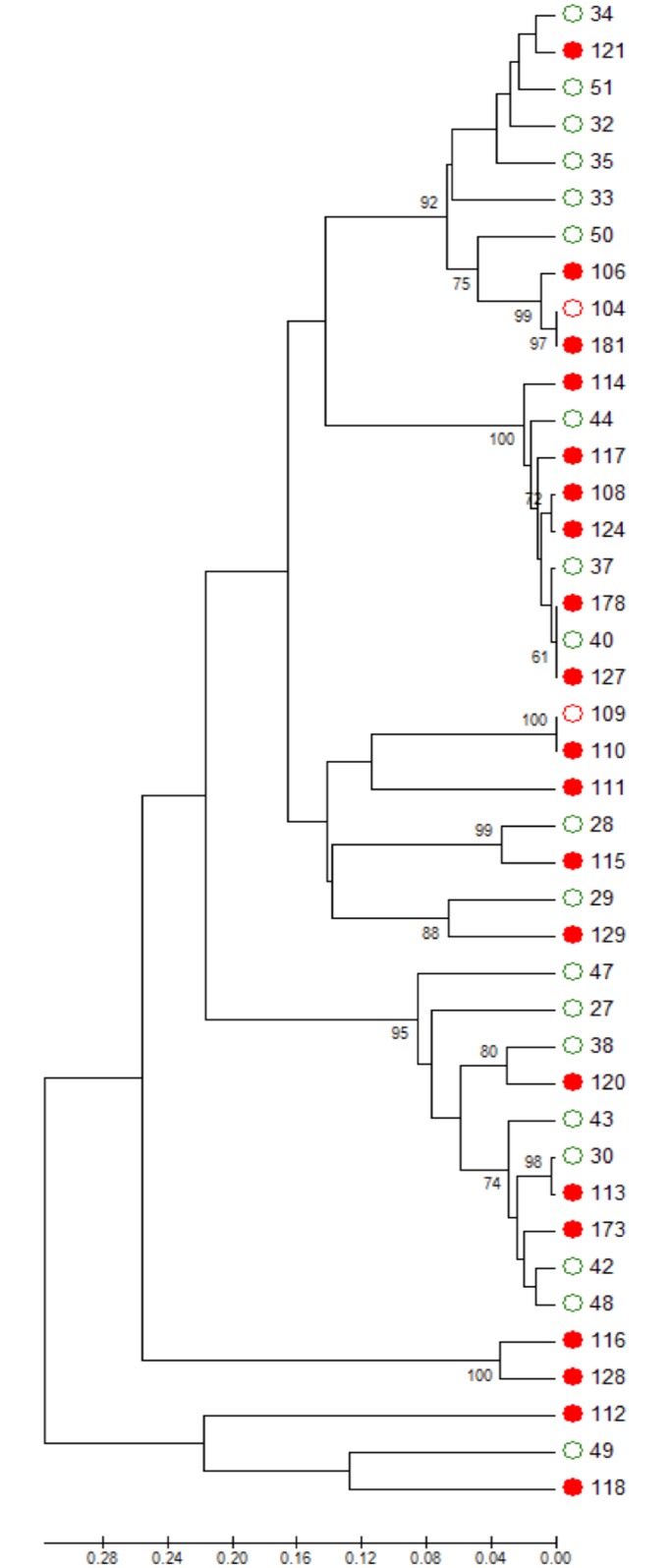
Neighbor-joining tree of invasive and colonizing *Candida* isolates. Red dots = invasive *Candida* isolates; green circles = colonizing *Candida* isolates from patients without candidemia; red circles = oral colonizing isolates from patients with candidemia. The percentage of replicate trees in which the associated taxa clustered together in the bootstrap test (1000 replicates) are shown next to the branches. The tree is drawn to scale, with branch lengths in the same units as those of the evolutionary distances used to infer the phylogenetic tree. The evolutionary distances are in the units of the number of amino acid differences per site.

Antibiotic therapy increased the prevalence of *Candida spp*. in the oral cavity and admission to ICU increased the prevalence of *Candida spp*. in the LRT as determined by conventional culture (p-values in Table 2). In total we cultured 139 *Candida albicans*, 27 *C*. *glabrata*, 4 *C*. *parapsilosis*, 4 *C*. *dubliniensis*, 2 *C*. *krusei*, 1 *C*. *tropicalis*, 1 *C*. *kefyr*, 1 *C*. *cerevisiae*, and 1 *C*. *boidinii* isolate(s) from LRT, urine, oral and perianal swabs and blood samples. Of those, 26 *C*. *albicans*, 2 *C*. *parapsilosis*, 1 *C*. *tropicalis*, 1 *C*. *glabrata* and 1 *C*. *krusei* isolate(s) were cultured from blood cultures from group 4 patients and comprised invasive *Candida* strains. *Candida* susceptibility testing revealed one Anidulafungin resistant *Candida albicans* isolate with homozygeous FKS S645F mutation. MLST was performed with randomly selected 44 isolates, comprising 17 orally colonizing isolates from group 1a and 22 invasive isolates obtained from blood cultures (group 4) as well as 2 oral samples from 2 candidemic patients. Three isolates did not provide adequate PCR results and were excluded from analysis (1 oral colonizing and 2 invasive isolates). There was no difference in distribution of invasive and colonizing isolates in different clades as determined by eBURST, neighbour joining or UPGMA analysis (Fig 1). Since there was no putative invasive *Candida* clade further MLST typing including *Candida spp*. from LRT samples was omitted.

**Table 2:** *Candida* positive cultures from the oral cavity, lower respiratory tract (LRT), and the *Candida* colonization index.

*Candida* cultures	1a, healthy adults, (n = 87)	1b, non-ICU, extrapulmonary infection, AB therapy (n = 18)	2a, ICU, no AB therapy (n = 8)	2b, ICU, extrapulmonary infection, AB therapy (n = 23)	3b, ICU, pneumonia, AB therapy (n = 34)
Oral cavity positive	25 (29%)*	14 (74%)*	4 (50%)	12 (52%)	20 (59%)*
LRT positive	1 (1%)**	2 (11%)	2 (25%)**	6 (26%)**	14 (41%)**
*Candida* colonization index (median)	n.d.	n.d.	0.25	0.25	0.25

* significant difference with p-values: group 1a vs. 1b < 0.001. 1a vs. 3b = 0.008

** significant difference with p-values: group 1a vs. 2a = 0.02. 1a vs. 2b < 0.001. 1a vs. 3b < 0.001

n.d. = not done

NGS analysis of EBS, BAL and saline used for BAL showed that saline did not influence microbiota results (as *Candida* was not present and fungal content reflecting other fungi was very low with 6% counts per OTU compared to BAL) and that endobronchial secretions and bronchoalveolar lavages yielded similar results (S1–S5 Figs).

**Fig 2:**
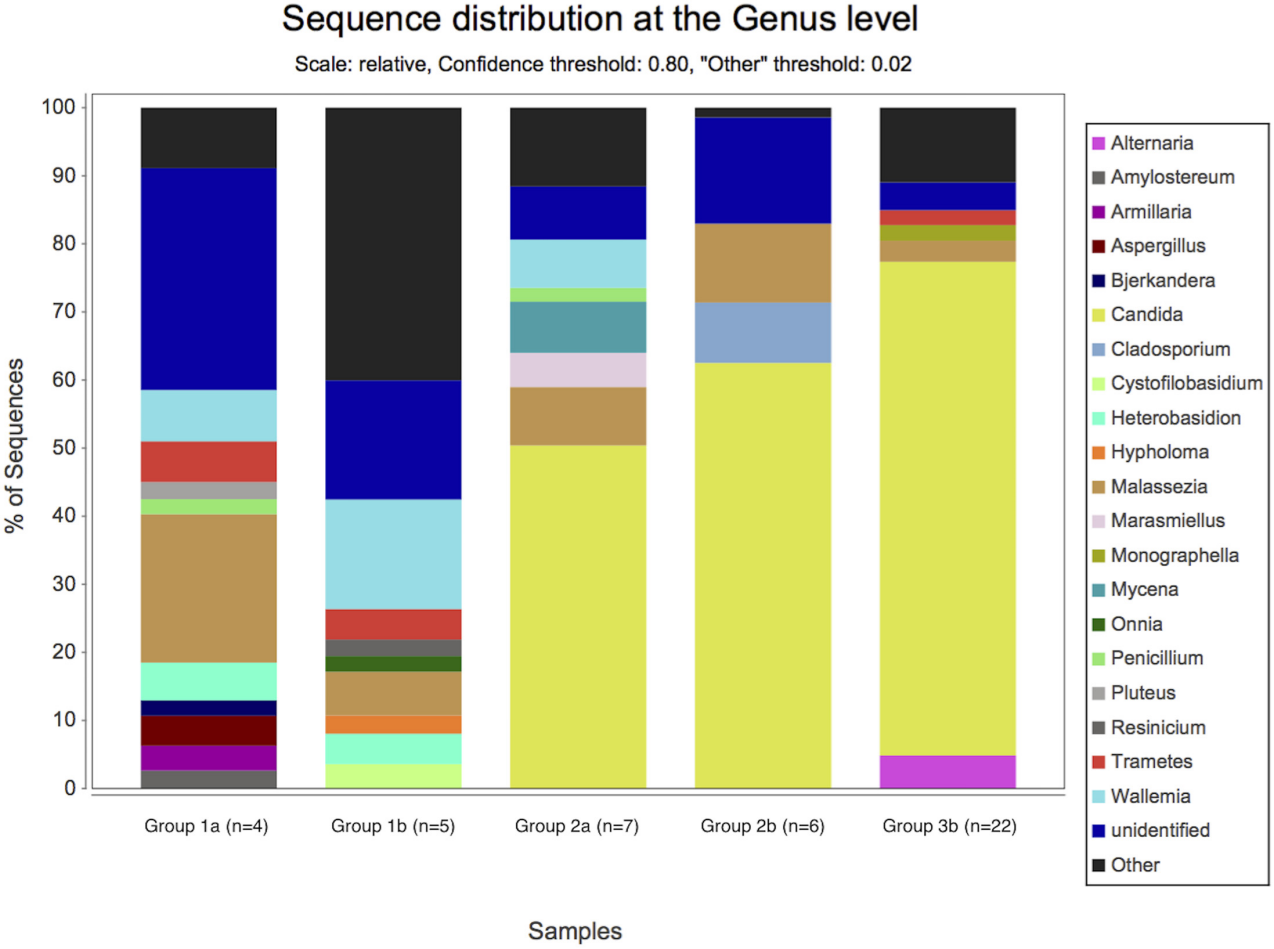
Relative abundance of fungal microbiota in lower respiratory tract samples. Healthy adults (bar group 1a), patients with healthy respiratory tract but with antibiotic therapy for extrapulmonary infection (bar group 1b), non-neutropenic intubated and mechanically ventilated ICU patients without antibiotic therapy (bar group 2a), non-neutropenic intubated and mechanically ventilated ICU patients with antibiotic therapy for extrapulmonary infection (bar group 2b), and patients with antibiotic therapy due to pneumonia (bar group 3b). Fungi are shown at genus level. Yellow bars represent the sum of *Candida* sequences across all samples per group related to the total number of detected fungal sequences within each of the groups. By conventional culture *Candida spp*. were detected in 0/8 (group 1a), 0/7 (group 1b), 1/7 (group 2a), 3/6 (group 2b), and 11/26 (group 3b) patients. No other fungi were cultured.

**Fig 3:**
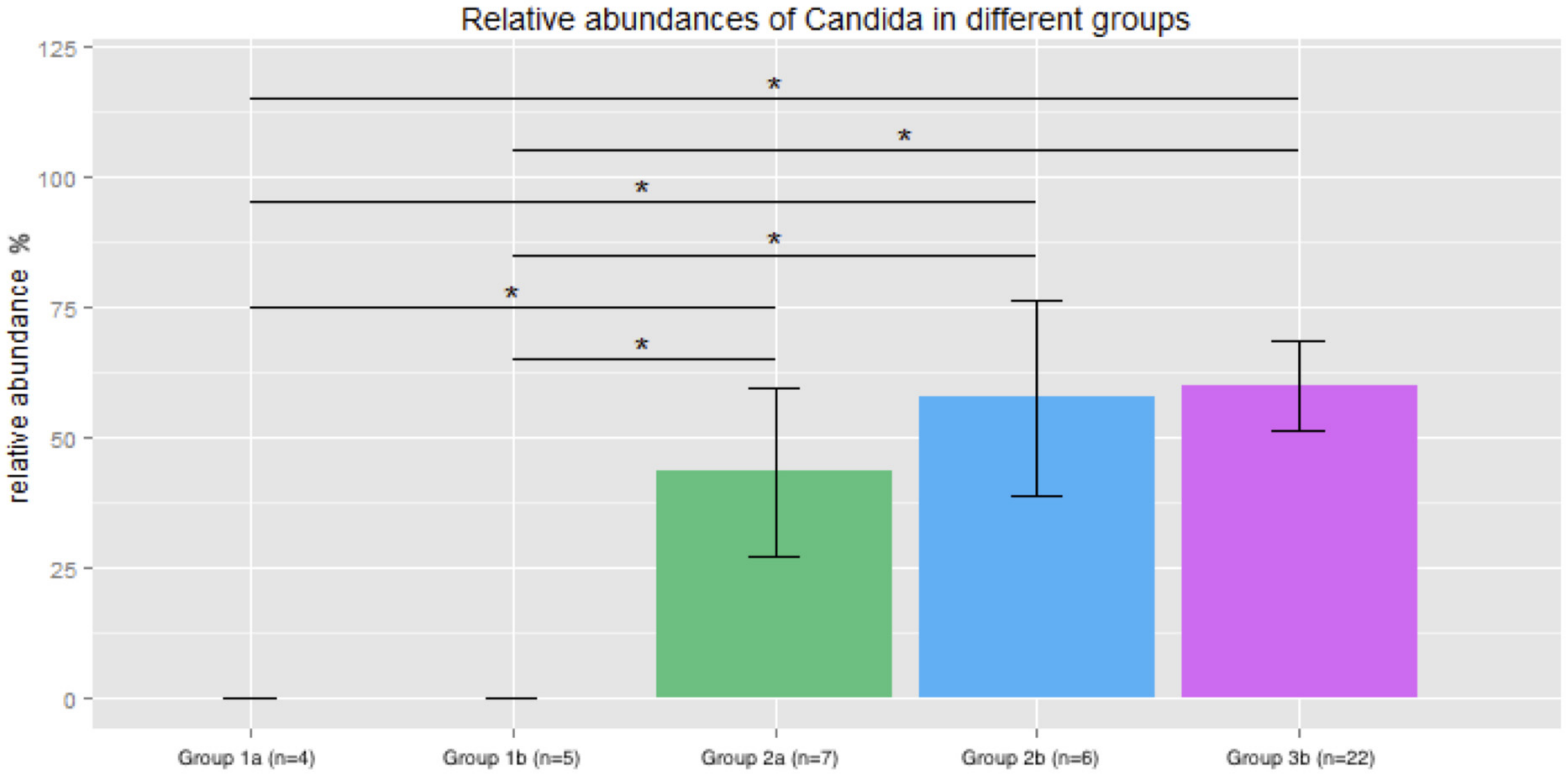
Average relative *Candida* abundance in lower respiratory tract samples. Healthy adults (bar group 1a), patients with healthy respiratory tract but with antibiotic therapy for extrapulmonary infection (bar group 1b), non-neutropenic intubated and mechanically ventilated ICU patients without antibiotic therapy (bar group 2a), non-neutropenic intubated and mechanically ventilated ICU patients with antibiotic therapy for extrapulmonary infection (bar group 2b), and patients with antibiotic therapy due to pneumonia (bar group 3b). Significant differences of average relative *Candida* abundance between groups are shown with bars representing the average *Candida* abundance and standard errors in given groups (* = p <0.05 for groups 2a, 2b, 3b versus 1a or 1b).

**Fig 4:**
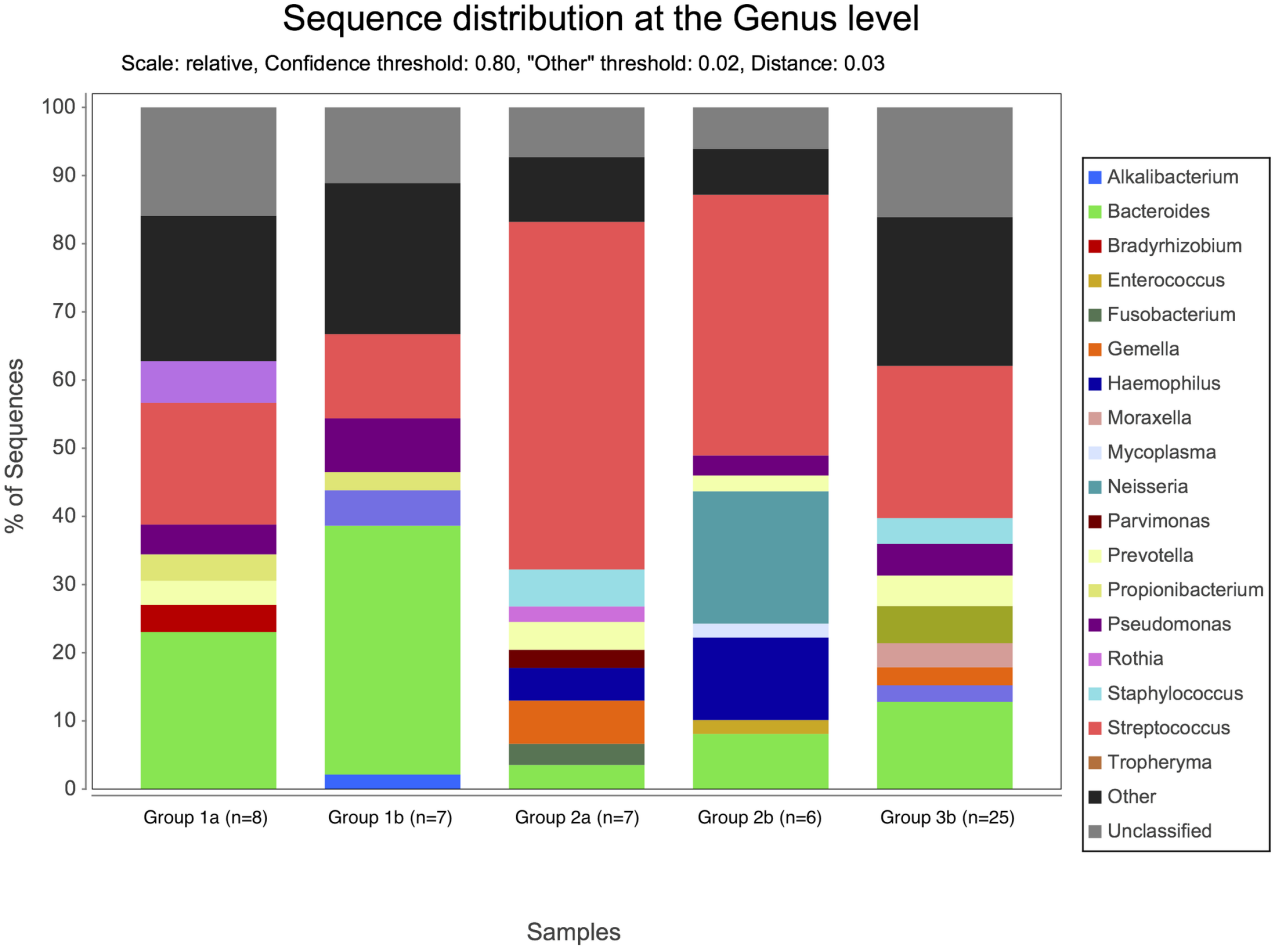
Relative abundance of bacterial microbiota in lower respiratory tract samples. Healthy adults (bar group 1a), patients with healthy respiratory tract but with antibiotic therapy for extrapulmonary infection (bar group 1b), non-neutropenic intubated and mechanically ventilated ICU patients without antibiotic therapy (bar group 2a), non-neutropenic intubated and mechanically ventilated ICU patients with antibiotic therapy for extrapulmonary infection (bar group 2b), and patients with antibiotic therapy due to pneumonia (bar group 3b). Bacteria are shown at genus level. By conventional culture bacteria were cultured in 5/8 (group 1a), 2/7 (group 1b), 7/7 (group 2a), 3/6 (group 2b), and 13/26 (group 3b) patients.

**Fig 5:**
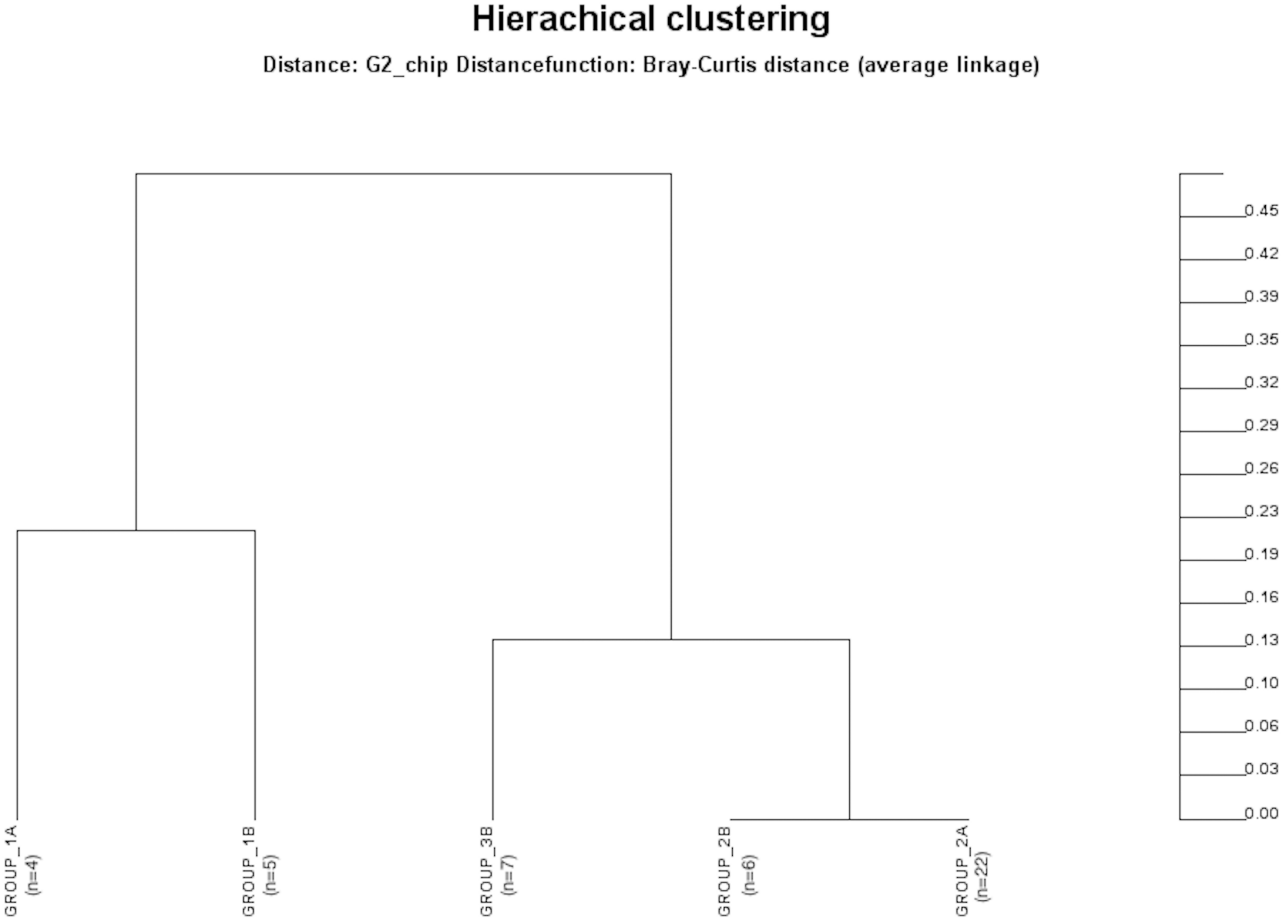
Bray-Curtis distances between study groups indicating differences in fungal species composition. Distance of zero indicates that groups are completely similar for every species. Distance of 1 indicates that groups are completely dissimilar and do not share any species. Group 1a, healthy adults; group 1b, healthy respiratory tract but with antibiotic therapy for extrapulmonary infection; group 2a, non-neutropenic intubated and mechanically ventilated ICU patients without antibiotic therapy; group 2b, non-neutropenic intubated and mechanically ventilated ICU patients with antibiotic therapy for extrapulmonary infection; group 3b, non-neutropenic intubated and mechanically ventilated ICU patients with antibiotic therapy due to pneumonia.

**Fig 6:**
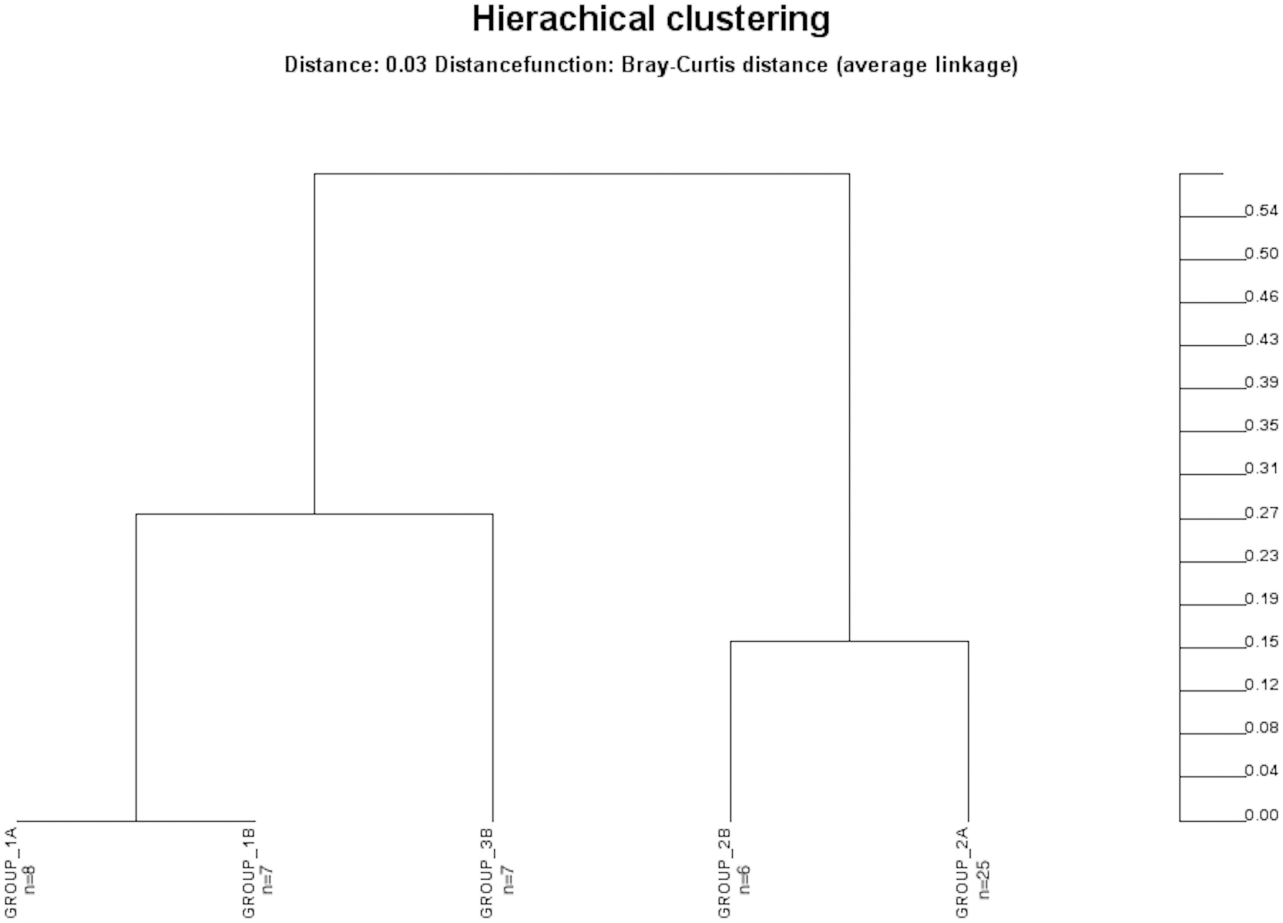
Bray-Curtis distances between study groups indicating differences in bacterial species composition. Distance of zero indicates that groups are completely similar for every species. Distance of 1 indicates that groups are completely dissimilar and do not share any species. Group 1a, healthy adults; group 1b, healthy respiratory tract but with antibiotic therapy for extrapulmonary infection; group 2a, non-neutropenic intubated and mechanically ventilated ICU patients without antibiotic therapy; group 2b, non-neutropenic intubated and mechanically ventilated ICU patients with antibiotic therapy for extrapulmonary infection; group 3b, non-neutropenic intubated and mechanically ventilated ICU patients with antibiotic therapy due to pneumonia.

**Fig 7:**
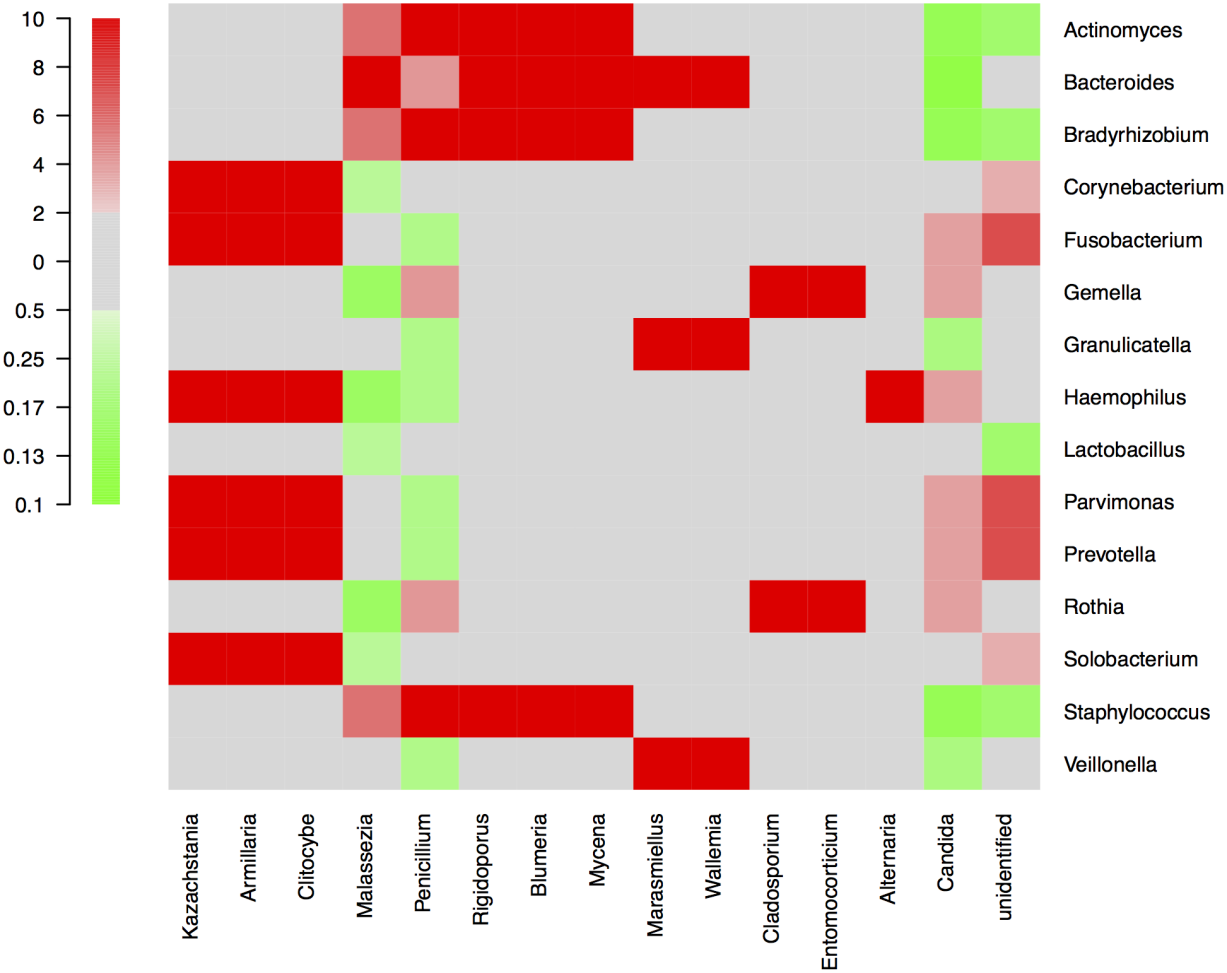
Associations/dissociations between bacteria and fungi (heatmaps). Relationships (association/dissociation) between bacteria and fungi in lower respiratory tract samples of non-neutropenic intubated and mechanically ventilated ICU patients without antibiotic therapy calculated as odds ratios and depicted as heatmap. An odds ratio above 2 was considered a positive association (in red), an odds ratio below 0.5 was interpreted as negative association (= dissociation, in green). The scale shows odds ratios.

**Fig 8:**
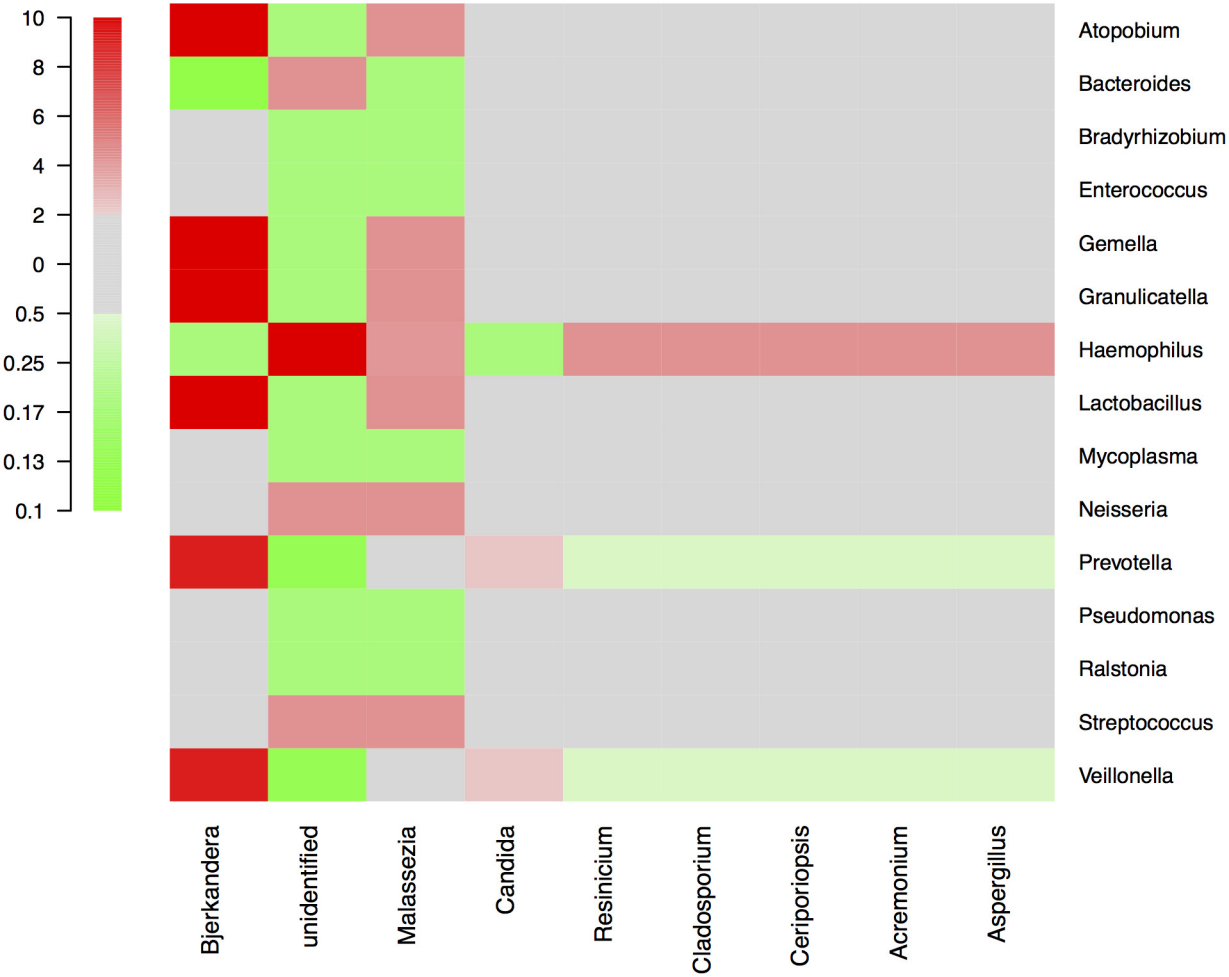
Associations/dissociations between bacteria and fungi (heatmaps). Relationships (association/dissociation) between bacteria and fungi in lower respiratory tract samples of non-neutropenic intubated and mechanically ventilated ICU patients with antibiotic therapy for extrapulmonary infection calculated as odds ratios and depicted as heatmap. An odds ratio above 2 was considered a positive association (in red), an odds ratio below 0.5 was interpreted as negative association (= dissociation, in green). The scale shows odds ratios.

**Fig 9:**
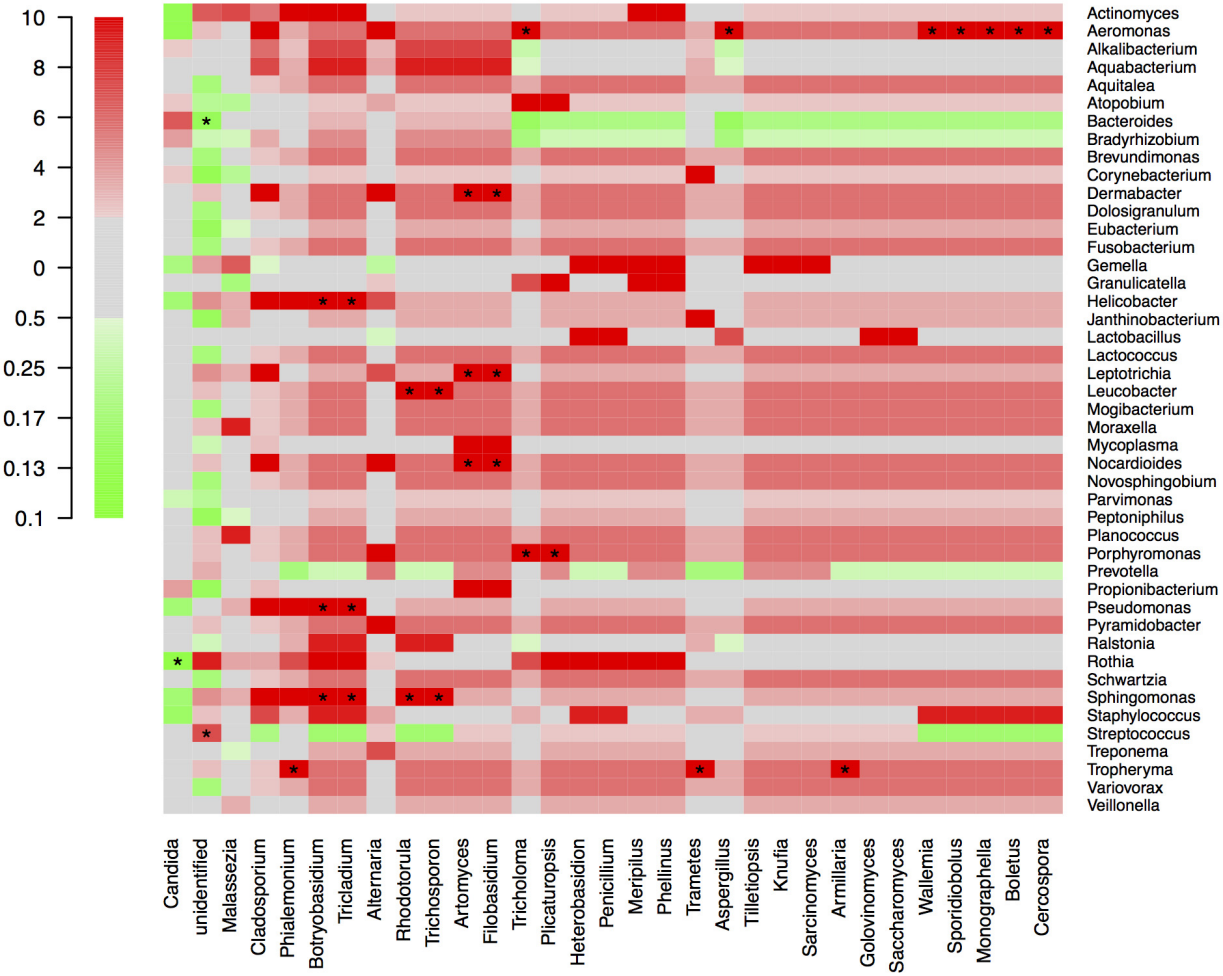
Associations/dissociations between bacteria and fungi (heatmaps). Relationships (association/dissociation) between bacteria and fungi in lower respiratory tract samples of non-neutropenic intubated and mechanically ventilated ICU patients with antibiotic therapy due to pneumonia calculated as odds ratios and depicted as heatmap. An odds ratio above 2 was considered a positive association (in red), an odds ratio below 0.5 was interpreted as negative association (= dissociation, in green). The scale shows odds ratios. Asterisks show significant differences (p<0.05; 95% confidence interval) for odds ratios (Fig 9).

**Fig 10:**
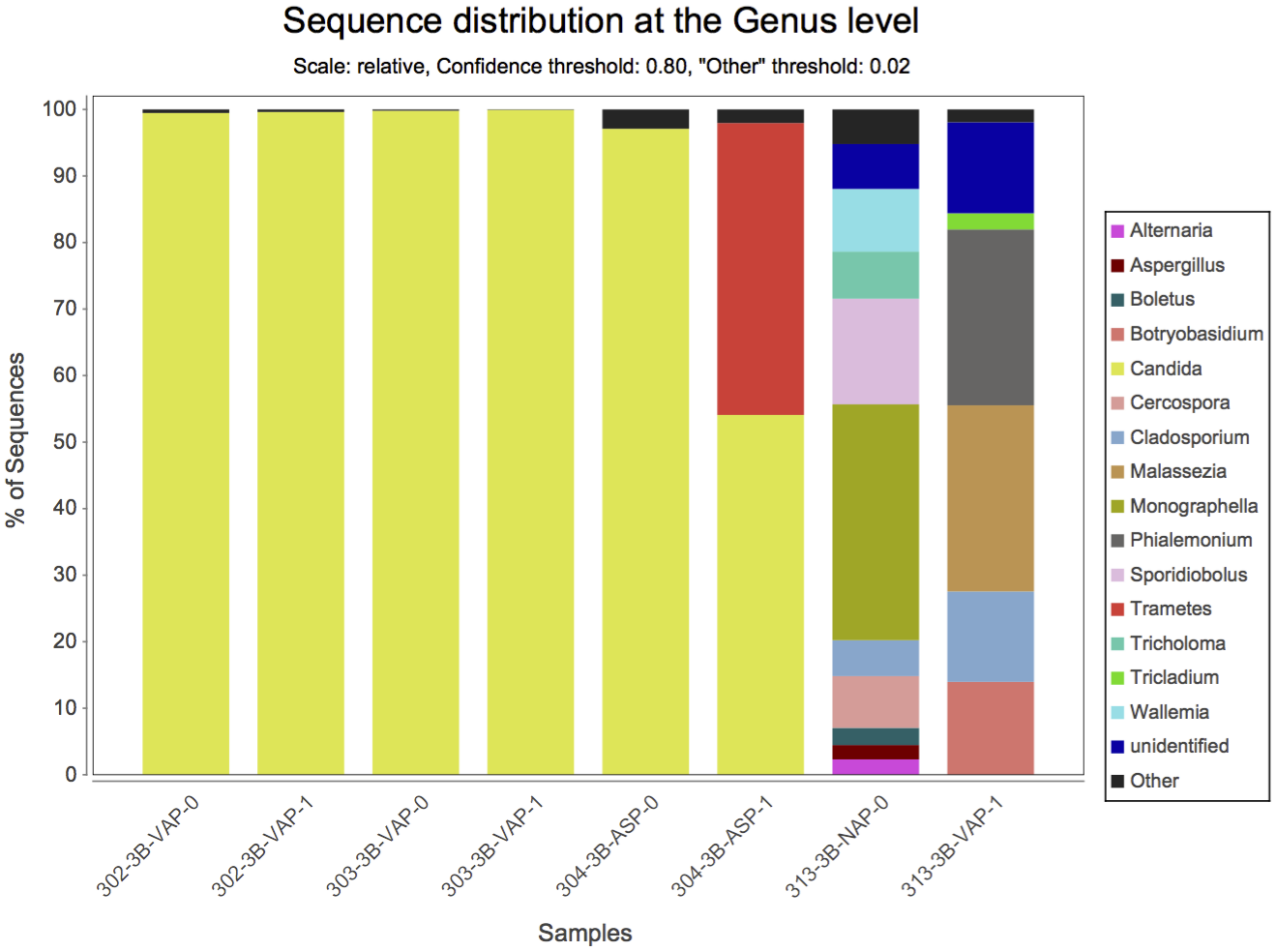
Relative abundance of fungal microbiota in consecutively sampled patients. Consecutive lower respiratory tract samples of 4 intubated and mechanically ventilated patients with pneumonia. Fungi are shown at genus level. Yellow bars represent *Candida* species. VAP = Ventilator associated pneumonia, ASP = aspiration pneumonia, NAP = nosocomial acquired pneumonia.

**Fig 11:**
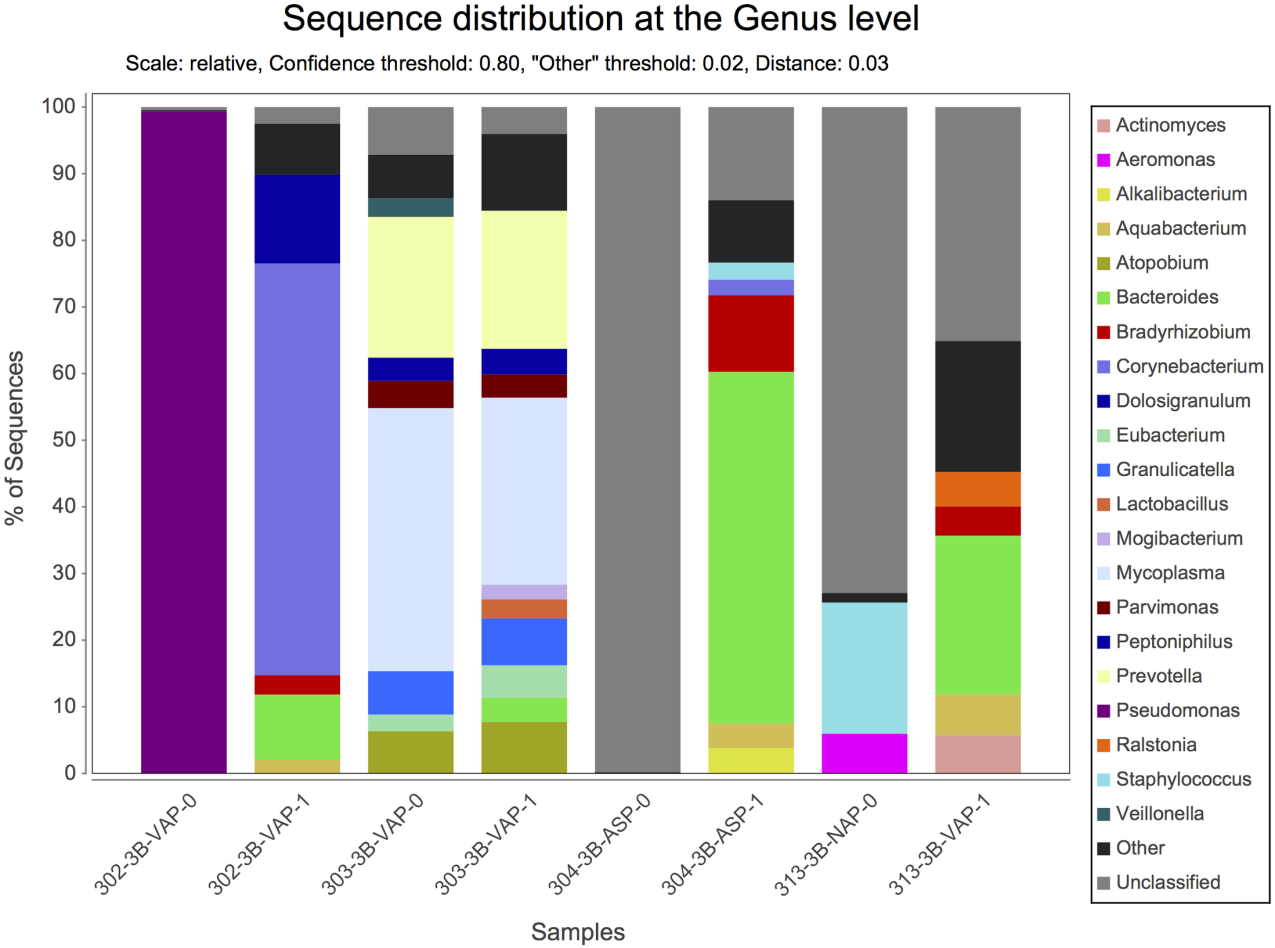
Relative abundance of bacterial microbiota in consecutively sampled patients. Consecutive lower respiratory tract samples of 4 intubated and mechanically ventilated patients with pneumonia. Bacteria are shown at genus level. Unclassified sequences in sample 304-3B-ASP-0 revealed *Escherichia/Shigella sp*. using a confidence threshold of 0.63. Comparison of these sequences with reference sequences using BLAST yielded uncultured *Shigella sp*. 16s rRNA genes. Unclassified sequences in sample 313-3B-VAP-0 revealed *Enterobacter sp*. using a confidence threshold of 0.63. Comparison of these sequences with reference sequences using BLAST yielded uncultured proteobacterium 16s rRNA genes. VAP = Ventilator associated pneumonia, ASP = aspiration pneumonia, NAP = nosocomial acquired pneumonia.

By NGS analysis the LRT of group 1a, 1b, 2a, 2b, and 3b patients contained 12, 10, 8, 5, 7 fungal genera, respectively (including taxa containing more than 2%; genera below 2% of sequences pooled). *Candida spp*. were detected in 0%, 0%, 50%, 63% and 73% of total fungal genera in the according groups, respectively (Fig 2). The differences of average relative *Candida* abundance between groups are shown in Fig 3 (p <0.05 for group 1a and 1b versus all other groups). The LRT of group 1a, 1b, 2a, 2b, and 3b patients further contained 9, 8, 11, 10, 11 bacterial genera (including taxa containing more than 2%; genera below 2% of sequences pooled) (Fig 4). Relative abundances for each fungal and bacterial taxonomic group for each sample are shown in the Supporting Information file (S1 and S2 Tables). Differences in fungal and bacterial microbiota between various groups (including fungal and bacterial richness, eveness and diversity indices) are shown in Figs 5 and 6, and in the Supporting Information file (S3–S6 Tables). Associations and dissociations between certain fungal and bacterial genera are shown in Figs 7–9 and in the Supporting Information file (S7–S11 Tables and S5 and S6 Figs). In group 2a the presence of *Candida spp*. was associated with *Fusobacteria sp*., *Gemella sp*., *Haemophilus sp*., *Parvimonas sp*., *Prevotella sp*., *Rothia sp*., and dissociated with *Actinomyces sp*., *Bacteroides sp*., *Bradyrhizobium sp*., *Granulicatella sp*., *Staphylococcus sp*. *and Veillonella sp*. In group 2b the presence of *Candida spp*. was associated with *Prevotella sp*. and *Veillonella sp*., and dissociated with *Haemophilus sp*. In group 3b the presence of *Candida spp*. was associated with *Atobium sp*., *Bacteroides sp*., *Bradyrhizobium sp*., *Corynebacterium sp*. *and Propionibacterium sp*., and dissociated with *Actinomyces sp*., *Aeromonas sp*., *Gemella sp*., *Helicobacter sp*., *Parvimonas sp*., *Pseudomonas sp*., *Rothia sp*., *Sphingomonas sp*., *Staphylococcus sp*. In summary, there was no common bacterial microbiota profile associated or dissociated with *Candida spp*. in LRT in the ICU groups and there were no associations or dissociations at all between *Candida spp*. and bacterial genera in group 1a and 1b. Fungal and bacterial communities of 4 patients with consecutive BAL samples are shown in Figs 10 and 11. Whereas fungal microbiota were stable as shown by analysis of first and second BAL sample in 3 of 4 patients bacterial communities differed in consecutive BAL samples in 3 of 4 patients.

Median kynurenine levels in LRT samples did not differ between patients from group 1a, 2b, and 3b, i.e. patients with and without pneumonia (median below the detection limit of 1 μM/L; one patient in group 2b had 1.21 μM/L and one patient in group 3 had 1.51 μM/L; p>0.05 for all comparisons). Since kynurenine is a metabolite of the essential amino acid tryptophan, we also calculated differences in tryptophan levels and a kynurenine/tryptophan ratio, to exclude dietary or ICU-treatment related influence (i.e., low kynurenine levels due to low tryptophan levels). There was no difference between group 2b and 3b in these calculations (p>0.05 for both comparisons). ICU patients from group 2b (median 6.6 μM/L, interquartile range [IQR] 1.4–25.8 μM/L) and 3b (median 7.9 μM/L, IQR 1–22.3 μM/L) had significantly higher tryptophan levels compared to healthy controls (median below the detection limit of 1μM/L) (p<0.001 for both comparisons).

## Discussion

Our data show that *Candida* is part of fungal microbiota of various intubated and mechanically ventilated ICU patients with and without antibiotic therapy and with and without pneumonia. *Candida* appears rapidly in the LRT after admission to an ICU even in patients without evidence of pneumonia. Some previously proposed risk factors for *Candida* colonization and infection like admission to and treatment on ICUs including intubation and mechanically ventilation shifted LRT fungal microbiota to *Candida spp*. dominated fungal profiles; however, antibiotic therapy did not. As shown previously, culture independent molecular assays can complement cultivation based findings and clearly extend culture results [8]. Our study is in line with previous studies confirming the non-sterility of the lung [8]. The shift towards *Candida spp*. dominated microbiota in ICU patients appeared to develop quickly, since intubated ICU patients without and with antibiotitic therapy were investigated on median day 2 and 3 after admission to ICU. The highest abundance of *Candida* (73% of all fungal genera) was detected in intubated and mechanically ventilated patients with various types of pneumonia treated with antibiotic therapy. However, the increase was not significantly different to the other ICU groups with and without antibiotic therapy and without pneumonia. There was no difference in fungal microbiota in certain pneumonia subtypes and relative abundance of *Candida spp*. in ASP and VAP compared to NAP (78%, 77% and 43% of all fungal genera; p>0.05 for all comparisons). In contrast to previous investigations we did not observe a loss of diversity by antibiotic treatment since subjects from group 1a (healthy adults) and 1b (patients with healthy respiratory tract but with antibiotic therapy for extrapulmonary infection) as well as 2a (non-neutropenic intubated and mechanically ventilated ICU patients without antibiotic therapy) and 2b (non-neutropenic intubated and mechanically ventilated ICU patients with antibiotic therapy for extrapulmonary infection) had similar Shannon diversity indices [16]. In our study every sample from LRT was obtained through endotracheal tubes even in healthy subjects. Therefore, contamination of samples from oral or pharyngeal bacterial and fungal microbiota during bronchoscopy or sampling is unlikely. The absence of *Candida spp*. within LRT fungal microbiota of healthy subjects (group 1a) resembles those of previous investigations [50]. In some previous studies reagents have been analyzed to assess the influence of reagents on mircobiota results from BALs [50]; however, in other studies this was not done [28]. Nevertheless, none of the studies investigating also reagents showed that *Candida* abundance reached >50% as has been found in our bronchoalveolar samples from ICU patients. Therefore, falsification of our BAL results by fungal content of reagents was very unlikely [50]. In addition, recent literature showed similar community compositions by 16s rRNA sequencing between endotracheal aspirate and BAL from single individuals [16]. However, we also compared EBS and BAL in intubated and mechanically ICU patients and obtained similar fungal microbiota compositions in both samples. *Candida* was absent in saline used for BAL. As *Candida* abundance did not increase in BAL samples compared to EBS and since *Candida* was not present in saline used for BAL the high abundance of *Candida* in our ICU patients can not be attributed to sampling technique or contamination by saline used for BAL.

In our patients the shift towards *Candida spp*. was not associated with invasive candidiasis as none of the included ICU patients of group 2 and 3 had candidemia or other invasive fungal disease. There was no association of *Candida* dominated LRT microbiota and intrahospital or 30 day overall mortality. However, this finding might be influenced by low sample size. Certainly, we do not believe that *Candida* colonization at all is of no relevance in ICU patients as it has been demonstrated as one major risk factor for invasive candidiasis [10].

There was no common bacterial microbiota profile associated or dissociated with *Candida spp*. as assessed by NGS. Previously, favorable growth conditions for *Pseudomonas aeruginosa* provided by *Candida albicans* and vice versa have been reported by investigating microbial interactions with conventional cultures [51–53]. Culture-independent methods provided a new perspectives to bacterial and fungal diversity. Since there was discordance between conventional culture based and recent molecular investigations of fungal microbiota previous studies using conventional fungal cultures solely are no longer reliable [7,8]. Thus, associations of *Candida spp*. in the LRT with other microorganisms, microbial interactions, worse clinical outcome and increased hospital mortality of *Candida* colonized ICU patients as shown in previous investigations using conventional *Candida* cultures are questionable. By NGS analysis we did not observe an association of *Pseudomonas sp*. and *Candida spp*. but found a dissociation in ICU patients with pneumonia.

In our study *Candida* isolates from candidemic patients as well as from colonized patients did not match with certain *Candida* clades as determined by phylogenetic MLST analysis. This indicates that not a single putative invasive *Candida* clade was responsible for invasive candidiasis in candidemic group 4 patients and strengthens the hypothesis that certain host factors are probably more important for development of invasive candidiasis [10].

Previously, we showed that *Candida* colonized ICU patients with and without pneumonia as well as healthy controls had lower serum IL-17A and kynurenine levels compared to patients with candidemia [12]. Increased kynurenine levels in vaginal lavage have been observed in a murine model of vulvovaginal candidiasis [54]. In our study kynurenine levels as well as kynurenine/tryptophan ratio in LRT did not differ between healthy controls and intubated and mechanically ventilated ICU patients with *Candida* positive cultures or *Candida* dominated fungal microbiota (with and without pneumonia) suggesting the absence of invasive pulmonary candidiasis in our subjects with *Candida* in LRT. IL-22 has been determined previously and showed no differences between patients with and without invasive candidiasis [12]. The (1–3)-ß-D Glucan test has a high potential to exclude invasive candidiasis based on its negative predictive value [11,49]. Twentytwo patients had negative (1–3)-ß-D Glucan tests despite presence of *Candida spp*. in LRT in 8 of these patients. Ten patients in pneumonia group 3b had evelevated serum (1–3)-ß-D Glucan tests. Of these patients 4 had *Candida* positive LRT cultures and 5 had *Candida* positive LRT fungal microbiota (of which 3 also had *Candida* positive cultures). However, all patients had significantly lower serum IL-17A values compared to candidemic patients suggesting *Candida* colonization in contrast to infection as described previously [12]. Additionally, the positive predictive value for detection of invasive candidiasis by (1–3)-ß-D Glucan test is only 72% in ICU patients [11] and we did not find invasive candidiasis by culture of blood, body fluids or sterile tissues (as clinically indicated). We therefore believe that these patients did not suffer from invasive candidiasis.

Previously, one autopsy study showed absence of *Candida* pneumonia in 77 patients with *Candida spp*. growth in tracheal aspirate and/or BAL cultures two weeks before death but another showed *Candida* pneumonia in 8% of 25 deceased ICU patients [2,3]. However, in a third autopsy study 4 investigators presented higly discordant results in assessment of histopathological samples from pneumonia patients. Histopathological examination as the proposed gold standard for diagnosis of *Candida* pneumonia seemed to suffer from high interobserver variability and standardization has been requested [55]. Transfer of data from autopsy analyses to the clinical setting suffers from several limitations including delayed necropsy, vague correlation of premortem cultures with postmortem histological examinations, missing postmortem cultures in many cases, and poor correlation of postmortem cultures with histopathological results in previous studies. In additon there is lack of clarity regarding the significance of *Candida spp*. in lung tissue accompanied by histologic changes of pneumonia but without the histologic demonstration of invading yeasts into lung tissue [2,3]. Additionally, lung biopsies are dangerous in intubated and mechanically ventilated patients due to imminent major side effects of this procedure and thus cannot be used in the clinical management of patients with suspected pulmonary *Candida* infection [3].

Based on our data including conventional culture, NGS, and host and fungal biomarker tests we conclude that *Candida* solely colonized the LRT of our 65 ICU patients. The presence of *Candida spp*. in the LRT rather reflected rapidly occurring LRT dysbiosis driven by ICU related factors than was associated with invasive candidiasis.

## Supporting Information

S1 Fig Fungal microbiota analysis (counts per OTU) of endobronchial secretion (EBS), bronchoalveloar lavage (BAL) and saline. Counts per OTU of fungal microbiota in lower respiratory tract samples (endobronchial secretion, EBS; bronchoalveolar lavage, BAL) and saline (NaCl). Results of BAL, EBS and saline are displayed for 5 intubated and mechanically ventilated ICU patients investigated for comparison of lower respiratory tract sampling techniques (EBS vs. BAL) and saline used for BAL in individual patients.
(PDF)

S2 Fig Fungal microbiota analysis (relative abundance) of endobronchial secretion (EBS), bronchoalveloar lavage (BAL) and saline. Relative abundance of fungal microbiota in lower respiratory tract samples (endobronchial secretion, EBS; bronchoalveolar lavage, BAL) and saline (NaCl). Results of BAL, EBS and saline are displayed for 5 intubated and mechanically ventilated ICU patients investigated for comparison of lower respiratory tract sampling techniques (EBS vs. BAL) and saline used for BAL in individual patients.
(PDF)

S3 Fig Bacterial microbiota analysis (counts per OTU) of endobronchial secretion (EBS), bronchoalveloar lavage (BAL) and saline. Counts per OTU of bacterial microbiota in lower respiratory tract samples (endobronchial secretion, EBS; bronchoalveolar lavage, BAL) and saline (NaCl). Results of BAL, EBS and saline are displayed for 5 intubated and mechanically ventilated ICU patients investigated for comparison of lower respiratory tract sampling techniques (EBS vs. BAL) and saline used for BAL in individual patients.
(PDF)

S4 Fig Bacterial microbiota analysis (relative abundance) of endobronchial secretion (EBS), bronchoalveloar lavage (BAL) and saline. Relative abundance of bacterial microbiota in lower respiratory tract samples (endobronchial secretion, EBS; bronchoalveolar lavage, BAL) and saline (NaCl). Results of BAL, EBS and saline are displayed for 5 intubated and mechanically ventilated ICU patients investigated for comparison of lower respiratory tract sampling techniques (EBS vs. BAL) and saline used for BAL in individual patients.
(PDF)

S5 Fig Associations/dissociations between bacteria and fungi (heatmaps) of patient group 1a. Relationships (association/dissociation) between bacteria and fungi in lower respiratory tract samples of healthy adults (group 1a) calculated as odds ratios and depicted as heatmap. An odds ratio above 2 was considered a positive association (in red), an odds ratio below 0.5 was interpreted as negative association (= dissociation, in green). The scale shows odds ratios. There were no associations or dissociations between *Candida spp*. and bacterial genera.
(PDF)

S6 Fig Associations/dissociations between bacteria and fungi (heatmaps) of patient group 1b. Relationships (association/dissociation) between bacteria and fungi in lower respiratory tract samples of patients with healthy respiratory tract but with antibiotic therapy for extrapulmonary infection (group 1b) calculated as odds ratios and depicted as heatmap. An odds ratio above 2 was considered a positive association (in red), an odds ratio below 0.5 was interpreted as negative association (= dissociation, in green). The scale shows odds ratios. There were no associations or dissociations between *Candida spp*. and bacterial genera in group 1b.
(PDF)

S1 Table Relative abundances for each fungal taxonomic group for each sample. (PDF)

S2 Table Relative abundances for each bacterial taxonomic group for each sample. (PDF)

S3 Table Differences in fungal microbiota of patient groups. Differences in fungal microbiota between various groups. 1a = healthy adults; 1b = patients with healthy respiratory tract but with antibiotic therapy for extrapulmonary infection; 2a = non-neutropenic intubated and mechanically ventilated ICU patients without antibiotic therapy; 2b = non-neutropenic intubated and mechanically ventilated ICU patients with antibiotic therapy for extrapulmonary infection; and 3b = non-neutropenic intubated and mechanically ventilated ICU patients with antibiotic therapy due to pneumonia. Tax = Taxa at genus level; G1 = comparative group 1; G2 = comparative group 2; raw Counts G1 = observed number of counts in comparative group 1. raw Counts G2 = observed number of counts in comparative group 2. cpm G1 = counts per million in comparative group 1. cpm G2 = counts per million in comparative group 2. log FC = log fold change. FDR = false diversity rate.
(PDF)

S4 Table Differences in bacterial microbiota of patient groups. Differences in bacterial microbiota between various groups. 1a = healthy adults; 1b = patients with healthy respiratory tract but with antibiotic therapy for extrapulmonary infection; 2a = non-neutropenic intubated and mechanically ventilated ICU patients without antibiotic therapy; 2b = non-neutropenic intubated and mechanically ventilated ICU patients with antibiotic therapy for extrapulmonary infection; and 3b = non-neutropenic intubated and mechanically ventilated ICU patients with antibiotic therapy due to pneumonia. Tax = Taxa at genus level; G1 = comparative group 1; G2 = comparative group 2; raw Counts G1 = observed number of counts in comparative group 1. raw Counts G2 = observed number of counts in comparative group 2. cpm G1 = counts per million in comparative group 1. cpm G2 = counts per million in comparative group 2. log FC = log fold change. FDR = false diversity rate.
(PDF)

S5 Table Fungal microbiota richness, eveness and diversity indices. Fungal microbiota richness, evenness and diversity of lower respiratory tract samples from study groups. Richness, Chao1 and ACE (Abundance-based Coverage Estimator) are different indicators of species richness. SDI (Shannon diversity index) and simpson are indicators of diversity. Kruskal-Wallis-Test was used for calculation of p-values (p-values <0.05 = significant). Groups: 1a = healthy adults, 1b = patients with healthy respiratory tract but with antibiotic therapy for extrapulmonary infection, 2a = non-neutropenic intubated and mechanically ventilated ICU patients without antibiotic therapy, 2b = non-neutropenic intubated and mechanically ventilated ICU patients with antibiotic therapy for extrapulmonary infection, and 3b = non-neutropenic intubated and mechanically ventilated ICU patients with antibiotic therapy due to pneumonia.
(PDF)

S6 Table Bacterial microbiota richness, eveness and diversity indices. Bacterial microbiota richness, evenness and diversity of lower respiratory tract samples from study groups. Richness, Chao1 and ACE (Abundance-based Coverage Estimator) are different indicators of species richness. SDI (Shannon diversity index) and simpson are indicators of diversity. Kruskal-Wallis-Test was used for calculation of p-values (p-values <0.05 = significant). Groups: 1a = healthy adults, 1b = patients with healthy respiratory tract but with antibiotic therapy for extrapulmonary infection, 2a = non-neutropenic intubated and mechanically ventilated ICU patients without antibiotic therapy, 2b = non-neutropenic intubated and mechanically ventilated ICU patients with antibiotic therapy for extrapulmonary infection, and 3b = non-neutropenic intubated and mechanically ventilated ICU patients with antibiotic therapy due to pneumonia.
(PDF)

S7 Table Associations/dissociations between bacteria and fungi (odds ratios), group 1a. Relationships (association/dissociation) between bacteria and fungi in lower respiratory tract samples of healthy adults (group 1a) calculated and depicted as odds ratios. An odds ratio above 2 was considered a positive association, an odds ratio below 0.5 was interpreted as negative association (= dissociation).
(PDF)

S8 Table Associations/dissociations between bacteria and fungi (odds ratios), group 1b. Relationships (association/dissociation) between bacteria and fungi in lower respiratory tract samples of patients with healthy respiratory tract but with antibiotic therapy for extrapulmonary infection (group 1b) calculated and depicted as odds ratios. An odds ratio above 2 was considered a positive association, an odds ratio below 0.5 was interpreted as negative association (= dissociation).
(PDF)

S9 Table Associations/dissociations between bacteria and fungi (odds ratios), group 2a. Relationships (association/dissociation) between bacteria and fungi in lower respiratory tract samples of non-neutropenic intubated and mechanically ventilated ICU patients without antibiotic therapy (group 2a) calculated and depicted as odds ratios. An odds ratio above 2 was considered a positive association, an odds ratio below 0.5 was interpreted as negative association (= dissociation).
(PDF)

S10 Table Associations/dissociations between bacteria and fungi (odds ratios), group 2b. Relationships (association/dissociation) between bacteria and fungi in lower respiratory tract samples of non-neutropenic intubated and mechanically ventilated ICU patients with antibiotic therapy for extrapulmonary infection (group 2b) calculated and depicted as odds ratios. An odds ratio above 2 was considered a positive association, an odds ratio below 0.5 was interpreted as negative association (= dissociation).
(PDF)

S11 Table Associations/dissociations between bacteria and fungi (odds ratios), group 3b. Relationships (association/dissociation) between bacteria and fungi in lower respiratory tract samples of non-neutropenic intubated and mechanically ventilated ICU patients with antibiotic therapy due to pneumonia (group 3b) calculated and depicted as odds ratios. An odds ratio above 2 was considered a positive association, an odds ratio below 0.5 was interpreted as negative association (= dissociation).
(PDF)
