# Supplementary figures and images for: Characterisation of Candida within the Mycobiome/Microbiome of the Lower Respiratory Tract of ICU Patients
Source: PLoS One. 2016 May 20;11(5):e0155033. doi: 10.1371/journal.pone.0155033 (PMC4874575; doi:10.1371/journal.pone.0155033)

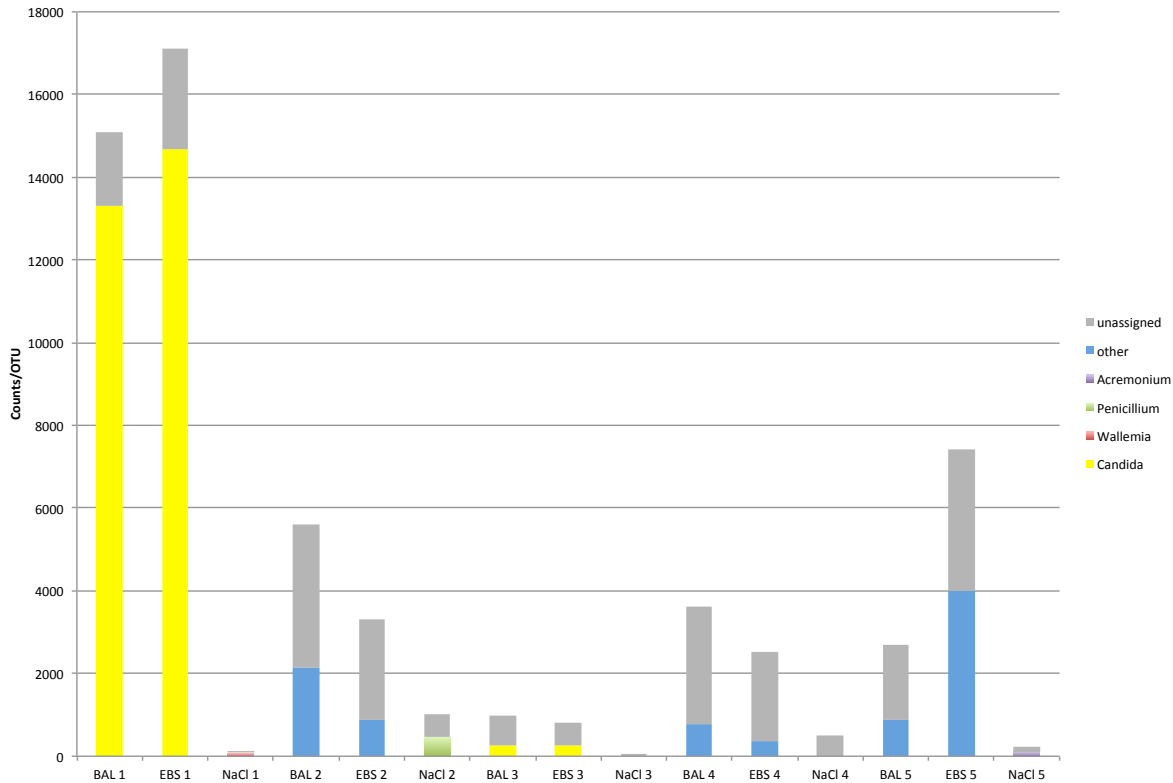

Supplement: S1 Fig — Counts per OTU of fungal microbiota in lower respiratory tract samples (endobronchial secretion, EBS; bronchoalveolar lavage, BAL) and saline (NaCl). Results of BAL, EBS and saline are displayed for 5 intubated and mechanically ventilated ICU patients investigated for comparison of lower respiratory tract sampling techniques (EBS vs. BAL) and saline used for BAL in individual patients. (PDF) [file pone.0155033.s001.pdf]

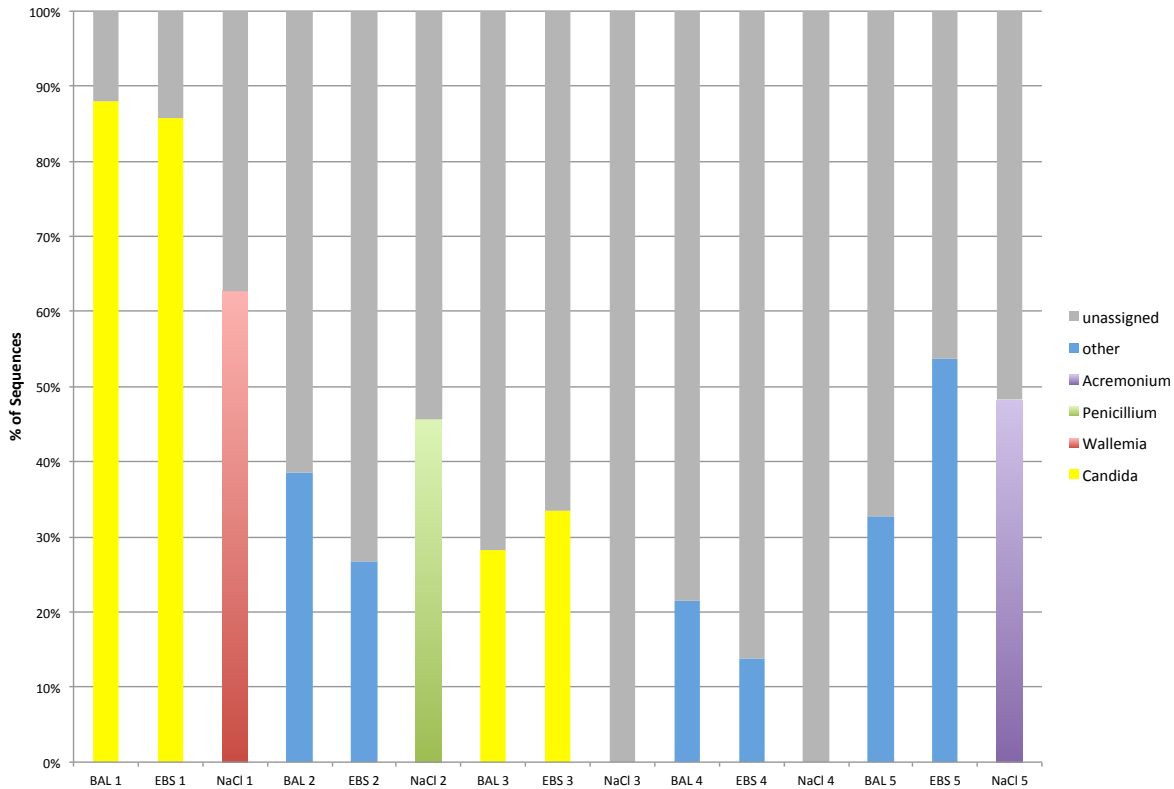

Supplement: S2 Fig — Relative abundance of fungal microbiota in lower respiratory tract samples (endobronchial secretion, EBS; bronchoalveolar lavage, BAL) and saline (NaCl). Results of BAL, EBS and saline are displayed for 5 intubated and mechanically ventilated ICU patients investigated for comparison of lower respiratory tract sampling techniques (EBS vs. BAL) and saline used for BAL in individual patients. (PDF) [file pone.0155033.s002.pdf]

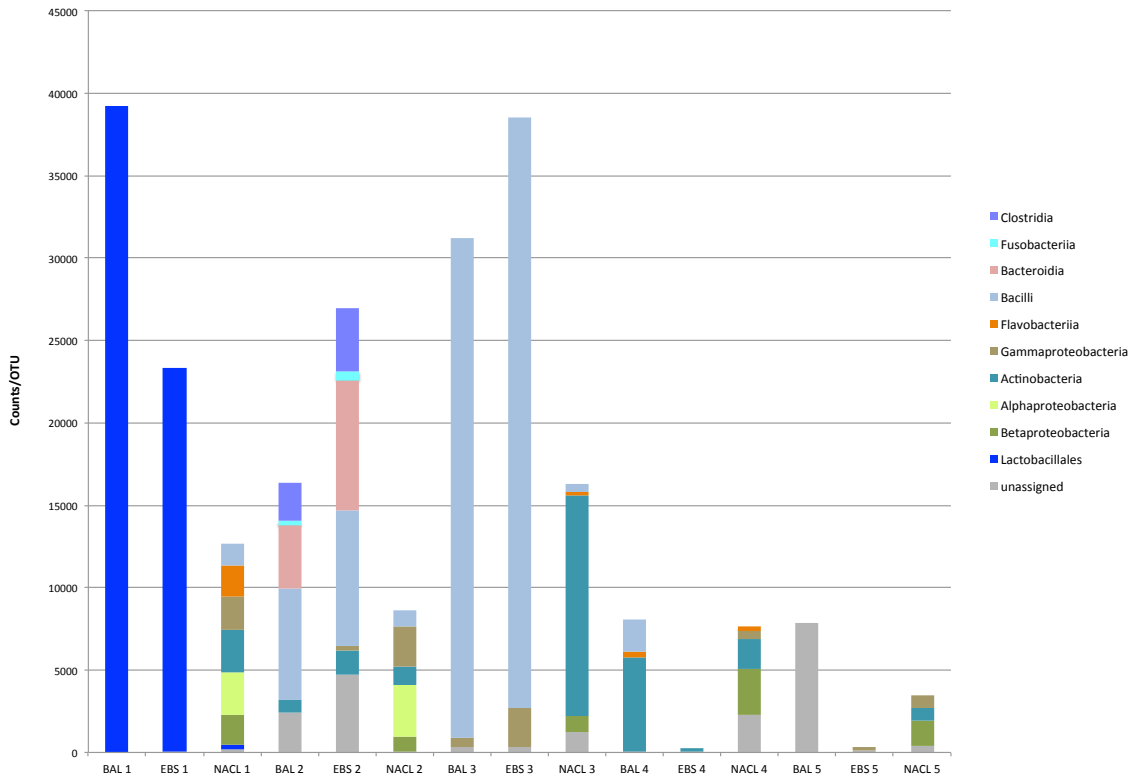

Supplement: S3 Fig — Counts per OTU of bacterial microbiota in lower respiratory tract samples (endobronchial secretion, EBS; bronchoalveolar lavage, BAL) and saline (NaCl). Results of BAL, EBS and saline are displayed for 5 intubated and mechanically ventilated ICU patients investigated for comparison of lower respiratory tract sampling techniques (EBS vs. BAL) and saline used for BAL in individual patients. (PDF) [file pone.0155033.s003.pdf]

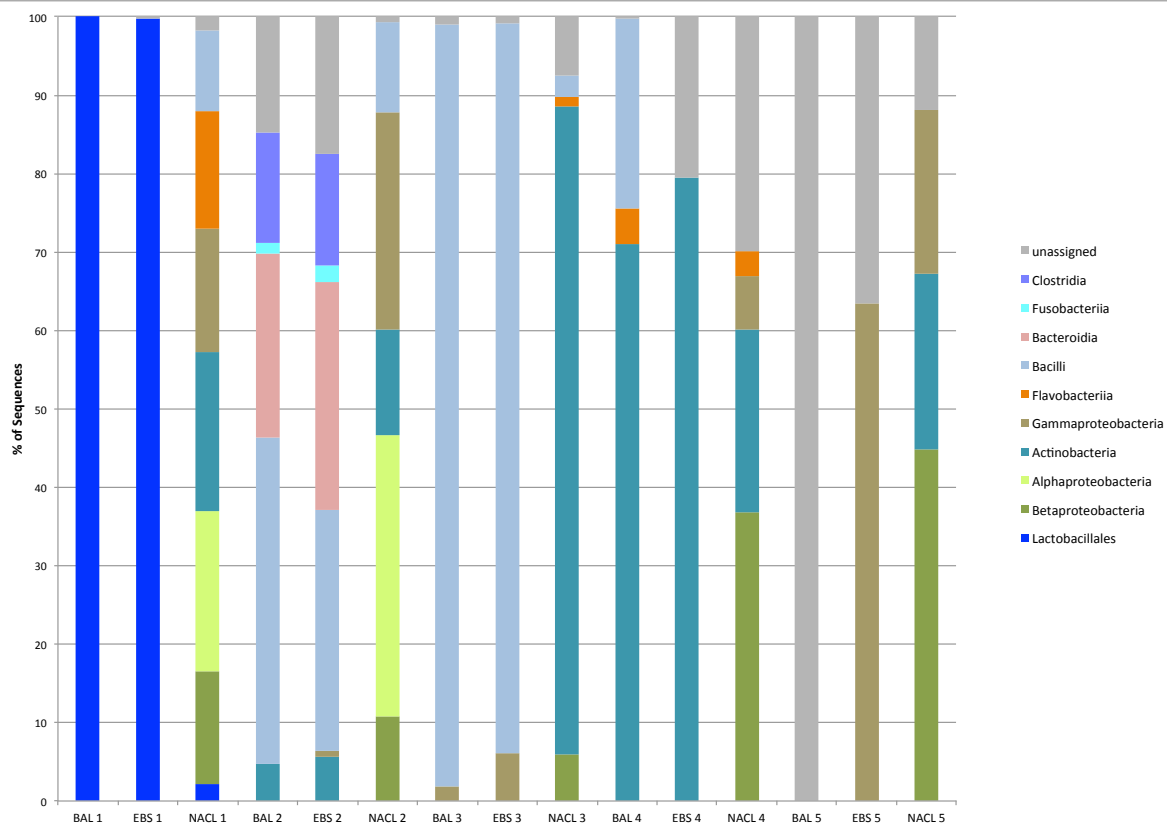

Supplement: S4 Fig — Relative abundance of bacterial microbiota in lower respiratory tract samples (endobronchial secretion, EBS; bronchoalveolar lavage, BAL) and saline (NaCl). Results of BAL, EBS and saline are displayed for 5 intubated and mechanically ventilated ICU patients investigated for comparison of lower respiratory tract sampling techniques (EBS vs. BAL) and saline used for BAL in individual patients. (PDF) [file pone.0155033.s004.pdf]

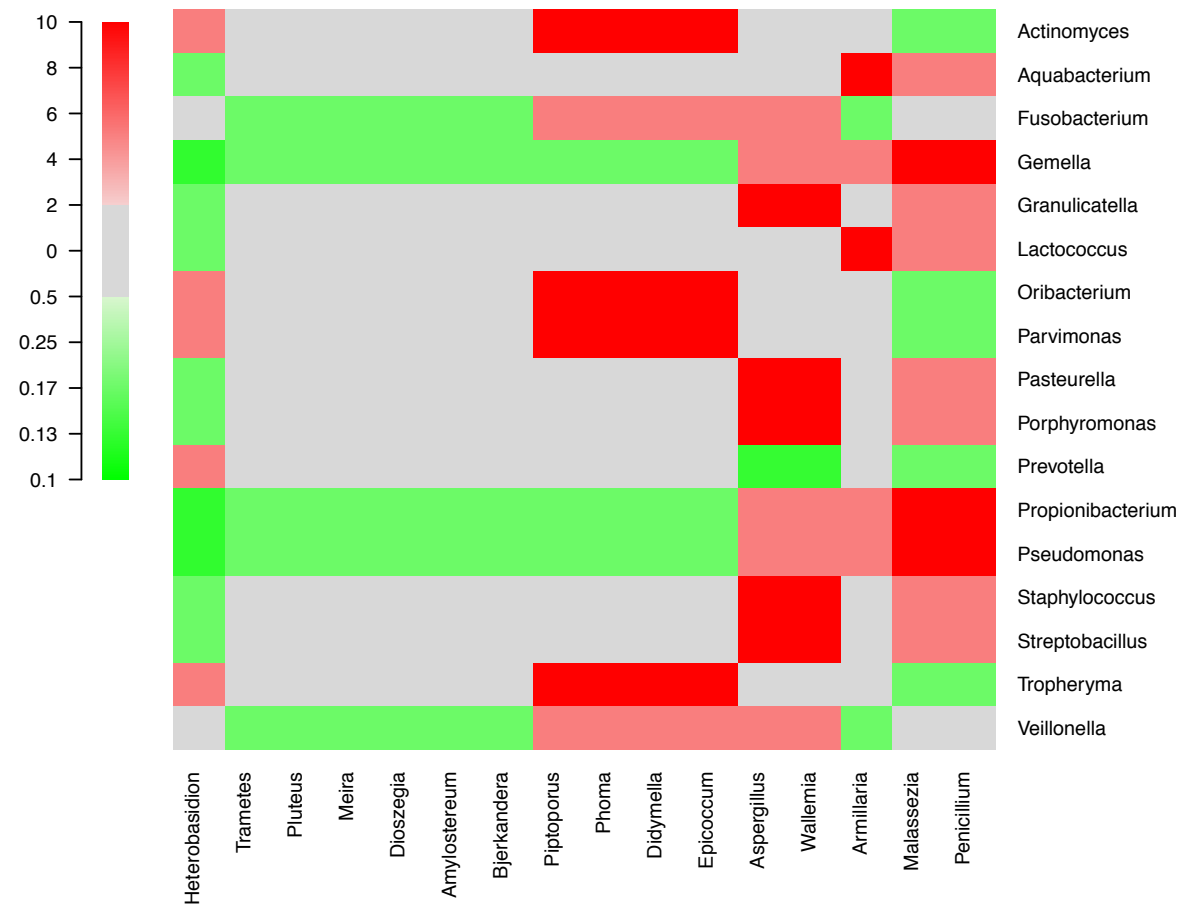

Supplement: S5 Fig — Relationships (association/dissociation) between bacteria and fungi in lower respiratory tract samples of healthy adults (group 1a) calculated as odds ratios and depicted as heatmap. An odds ratio above 2 was considered a positive association (in red), an odds ratio below 0.5 was interpreted as negative association (= dissociation, in green). The scale shows odds ratios. There were no associations or dissociations between Candida spp. and bacterial genera. (PDF) [file pone.0155033.s005.pdf]

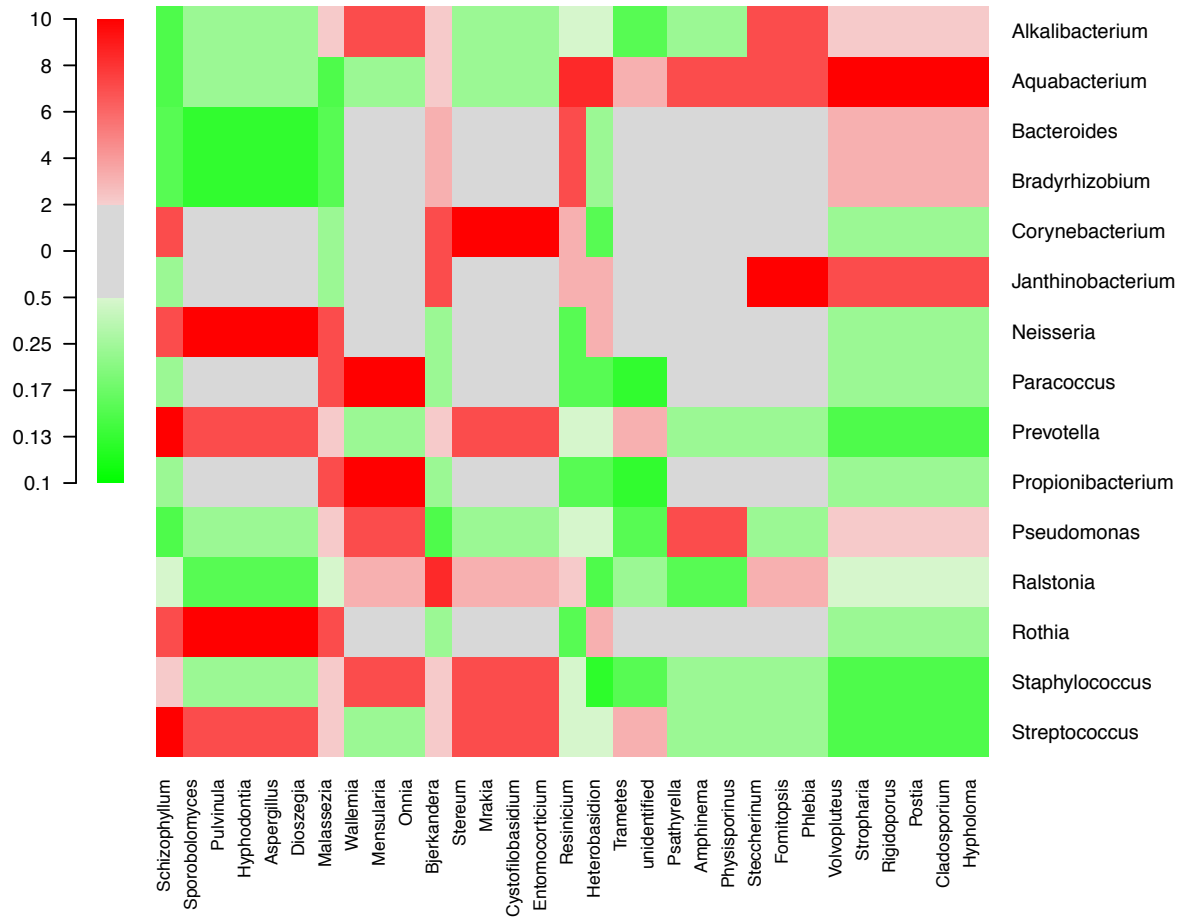

Supplement: S6 Fig — Relationships (association/dissociation) between bacteria and fungi in lower respiratory tract samples of patients with healthy respiratory tract but with antibiotic therapy for extrapulmonary infection (group 1b) calculated as odds ratios and depicted as heatmap. An odds ratio above 2 was considered a positive association (in red), an odds ratio below 0.5 was interpreted as negative association (= dissociation, in green). The scale shows odds ratios. There were no associations or dissociations between Candida spp. and bacterial genera in group 1b. (PDF) [file pone.0155033.s006.pdf]
