# Supplementary material for: Characterisation of Candida within the Mycobiome/Microbiome of the Lower Respiratory Tract of ICU Patients
Source: PLoS One. 2016 May 20;11(5):e0155033. doi: 10.1371/journal.pone.0155033 (PMC4874575; doi:10.1371/journal.pone.0155033)
Supplement: S3 Table — Differences in fungal microbiota between various groups. 1a = healthy adults; 1b = patients with healthy respiratory tract but with antibiotic therapy for extrapulmonary infection; 2a = non-neutropenic intubated and mechanically ventilated ICU patients without antibiotic therapy; 2b = non-neutropenic intubated and mechanically ventilated ICU patients with antibiotic therapy for extrapulmonary infection; and 3b = non-neutropenic intubated and mechanically ventilated ICU patients with antibiotic therapy due to pneumonia. Tax = Taxa at genus level; G1 = comparative group 1; G2 = comparative group 2; raw Counts G1 = observed number of counts in comparative group 1. raw Counts G2 = observed number of counts in comparative group 2. cpm G1 = counts per million in comparative group 1. cpm G2 = counts per million in comparative group 2. log FC = log fold change. FDR = false diversity rate. (PDF) [file pone.0155033.s009.pdf]

TAX	G1	G2	number of samples G1	number of samples G2	rawCounts G1	rawCounts G2	cpm G1	cpm G2	logFC	FDR
Ophiocordyceps	1a	1b	4	5	1665	0	59830.9	142.9	8.54	4,37E-03
Amylostereum	1a	1b	4	5	1017	0	36251.4	142.9	7.82	5,94E-05
Bjerkandera	1a	1b	4	5	911	0	32487.9	142.9	7.66	5,94E-05
Pluteus	1a	1b	4	5	941	0	33553	142.9	7.71	5,94E-05
Clitocybe	1a	1b	4	5	335	0	12152.3	142.9	6.24	1,01E-03
Schizophyllum	1a	1b	4	5	0	705	143.1	20255.6	-6.98	1,32E-03
Mensularia	1a	1b	4	5	0	1257	143.1	36310.6	-7.82	1,55E-03
Piptoporus	1a	1b	4	5	208	0	7599.6	142.9	5.57	3,17E-03
Hypholoma	1a	1b	4	5	0	343	143.1	9928	-5.95	5,76E-03
Postia	1a	1b	4	5	0	341	143.1	9879.3	-5.94	0.000169073
Physisporinus	1a	1b	4	5	0	294	143.05	8537.4	-5.73	0.000205362
Resinicium	1a	1b	4	5	0	915	143.1	26240.3	-7.35	0.000205362
Porotheleum	1a	1b	4	5	0	267	143.1	7752.8	-5.59	0.000215561
Stereum	1a	1b	4	5	0	270	143.1	7852.1	-5.61	0.000215561
Grandinia	1a	1b	4	5	0	249	143.1	7239.7	-5.49	0.000237996
Heterobasidion	1a	1b	4	5	1949	0	69341.9	142.9	8.75	0.000329445
Mycena	1a	1b	4	5	0	148	143.1	4368.7	-4.77	0.000769069
Peniophora	1a	1b	4	5	0	138	143.1	4083.1	-4.67	0.000870724
Penicillium	1a	1b	4	5	4829	0	173255.9	142.9	10.07	0.00151559
Aspergillus	1a	1b	4	5	0	294	143.1	8522.3	-5.73	0.021478365
Ophiocordyceps	1a	2a	4	7	1665	0	59830.9	143.5	8.53	1,04E-07
Amylostereum	1a	2a	4	7	1017	0	36251.4	143.5	7.81	2,80E-07
Bjerkandera	1a	2a	4	7	911	0	32487.9	143.5	7.65	2,80E-07
Pluteus	1a	2a	4	7	941	0	33553	143.5	7.7	2,80E-07
Clitocybe	1a	2a	4	7	335	0	12152.3	143.5	6.24	1,63E-05
Piptoporus	1a	2a	4	7	208	0	7599.6	143.5	5.56	1,28E-04
Dioszegia	1a	2a	4	7	498	0	17824.4	143.5	6.79	3,10E-04
Rigidoporus	1a	2a	4	7	0	4639	143.1	95173	-9.21	2,32E-03
Heterobasidion	1a	2a	4	7	1949	0	69341.9	143.5	8.75	3,15E-03
Psathyrella	1a	2a	4	7	210	0	7671.3	143.5	5.57	3,15E-03
Candida	1a	2a	4	7	0	27777	143.1	570528.2	-11.79	9,08E-03
Kazachstania	1a	2a	4	7	0	687	143.1	14129.6	-6.46	0.000239284

TAX	G1	G2	number of samples G1	number of samples G2	rawCounts G1	rawCounts G2	cpm G1	cpm G2	logFC	FDR
Blumeria	1a	2a	4	7	0	393	143.1	8194.1	-5.67	0.000586765
Resinicium	1a	2a	4	7	0	166	143.1	3523	-4.46	0.015378769
Ophiocordyceps	1a	2b	4	6	1665	0	59830.9	143.7	8.53	2,07E-06
Amylostereum	1a	2b	4	6	1017	0	36251.4	143.7	7.81	3,96E-06
Bjerkandera	1a	2b	4	6	911	0	32487.9	143.7	7.65	3,96E-06
Pluteus	1a	2b	4	6	941	0	33553	143.7	7.7	3,96E-06
Clitocybe	1a	2b	4	6	335	0	12152.3	143.7	6.23	1,25E-04
Piptoporus	1a	2b	4	6	208	0	7599.56	143.7	5.56	7,13E-04
Dioszegia	1a	2b	4	6	498	0	17824.4	143.7	6.79	1,65E-03
Candida	1a	2b	4	6	0	37544	143.1	899888.8	-12.45	6,97E-03
Heterobasidion	1a	2b	4	6	1949	0	69341.9	143.7	8.75	0.000125398
Psathyrella	1a	2b	4	6	210	0	7671.3	143.7	5.57	0.000125398
Penicillium	1a	2b	4	6	4829	0	173255.9	143.7	10.07	0.000815484
Lenzites	1a	2b	4	6	0	2763	143.1	66176.8	-8.68	0.003938745
Acremonium	1a	2b	4	6	0	67	143.1	1750.2	-3.46	0.015041624
Ophiocordyceps	1a	3b	4	26	1665	0	59830.9	143.5	8.53	4,07E-28
Amylostereum	1a	3b	4	26	1017	0	36251.4	143.5	7.81	3,70E-25
Pluteus	1a	3b	4	26	941	0	33553	143.5	7.7	8,19E-25
Bjerkandera	1a	3b	4	26	911	0	32487.9	143.5	7.65	1,01E-24
Clitocybe	1a	3b	4	26	335	0	12152.3	143.5	6.24	7,47E-18
Dioszegia	1a	3b	4	26	498	0	17824.4	143.5	6.79	9,92E-15
Piptoporus	1a	3b	4	26	208	0	7599.6	143.5	5.56	1,50E-14
Psathyrella	1a	3b	4	26	210	0	7671.3	143.5	5.57	1,31E-10
Candida	1a	3b	4	26	0	180978	143.1	999515.8	-12.6	3,64E-03
Heterobasidion	1a	3b	4	26	1949	461	69341.9	2674.8	4.69	3,64E-03
Plectosphaerella	1a	3b	4	26	0	5909	143.1	32328.8	-7.65	0.018895928
Phialemonium	1a	3b	4	26	0	2410	143.1	13418.9	-6.38	0.022625527
Sporidiobolus	1a	3b	4	26	0	2752	143.1	15133.2	-6.56	0.02333967
Nakaseomyces	1a	3b	4	26	0	1603	143.1	9006.4	-5.81	0.024921938
Cladosporium	1a	3b	4	26	0	1188	143.1	6627.1	-5.37	0.026722561
Rhodotorula	1a	3b	4	26	0	1290	143.1	7245.1	-5.5	0.026722561
Cercospora	1a	3b	4	26	0	1355	143.1	7524	-5.55	0.027103785

TAX	G1	G2	number of samples G1	number of samples G2	rawCounts G1	rawCounts G2	cpm G1	cpm G2	logFC	FDR
Tricholoma	1a	3b	4	26	0	1147	143.1	6391	-5.32	0.028552211
Alternaria	1a	3b	4	26	0	686	143.1	3929.4	-4.62	0.033485111
Artomyces	1a	3b	4	26	0	480	143.1	2790.8	-4.12	0.039854176
Lenzites	1a	3b	4	26	0	862	143.1	4883.7	-4.93	0.039854176
Tilletiopsis	1a	3b	4	26	0	578	143.1	3331.3	-4.38	0.039854176
Trametes	1a	3b	4	26	0	596	143.1	3405.3	-4.41	0.039854176
Trichosporon	1a	3b	4	26	0	516	143.1	2971.7	-4.22	0.039854176
Boletus	1a	3b	4	26	0	438	143.1	2529.2	-3.98	0.040958592
Mensularia	1b	2a	5	7	1257	0	36310.6	143.5	7.81	2,30E-06
Schizophyllum	1b	2a	5	7	705	0	20255.6	143.5	6.97	2,30E-06
Hypholoma	1b	2a	5	7	343	0	9928	143.5	5.94	2,84E-05
Postia	1b	2a	5	7	341	0	9879.3	143.5	5.94	1,41E-04
Physisporinus	1b	2a	5	7	294	0	8537.3	143.5	5.73	1,93E-04
Porotheleum	1b	2a	5	7	267	0	7752.8	143.5	5.59	1,93E-04
Rigidoporus	1b	2a	5	7	0	4639	142.9	95173	-9.21	1,93E-04
Stereum	1b	2a	5	7	270	0	7852.1	143.5	5.61	1,93E-04
Grandinia	1b	2a	5	7	249	0	7239.7	143.5	5.49	2,29E-04
Dioszegia	1b	2a	5	7	479	0	13795.1	143.5	6.42	4,45E-04
Candida	1b	2a	5	7	0	27777	142.9	570528.2	-11.79	9,18E-04
Mycena	1b	2a	5	7	148	0	4368.7	143.5	4.76	1,47E-03
Psathyrella	1b	2a	5	7	274	0	7952.3	143.5	5.63	1,47E-03
Peniophora	1b	2a	5	7	138	0	4083.1	143.5	4.67	1,86E-03
Kazachstania	1b	2a	5	7	0	687	142.9	14129.6	-6.46	2,69E-03
Blumeria	1b	2a	5	7	0	393	142.9	8194.1	-5.67	8,51E-03
Aspergillus	1b	2a	5	7	294	0	8522.3	143.5	5.72	0.002266652
Resinicium	1b	2a	5	7	915	166	26240.3	3523	2.89	0.034343139
Armillaria	1b	2a	5	7	0	91	142.9	1996.1	-3.65	0.042179007
Penicillium	1b	2a	5	7	0	331	142.9	6924	-5.43	0.047788798
Mensularia	1b	2b	5	6	1257	0	36310.6	143.7	7.81	2,85E-05
Schizophyllum	1b	2b	5	6	705	0	20255.6	143.7	6.97	2,85E-05
Hypholoma	1b	2b	5	6	343	0	9928	143.7	5.94	2,21E-04
Postia	1b	2b	5	6	341	0	9879.3	143.7	5.94	8,76E-04

TAX	G1	G2	number of samples G1	number of samples G2	rawCounts G1	rawCounts G2	cpm G1	cpm G2	logFC	FDR
Candida	1b	2b	5	6	0	37544	142.9	899888.8	-12.45	9,79E-04
Physisporinus	1b	2b	5	6	294	0	8537.4	143.7	5.73	9,79E-04
Porotheleum	1b	2b	5	6	267	0	7752.8	143.7	5.59	1,04E-03
Stereum	1b	2b	5	6	270	0	7852.1	143.7	5.61	1,04E-03
Grandinia	1b	2b	5	6	249	0	7239.7	143.7	5.49	1,18E-03
Dioszegia	1b	2b	5	6	479	0	13795.1	143.7	6.42	2,21E-03
Mycena	1b	2b	5	6	148	0	4368.7	143.7	4.76	6,08E-03
Psathyrella	1b	2b	5	6	274	0	7952.3	143.7	5.62	6,08E-03
Peniophora	1b	2b	5	6	138	0	4083.1	143.7	4.67	7,29E-03
Lenzites	1b	2b	5	6	0	2763	142.9	66176.8	-8.69	0.000849149
Acremonium	1b	2b	5	6	0	67	142.9	1750.2	-3.46	0.005131978
Resinicium	1b	2b	5	6	915	62	26240.3	1630.3	3.99	0.005614114
Mensularia	1b	3b	5	26	1257	0	36310.6	143.5	7.81	4,03E-23
Schizophyllum	1b	3b	5	26	705	0	20255.6	143.5	6.97	5,62E-23
Hypholoma	1b	3b	5	26	343	0	9928	143.5	5.95	6,93E-18
Resinicium	1b	3b	5	26	915	0	26240.3	143.5	7.35	1,05E-16
Postia	1b	3b	5	26	341	0	9879.3	143.5	5.94	6,64E-16
Physisporinus	1b	3b	5	26	294	0	8537.4	143.5	5.73	4,91E-15
Stereum	1b	3b	5	26	270	0	7852.1	143.5	5.61	1,48E-14
Porotheleum	1b	3b	5	26	267	0	7752.8	143.5	5.59	1,56E-14
Dioszegia	1b	3b	5	26	479	0	13795.1	143.5	6.42	3,33E-14
Grandinia	1b	3b	5	26	249	0	7239.7	143.5	5.49	3,51E-14
Psathyrella	1b	3b	5	26	274	0	7952.3	143.5	5.63	6,43E-12
Mycena	1b	3b	5	26	148	0	4368.7	143.5	4.76	6,10E-11
Peniophora	1b	3b	5	26	138	0	4083.1	143.5	4.67	1,55E-10
Candida	1b	3b	5	26	0	180978	142.9	999515.8	-12.6	1,82E-04
Plectosphaerella	1b	3b	5	26	0	5909	142.9	32328.8	-7.65	0.004320611
Phialemonium	1b	3b	5	26	0	2410	142.9	13418.9	-6.38	0.005667623
Sporidiobolus	1b	3b	5	26	0	2752	142.9	15133.2	-6.56	0.006127111
Nakaseomyces	1b	3b	5	26	0	1603	142.9	9006.4	-5.81	0.006910283
Rhodotorula	1b	3b	5	26	0	1290	142.9	7245.1	-5.5	0.007793962
Cladosporium	1b	3b	5	26	0	1188	142.9	6627.1	-5.37	0.008033622

TAX	G1	G2	number of samples G1	number of samples G2	rawCounts G1	rawCounts G2	cpm G1	cpm G2	logFC	FDR
Cercospora	1b	3b	5	26	0	1355	142.9	7524	-5.55	0.008392155
Penicillium	1b	3b	5	26	0	4022	142.9	22241.6	-7.11	0.008775744
Tricholoma	1b	3b	5	26	0	1147	142.9	6391	-5.32	0.008800804
Alternaria	1b	3b	5	26	0	686	142.9	3929.4	-4.62	0.011008507
Tilletiopsis	1b	3b	5	26	0	578	142.9	3331.3	-4.38	0.01430971
Trametes	1b	3b	5	26	0	596	142.9	3405.3	-4.41	0.01430971
Artomyces	1b	3b	5	26	0	480	142.9	2790.8	-4.13	0.015496942
Lenzites	1b	3b	5	26	0	862	142.9	4883.7	-4.93	0.015496942
Trichosporon	1b	3b	5	26	0	516	142.9	2971.7	-4.22	0.015496942
Boletus	1b	3b	5	26	0	438	142.9	2529.2	-3.99	0.016683947
Phellinus	1b	3b	5	26	0	308	142.9	1849.7	-3.54	0.023295236
Heterobasidion	1b	3b	5	26	0	461	142.9	2674.8	-4.07	0.024061504
Saccharomyces	1b	3b	5	26	0	253	142.9	1532.7	-3.27	0.027781545
Rigidoporus	2a	2b	7	6	4639	0	95173	143.7	9.2	1,02E-04
Kazachstania	2a	2b	7	6	687	0	14129.6	143.7	6.45	3,12E-03
Blumeria	2a	2b	7	6	393	0	8194.1	143.7	5.67	8,94E-03
Lenzites	2a	2b	7	6	0	2763	143.5	66176.8	-8.68	0.000214656
Acremonium	2a	2b	7	6	0	67	143.5	1750.2	-3.45	0.003029642
Rigidoporus	2a	3b	7	26	4639	0	95173	143.5	9.2	4,75E-24
Kazachstania	2a	3b	7	26	687	0	14129.6	143.5	6.45	3,60E-16
Blumeria	2a	3b	7	26	393	0	8194.1	143.5	5.67	1,47E-13
Resinicium	2a	3b	7	26	166	0	3523	143.5	4.46	2,65E-06
Plectosphaerella	2a	3b	7	26	0	5909	143.5	32328.8	-7.65	0.001649791
Phialemonium	2a	3b	7	26	0	2410	143.5	13418.9	-6.38	0.002193337
Sporidiobolus	2a	3b	7	26	0	2752	143.5	15133.2	-6.55	0.002252112
Nakaseomyces	2a	3b	7	26	0	1603	143.54	9006.4	-5.8	0.002525007
Rhodotorula	2a	3b	7	26	0	1290	143.5	7245.1	-5.49	0.00284686
Cladosporium	2a	3b	7	26	0	1188	143.5	6627.1	-5.36	0.002858013
Cercospora	2a	3b	7	26	0	1355	143.5	7524	-5.55	0.002924242
Tricholoma	2a	3b	7	26	0	1147	143.5	6391	-5.31	0.003228367
Alternaria	2a	3b	7	26	0	686	143.5	3929.4	-4.61	0.004320767
Tilletiopsis	2a	3b	7	26	0	578	143.5	3331.3	-4.37	0.005937968

TAX	G1	G2	number of samples G1	number of samples G2	rawCounts G1	rawCounts G2	cpm G1	cpm G2	logFC	FDR
Trametes	2a	3b	7	26	0	596	143.5	3405.3	-4.41	0.005937968
Artomyces	2a	3b	7	26	0	480	143.5	2790.8	-4.12	0.006381345
Lenzites	2a	3b	7	26	0	862	143.5	4883.7	-4.92	0.006381345
Trichosporon	2a	3b	7	26	0	516	143.5	2971.7	-4.21	0.006381345
Boletus	2a	3b	7	26	0	438	143.5	2529.2	-3.98	0.006997761
Aspergillus	2a	3b	7	26	0	503	143.5	2890	-4.17	0.01021091
Phellinus	2a	3b	7	26	0	308	143.5	1849.7	-3.53	0.01044253
Heterobasidion	2a	3b	7	26	0	461	143.5	2674.8	-4.06	0.010686799
Saccharomyces	2a	3b	7	26	0	253	143.5	1532.7	-3.26	0.013167422
Oidium	2a	3b	7	26	0	134	143.5	879.3	-2.47	0.036988761
Acremonium	2b	3b	6	26	67	0	1750.2	143.5	3.45	1,76E-04
Resinicium	2b	3b	6	26	62	0	1630.3	143.5	3.35	0.000424599
Lenzites	2b	3b	6	26	2763	862	66176.8	4883.7	3.76	0.004453133
Plectosphaerella	2b	3b	6	26	0	5909	143.7	32328.8	-7.64	0.005943417
Phialemonium	2b	3b	6	26	0	2410	143.7	13418.9	-6.38	0.006920473
Sporidiobolus	2b	3b	6	26	0	2752	143.7	15133.2	-6.55	0.006920473
Nakaseomyces	2b	3b	6	26	0	1603	143.7	9006.4	-5.8	0.007328063
Cercospora	2b	3b	6	26	0	1355	143.7	7524	-5.54	0.007510045
Cladosporium	2b	3b	6	26	0	1188	143.7	6627.1	-5.36	0.007510045
Penicillium	2b	3b	6	26	0	4022	143.7	22241.6	-7.1	0.007510045
Rhodotorula	2b	3b	6	26	0	1290	143.7	7245.1	-5.49	0.007510045
Tricholoma	2b	3b	6	26	0	1147	143.7	6391	-5.31	0.007510045
Alternaria	2b	3b	6	26	0	686	143.7	3929.4	-4.61	0.009532083
Tilletiopsis	2b	3b	6	26	0	578	143.7	3331.3	-4.37	0.012327595
Trametes	2b	3b	6	26	0	596	143.7	3405.3	-4.4	0.012327595
Trichosporon	2b	3b	6	26	0	516	143.7	2971.7	-4.21	0.013295315
Artomyces	2b	3b	6	26	0	480	143.7	2790.8	-4.11	0.013552464
Boletus	2b	3b	6	26	0	438	143.7	2529.2	-3.98	0.014526367
Phellinus	2b	3b	6	26	0	308	143.7	1849.7	-3.53	0.02122753
Heterobasidion	2b	3b	6	26	0	461	143.7	2674.8	-4.06	0.021538389
Saccharomyces	2b	3b	6	26	0	253	143.7	1532.7	-3.26	0.02541457
