# Supplementary material for: Characterisation of Candida within the Mycobiome/Microbiome of the Lower Respiratory Tract of ICU Patients
Source: PLoS One. 2016 May 20;11(5):e0155033. doi: 10.1371/journal.pone.0155033 (PMC4874575; doi:10.1371/journal.pone.0155033)
Supplement: S4 Table — Differences in bacterial microbiota between various groups. 1a = healthy adults; 1b = patients with healthy respiratory tract but with antibiotic therapy for extrapulmonary infection; 2a = non-neutropenic intubated and mechanically ventilated ICU patients without antibiotic therapy; 2b = non-neutropenic intubated and mechanically ventilated ICU patients with antibiotic therapy for extrapulmonary infection; and 3b = non-neutropenic intubated and mechanically ventilated ICU patients with antibiotic therapy due to pneumonia. Tax = Taxa at genus level; G1 = comparative group 1; G2 = comparative group 2; raw Counts G1 = observed number of counts in comparative group 1. raw Counts G2 = observed number of counts in comparative group 2. cpm G1 = counts per million in comparative group 1. cpm G2 = counts per million in comparative group 2. log FC = log fold change. FDR = false diversity rate. (PDF) [file pone.0155033.s010.pdf]

TAX	G1	G2	number of samples G1	number of samples G2	rawCounts G1	rawCounts G2	cpm G1	cpm G2	logFC	FDR
Alkalibacterium	1a	1b	8	7	0	179	246.1	6491	-4.55	0.006587457
Aquabacterium	1a	1b	8	7	129	0	4081.2	254.4	3.85	0.046411043
Cloacibacterium	1a	1b	8	7	202	0	6501.5	254.4	4.51	0.000543804
Fusobacterium	1a	1b	8	7	368	0	11285.7	254.4	5.31	0.00276609
Neisseria	1a	1b	8	7	0	223	246.14	8649.4	-4.96	0.001250022
Paracoccus	1a	1b	8	7	0	200	246.1	7147.4	-4.69	0.000128723
Porphyromonas	1a	1b	8	7	131	0	4140.6	254.4	3.87	0.001250022
Streptobacillus	1a	1b	8	7	129	0	4081.1	254.4	3.85	0.001250022
Tropheryma	1a	1b	8	7	1858	0	57783.2	254.4	7.66	0.002310563
Veillonella	1a	1b	8	7	199	0	6162.2	254.4	4.44	0.030029159
Aquabacterium	1a	2a	8	7	129	0	4081.2	257.9	3.83	0.034158426
Bradyrhizobium	1a	2a	8	7	1006	0	31605.9	257.9	6.77	0.001380188
Cloacibacterium	1a	2a	8	7	202	0	6501.5	257.9	4.5	0.000386939
Corynebacterium	1a	2a	8	7	0	178	246.1	6392.7	-4.53	0.020233122
Gemella	1a	2a	8	7	0	1397	246.1	53917.6	-7.6	0.000783874
Haemophilus	1a	2a	8	7	0	1114	246.1	41286	-7.21	1,67E-04
Parvimonas	1a	2a	8	7	0	614	246.1	21846	-6.3	0.000776181
Porphyromonas	1a	2a	8	7	131	0	4140.6	257.9	3.85	0.000834175
Propionibacterium	1a	2a	8	7	1023	0	31245.5	257.9	6.75	0.000834175
Pseudomonas	1a	2a	8	7	1073	0	32942.7	257.9	6.83	0.010782243
Rothia	1a	2a	8	7	0	513	246.1	19578.4	-6.14	0.001042342
Solobacterium	1a	2a	8	7	0	259	246.1	9184.3	-5.05	2,09E-03
Streptobacillus	1a	2a	8	7	129	0	4081.2	257.9	3.83	0.000834175
Tropheryma	1a	2a	8	7	1858	0	57783.2	257.9	7.64	0.001380188
Cloacibacterium	1a	2b	8	6	202	0	6501.5	260.5	4.48	0.000971012
Enterococcus	1a	2b	8	6	0	566	246.1	25889.4	-6.54	9,06E-06
Fusobacterium	1a	2b	8	6	368	0	11285.7	260.5	5.35	0.004815494
Gemella	1a	2b	8	6	0	144	246.1	6206.2	-4.49	0.02902072
Granulicatella	1a	2b	8	6	0	113	246.1	4926.2	-4.16	0.01882617
Haemophilus	1a	2b	8	6	0	3358	246.1	147558.7	-9.05	7,58E-07
Lactobacillus	1a	2b	8	6	0	88	246.1	3894	-3.82	0.019204809

TAX	G1	G2	number of samples G1	number of samples G2	rawCounts G1	rawCounts G2	cpm G1	cpm G2	logFC	FDR
Mycoplasma	1a	2b	8	6	0	566	246.1	24054.5	-6.44	0.003002042
Neisseria	1a	2b	8	6	0	5393	246.1	226976.1	-9.67	7,58E-07
Porphyromonas	1a	2b	8	6	131	0	4140.6	260.5	3.84	0.002523728
Propionibacterium	1a	2b	8	6	1023	0	31245.5	260.5	6.74	0.002523728
Streptobacillus	1a	2b	8	6	129	0	4081.1	260.5	3.82	0.002523728
Tropheryma	1a	2b	8	6	1858	0	57783.2	260.5	7.63	0.004057751
Aeromonas	1a	3b	8	30	0	231	246.1	2373.2	-3.11	0.007807056
Alkalibacterium	1a	3b	8	30	0	772	246.1	6582.7	-4.57	0.001780624
Atopobium	1a	3b	8	30	0	779	246.1	6449.1	-4.54	0.001666673
Cloacibacterium	1a	3b	8	30	202	0	6501.5	257.41	4.5	1,02E-09
Corynebacterium	1a	3b	8	30	0	1384	246.1	12791.1	-5.53	0.001872128
Dolosigranulum	1a	3b	8	30	0	299	246.1	2965.2	-3.43	0.005158768
Eubacterium	1a	3b	8	30	0	254	246.1	2268.7	-3.05	0.008066541
Fusobacterium	1a	3b	8	30	368	117	11285.7	1274.9	3.12	0.000990035
Gemella	1a	3b	8	30	0	3461	246.14	30271.8	-6.77	0.000990035
Granulicatella	1a	3b	8	30	0	1179	246.1	9739.6	-5.14	0.001666673
Lactobacillus	1a	3b	8	30	0	423	246.1	3877.2	-3.81	0.00428154
Lactococcus	1a	3b	8	30	0	712	246.1	6243.7	-4.5	0.001872128
Leptotrichia	1a	3b	8	30	0	421	246.1	3630.1	-3.72	0.003199833
Moraxella	1a	3b	8	30	0	4793	246.1	44157.9	-7.31	0.000608469
Mycoplasma	1a	3b	8	30	0	7350	246.1	61378	-7.79	0.000408875
Nocardioides	1a	3b	8	30	0	903	246.1	7714.3	-4.8	0.001780624
Novosphingobium	1a	3b	8	30	0	1052	246.1	9102.4	-5.04	0.001666673
Parvimonas	1a	3b	8	30	0	685	246.1	5973	-4.43	0.002527882
Peptoniphilus	1a	3b	8	30	0	313	246.1	2736.9	-3.32	0.005072451
Planococcus	1a	3b	8	30	0	813	246.1	6681	-4.6	0.001872128
Porphyromonas	1a	3b	8	30	131	0	4140.6	257.4	3.85	2,26E-07
Propionibacterium	1a	3b	8	30	1023	377	31245.5	3427.1	3.18	0.001666673
Ralstonia	1a	3b	8	30	0	552	246.1	4676.3	-4.08	0.002527882
Rothia	1a	3b	8	30	0	1076	246.1	9004.1	-5.02	0.001872128
Streptobacillus	1a	3b	8	30	129	0	4081.1	257.4	3.83	2,26E-07

TAX	G1	G2	number of samples G1	number of samples G2	rawCounts G1	rawCounts G2	cpm G1	cpm G2	logFC	FDR
Treponema	1a	3b	8	30	0	208	246.1	1975.1	-2.85	0.010542488
Ureaplasma	1a	3b	8	30	0	168	246.1	1718.4	-2.65	0.01403704
Actinomyces	1b	2a	7	7	0	274	254.4	10131	-5.15	0.00935575
Alkalibacterium	1b	2a	7	7	179	0	6491	257.9	4.49	0.009091023
Bradyrhizobium	1b	2a	7	7	984	0	35585.9	257.9	6.94	0.00161755
Fusobacterium	1b	2a	7	7	0	662	254.4	23335.7	-6.35	0.000750756
Gemella	1b	2a	7	7	0	1397	254.4	53917.6	-7.56	0.001584108
Haemophilus	1b	2a	7	7	0	1114	254.4	41286	-7.17	1,14E-03
Neisseria	1b	2a	7	7	223	0	8649.4	257.9	4.9	0.001584108
Paracoccus	1b	2a	7	7	200	0	7147.4	257.9	4.63	0.000183762
Parvimonas	1b	2a	7	7	0	614	254.4	21846	-6.26	0.001584108
Propionibacterium	1b	2a	7	7	369	0	12972	257.9	5.49	0.004835428
Pseudomonas	1b	2a	7	7	1413	0	52918.9	257.9	7.51	0.006436752
Rothia	1b	2a	7	7	0	513	254.4	19578.4	-6.1	0.002553789
Solobacterium	1b	2a	7	7	0	259	254.4	9184.3	-5.01	9,55E-03
Veillonella	1b	2a	7	7	0	155	254.4	5843.1	-4.36	0.023360772
Alkalibacterium	1b	2b	7	6	179	0	6491	260.5	4.48	0.026025231
Enterococcus	1b	2b	7	6	0	566	254.4	25889.4	-6.5	1,12E-04
Granulicatella	1b	2b	7	6	0	113	254.4	4926.2	-4.11	0.038644426
Haemophilus	1b	2b	7	6	0	3358	254.4	147558.7	-9.01	9,84E-06
Lactobacillus	1b	2b	7	6	0	88	254.4	3894	-3.78	0.038644426
Mycoplasma	1b	2b	7	6	0	566	254.4	24054.5	-6.4	0.010489211
Neisseria	1b	2b	7	6	223	5393	8649.4	226976.1	-4.71	0.003833176
Paracoccus	1b	2b	7	6	200	0	7147.4	260.5	4.62	0.000665127
Propionibacterium	1b	2b	7	6	369	0	12972	260.5	5.48	0.014785926
Veillonella	1b	2b	7	6	0	266	254.4	11790.3	-5.37	0.013583014
Actinomyces	1b	3b	7	30	0	806	254.4	6660.4	-4.55	0.009793697
Aeromonas	1b	3b	7	30	0	231	254.4	2373.2	-3.07	0.016674468
Aquabacterium	1b	3b	7	30	0	1251	254.4	10611.8	-5.22	0.004713894
Atopobium	1b	3b	7	30	0	779	254.4	6449.1	-4.5	0.004713894
Dolosigranulum	1b	3b	7	30	0	299	254.4	2965.2	-3.39	0.011656177

TAX	G1	G2	number of samples G1	number of samples G2	rawCounts G1	rawCounts G2	cpm G1	cpm G2	logFC	FDR
Eubacterium	1b	3b	7	30	0	254	254.4	2268.7	-3.01	0.017000522
Gemella	1b	3b	7	30	0	3461	254.4	30271.8	-6.73	0.003717055
Granulicatella	1b	3b	7	30	0	1179	254.4	9739.6	-5.09	0.004713894
Lactobacillus	1b	3b	7	30	0	423	254.4	3877.2	-3.77	0.010081442
Lactococcus	1b	3b	7	30	0	712	254.4	6243.7	-4.45	0.005165113
Leptotrichia	1b	3b	7	30	0	421	254.4	3630.1	-3.68	0.008442569
Moraxella	1b	3b	7	30	0	4793	254.4	44157.9	-7.27	0.002249067
Mycoplasma	1b	3b	7	30	0	7350	254.4	61378	-7.74	0.00173512
Neisseria	1b	3b	7	30	223	0	8649.4	257.4	4.91	2,76E-07
Nocardioides	1b	3b	7	30	0	903	254.4	7714.3	-4.76	0.00495346
Novosphingobium	1b	3b	7	30	0	1052	254.4	9102.4	-5	0.004713894
Paracoccus	1b	3b	7	30	200	0	7147.4	257.4	4.64	1,91E-10
Parvimonas	1b	3b	7	30	0	685	254.4	5973	-4.39	0.006917691
Peptoniphilus	1b	3b	7	30	0	313	254.4	2736.9	-3.28	0.011656177
Planococcus	1b	3b	7	30	0	813	254.4	6681	-4.55	0.005165113
Rothia	1b	3b	7	30	0	1076	254.4	9004.1	-4.98	0.005165113
Treponema	1b	3b	7	30	0	208	254.4	1975.1	-2.81	0.021373762
Tropheryma	1b	3b	7	30	0	2402	254.4	22478.6	-6.3	0.004713894
Ureaplasma	1b	3b	7	30	0	168	254.4	1718.4	-2.61	0.027337866
Veillonella	1b	3b	7	30	0	344	254.4	2975.4	-3.4	0.031125267
Actinomyces	2a	2b	7	6	274	0	10131	260.5	5.12	0.021571253
Bradyrhizobium	2a	2b	7	6	0	214	257.9	9256.8	-5	0.020787971
Enterococcus	2a	2b	7	6	0	566	257.9	25889.4	-6.48	1,19E-04
Fusobacterium	2a	2b	7	6	662	0	23335.7	260.5	6.32	0.002377062
Granulicatella	2a	2b	7	6	0	113	257.9	4926.2	-4.1	0.030215699
Lactobacillus	2a	2b	7	6	0	88	257.9	3894	-3.76	0.030725796
Mycoplasma	2a	2b	7	6	0	566	257.9	24054.5	-6.38	0.007972966
Neisseria	2a	2b	7	6	0	5393	257.9	226976.1	-9.61	1,89E-05
Parvimonas	2a	2b	7	6	614	0	21846	260.49	6.23	0.004772005
Pseudomonas	2a	2b	7	6	0	802	257.9	33975.7	-6.87	0.015562541
Rothia	2a	2b	7	6	513	0	19578.4	260.49	6.07	0.007972966

TAX	G1	G2	number of samples G1	number of samples G2	rawCounts G1	rawCounts G2	cpm G1	cpm G2	logFC	FDR
Solobacterium	2a	2b	7	6	259	0	9184.3	260.5	4.98	0.000282563
Staphylococcus	2a	2b	7	6	1260	0	45659.9	260.5	7.29	0.011519721
Aeromonas	2a	3b	7	30	0	231	257.9	2373.2	-3.05	0.01655732
Alkalibacterium	2a	3b	7	30	0	772	257.9	6582.7	-4.51	0.004021618
Aquabacterium	2a	3b	7	30	0	1251	257.9	10611.8	-5.2	0.00402138
Atopobium	2a	3b	7	30	0	779	257.9	6449.1	-4.49	0.00402138
Bradyrhizobium	2a	3b	7	30	0	2662	257.9	21985.7	-6.25	0.003264647
Dolosigranulum	2a	3b	7	30	0	299	257.9	2965.2	-3.37	0.011534184
Eubacterium	2a	3b	7	30	0	254	257.9	2268.7	-2.99	0.016920429
Fusobacterium	2a	3b	7	30	662	117	23335.7	1274.9	4.16	9,84E-04
Granulicatella	2a	3b	7	30	0	1179	257.9	9739.6	-5.08	0.00402138
Haemophilus	2a	3b	7	30	1114	0	41286	257.4	7.16	3,07E-18
Lactobacillus	2a	3b	7	30	0	423	257.9	3877.2	-3.75	0.00989275
Lactococcus	2a	3b	7	30	0	712	257.9	6243.7	-4.44	0.004675571
Leptotrichia	2a	3b	7	30	0	421	257.9	3630.1	-3.66	0.007799998
Moraxella	2a	3b	7	30	0	4793	257.9	44157.9	-7.25	0.001844733
Mycoplasma	2a	3b	7	30	0	7350	257.9	61378	-7.73	0.00133393
Nocardioides	2a	3b	7	30	0	903	257.9	7714.3	-4.74	0.004021618
Novosphingobium	2a	3b	7	30	0	1052	257.9	9102.4	-4.98	0.00402138
Peptoniphilus	2a	3b	7	30	0	313	257.9	2736.9	-3.26	0.011534184
Planococcus	2a	3b	7	30	0	813	257.9	6680.98	-4.54	0.004529865
Propionibacterium	2a	3b	7	30	0	377	257.9	3427.1	-3.58	0.020662298
Pseudomonas	2a	3b	7	30	0	5862	257.9	54538	-7.56	0.00402138
Ralstonia	2a	3b	7	30	0	552	257.9	4676.3	-4.02	0.006090376
Solobacterium	2a	3b	7	30	259	0	9184.3	257.4	5	1,99E-12
Treponema	2a	3b	7	30	0	208	257.9	1975.1	-2.8	0.020662298
Tropheryma	2a	3b	7	30	0	2402	257.9	22478.6	-6.28	0.00402138
Ureaplasma	2a	3b	7	30	0	168	257.9	1718.4	-2.59	0.026289638
Actinomyces	2b	3b	6	30	0	806	260.49	6660.4	-4.52	0.017302473
Aeromonas	2b	3b	6	30	0	231	260.5	2373.2	-3.04	0.028784977
Alkalibacterium	2b	3b	6	30	0	772	260.5	6582.7	-4.5	0.010668456

TAX	G1	G2	number of samples G1	number of samples G2	rawCounts G1	rawCounts G2	cpm G1	cpm G2	logFC	FDR
Aquabacterium	2b	3b	6	30	0	1251	260.5	10611.8	-5.19	0.010668456
Atopobium	2b	3b	6	30	0	779	260.5	6449.1	-4.47	0.010668456
Corynebacterium	2b	3b	6	30	0	1384	260.5	12791.1	-5.45	0.010668456
Dolosigranulum	2b	3b	6	30	0	299	260.5	2965.2	-3.36	0.021138721
Enterococcus	2b	3b	6	30	566	0	25889.4	257.4	6.49	6,88E-20
Eubacterium	2b	3b	6	30	0	254	260.5	2268.7	-2.98	0.029173697
Haemophilus	2b	3b	6	30	3358	0	147558.7	257.4	9	1,93E-26
Lactococcus	2b	3b	6	30	0	712	260.5	6243.7	-4.42	0.010668456
Leptotrichia	2b	3b	6	30	0	421	260.5	3630.1	-3.65	0.015349897
Moraxella	2b	3b	6	30	0	4793	260.5	44157.9	-7.24	0.00654039
Neisseria	2b	3b	6	30	5393	0	226976.2	257.4	9.62	3,25E-26
Nocardioides	2b	3b	6	30	0	903	260.5	7714.3	-4.73	0.010668456
Novosphingobium	2b	3b	6	30	0	1052	260.5	9102.4	-4.97	0.010668456
Parvimonas	2b	3b	6	30	0	685	260.5	5973	-4.36	0.012863868
Peptoniphilus	2b	3b	6	30	0	313	260.5	2736.9	-3.25	0.021138721
Planococcus	2b	3b	6	30	0	813	260.5	6681	-4.52	0.010668456
Propionibacterium	2b	3b	6	30	0	377	260.5	3427.1	-3.57	0.034362789
Ralstonia	2b	3b	6	30	0	552	260.5	4676.3	-4.01	0.012863868
Rothia	2b	3b	6	30	0	1076	260.5	9004.1	-4.95	0.010668456
Staphylococcus	2b	3b	6	30	0	4685	260.5	40973.2	-7.13	0.010668456
Treponema	2b	3b	6	30	0	208	260.5	1975.1	-2.78	0.034362789
Tropheryma	2b	3b	6	30	0	2402	260.5	22478.6	-6.27	0.010668456
Ureaplasma	2b	3b	6	30	0	168	260.5	1718.4	-2.58	0.042186912
