# Supplementary material for: Characterisation of Candida within the Mycobiome/Microbiome of the Lower Respiratory Tract of ICU Patients
Source: PLoS One. 2016 May 20;11(5):e0155033. doi: 10.1371/journal.pone.0155033 (PMC4874575; doi:10.1371/journal.pone.0155033)
Supplement: S5 Table — Fungal microbiota richness, evenness and diversity of lower respiratory tract samples from study groups. Richness, Chao1 and ACE (Abundance-based Coverage Estimator) are different indicators of species richness. SDI (Shannon diversity index) and simpson are indicators of diversity. Kruskal-Wallis-Test was used for calculation of p-values (p-values <0.05 = significant). Groups: 1a = healthy adults, 1b = patients with healthy respiratory tract but with antibiotic therapy for extrapulmonary infection, 2a = non-neutropenic intubated and mechanically ventilated ICU patients without antibiotic therapy, 2b = non-neutropenic intubated and mechanically ventilated ICU patients with antibiotic therapy for extrapulmonary infection, and 3b = non-neutropenic intubated and mechanically ventilated ICU patients with antibiotic therapy due to pneumonia. (PDF) [file pone.0155033.s011.pdf]

	Richness (Median, IQR)	Chao1 (Median, IQR)	ACE (Median, IQR)	Evenness (Median, IQR)	SDI (Median, IQR)	Simpson (Median, IQR)
Group 1a	40 (31-49)	74.5 (64.4-76.59)	55.47 (36.65-64.25)	0.5 (0.4-0.6)	1.75 (1.51-2.24)	0.75 (0.56-0.84)
Group 1b	183 (57.5-2739	226.2 (125.9-352.38)	217.25 (85.9-335.93)	0.74 (0.49-0.81)	3.38 (2.2-4.55)	0.95 (0.72-0.98)
Group 2a	28 (21-81)	55.8 (39.25-160.37)	41.71 (25.62-123.79)	0.39 (0.13-0.49)	1.13 (0.46-2.08)	0.55 (0.17-0.76)
Group 2b	17.5 (13.75-20.75)	28.19 (14.92-35.06)	26.3 (16.11-32.11)	0.27 (0.035-0.55)	0.77 (0.098-1.65)	0.41 (0.028-0.77)
Group 3b	19 (9-22.25)	28 (21.34-70)	24.45 (9.44-51.34)	0.31 (0.06-0.51)	0.93 (0.18-1.6)	0.5 (0.06-0.73)

	Richness	Chao 1	ACE	Evenness	SDI	Simpson
1a vs 1b	n.s.	n.s.	n.s.	n.s.	n.s.	n.s.
1a vs 2a	n.s.	n.s.	n.s.	n.s.	n.s.	n.s.

1a vs 2b	0.011	0.02	0.011	n.s.	n.s.	n.s.
1a vs 3b	0.038	n.s.	n.s.	n.s.	0.022	n.s.
1b vs 2a	0.042	n.s.	0.042	0.028	0.028	0.034
1b vs 2b	0.006	0.018	0.006	0.045	0.028	0.028
1b vs 3b	0.001	0.005	0.002	0.005	0.004	0.005
2a vs 2b	0.015	0.016	0.046	n.s.	n.s.	n.s.
2a vs 3b	0.021	n.s.	0.043	n.s.	n.s.	n.s.
2b vs 3b	n.s.	n.s.	n.s.	n.s.	n.s.	n.s.

n.s.=non significant
