# Supplementary material for: Characterisation of Candida within the Mycobiome/Microbiome of the Lower Respiratory Tract of ICU Patients
Source: PLoS One. 2016 May 20;11(5):e0155033. doi: 10.1371/journal.pone.0155033 (PMC4874575; doi:10.1371/journal.pone.0155033)
Supplement: S6 Table — Bacterial microbiota richness, evenness and diversity of lower respiratory tract samples from study groups. Richness, Chao1 and ACE (Abundance-based Coverage Estimator) are different indicators of species richness. SDI (Shannon diversity index) and simpson are indicators of diversity. Kruskal-Wallis-Test was used for calculation of p-values (p-values <0.05 = significant). Groups: 1a = healthy adults, 1b = patients with healthy respiratory tract but with antibiotic therapy for extrapulmonary infection, 2a = non-neutropenic intubated and mechanically ventilated ICU patients without antibiotic therapy, 2b = non-neutropenic intubated and mechanically ventilated ICU patients with antibiotic therapy for extrapulmonary infection, and 3b = non-neutropenic intubated and mechanically ventilated ICU patients with antibiotic therapy due to pneumonia. (PDF) [file pone.0155033.s012.pdf]

| | Richness (Median, IQR) | Chao1 (Median, IQR) | ACE (Median, IQR) | Evenness (Median, IQR) | SDI (Median, IQR) | Simpson (Median, IQR) |
|---|---|---|---|---|---|---|
| Group 1a | 103 (85.25-119.75) | 169.51 (147.36-250.64) | 133.57 (121.19-155.47) | 0.69 (0.55-0.74) | 3.16 (2.5-3.49) | 0.88 (0.79-0.93) |
| Group 1b | 80 (68-111) | 108.5 (92.25-142.25) | 93.99 (90.58-124.74) | 0.58 (0.48-0.63) | 2.46 (2.1-2.75) | 0.78 (0.68-0.78) |
| Group 2a | 107 (92-165) | 194.1 (174-249.48) | 178.21 (131.86-271.13) | 0.52 (0.35-0.63) | 2.39 (1.57-3.24) | 0.75 (0.6-0.92) |
| Group 2b | 79.5 (62.5 -102.75) | 118.01 (92.86 -174.04) | 110.1 (89.14-181.86) | 0.38 (0.3 -0.57) | 1.66 (1.24-2.6) | 0.67 (0.49-0.85) |
| Group 3b | 84 (56.75-123) | 108.06 (67-210.76) | 99.79 (65.1-186.31) | 0.48 (0.3-0.61) | 1.98 (1.5-3) | 0.7 (0.45-0.88) |

| | Richness | Chao 1 | ACE | Evenness | SDI | Simpson |
|---|---|---|---|---|---|---|
| 1a vs 1b | n.s. | 0.015 | 0.037 | n.s. | n.s. | 0.035 |
| 1a vs 2a | n.s. | n.s. | 0.037 | 0.015 | n.s. | n.s. |
| 1a vs 2b | n.s. | n.s. | n.s. | 0.01 | 0.028 | 0.045 |
| 1a vs 3b | n.s. | n.s. | n.s. | 0.013 | 0.005 | 0.013 |
| 1b vs 2a | 0.035 | 0.018 | 0.013 | n.s. | n.s. | n.s. |
| 1b vs 2b | n.s. | n.s. | n.s. | n.s. | n.s. | n.s. |
| 1b vs 3b | n.s. | n.s. | n.s. | n.s. | n.s. | n.s. |
| 2a vs 2b | 0.022 | 0.032 | 0.032 | n.s. | n.s. | n.s. |
| 2a vs 3b | 0.028 | 0.03 | 0.04 | n.s. | n.s. | n.s. |
| 2b vs 3b | n.s. | n.s. | n.s. | n.s. | n.s. | n.s. |

n.s.=non significant
