# Supplementary material for: Characterisation of Candida within the Mycobiome/Microbiome of the Lower Respiratory Tract of ICU Patients
Source: PLoS One. 2016 May 20;11(5):e0155033. doi: 10.1371/journal.pone.0155033 (PMC4874575; doi:10.1371/journal.pone.0155033)
Supplement: S7 Table — Relationships (association/dissociation) between bacteria and fungi in lower respiratory tract samples of healthy adults (group 1a) calculated and depicted as odds ratios. An odds ratio above 2 was considered a positive association, an odds ratio below 0.5 was interpreted as negative association (= dissociation). (PDF) [file pone.0155033.s013.pdf]

Fungi	*Amylostereum*	*Armillaria*	*Aspergillus*	*Bjerkandera*	*Didymella*	*Dioszegia*	*Epicoccum*	*Heterobasidion*	*Malassezia*	*Meira*	*Penicillium*	*Phoma*	*Piptoporus*	*Pluteus*	*Trametes*	*Wallemia*
Bacteria																
Actinomyces	0.56	0.56	0.56	0.56	21	0.56	21	5	0.2	0.56	0.2	21	21	0.56	0.56	0.56
Aquabacterium	0.56	21	0.56	0.56	0.56	0.56	0.56	0.2	5	0.56	5	0.56	0.56	0.56	0.56	0.56
Fusobacterium	0.2	0.2	5	0.2	5	0.2	5	1	1	0.2	1	5	5	0.2	0.2	5
Gemella	0.2	5	5	0.2	0.2	0.2	0.2	0.04	25	0.2	25	0.2	0.2	0.2	0.2	5
Granulicatella	0.56	0.56	21	0.56	0.56	0.56	0.56	0.2	5	0.56	5	0.56	0.56	0.56	0.56	21
Lactococcus	0.56	21	0.56	0.56	0.56	0.56	0.56	0.2	5	0.56	5	0.56	0.56	0.56	0.56	0.56
Oribacterium	0.56	0.56	0.56	0.56	21	0.56	21	5	0.2	0.56	0.2	21	21	0.56	0.56	0.56
Parvimonas	0.56	0.56	0.56	0.56	21	0.56	21	5	0.2	0.56	0.2	21	21	0.56	0.56	0.56
Pasteurella	0.56	0.56	21	0.56	0.56	0.56	0.56	0.2	5	0.56	5	0.56	0.56	0.56	0.56	21
Porphyromonas	0.56	0.56	21	0.56	0.56	0.56	0.56	0.2	5	0.56	5	0.56	0.56	0.56	0.56	21
Prevotella	1.8	1.8	0.05	1.8	1.8	1.8	1.8	5	0.2	1.8	0.2	1.8	1.8	1.8	1.8	0.05
Propionibacterium	0.2	5	5	0.2	0.2	0.2	0.2	0.04	25	0.2	25	0.2	0.2	0.2	0.2	5
Pseudomonas	0.2	5	5	0.2	0.2	0.2	0.2	0.04	25	0.2	25	0.2	0.2	0.2	0.2	5
Staphylococcus	0.56	0.56	21	0.56	0.56	0.56	0.56	0.2	5	0.56	5	0.56	0.56	0.56	0.56	21
Streptobacillus	0.56	0.56	21	0.56	0.56	0.56	0.56	0.2	5	0.56	5	0.56	0.56	0.56	0.56	21
Tropheryma	0.56	0.56	0.56	0.56	21	0.56	21	5	0.2	0.56	0.2	21	21	0.56	0.56	0.56
Veillonella	0.2	0.2	5	0.2	5	0.2	5	1	1	0.2	1	5	5	0.2	0.2	5
