# Supplementary material for: Characterisation of Candida within the Mycobiome/Microbiome of the Lower Respiratory Tract of ICU Patients
Source: PLoS One. 2016 May 20;11(5):e0155033. doi: 10.1371/journal.pone.0155033 (PMC4874575; doi:10.1371/journal.pone.0155033)
Supplement: S8 Table — Relationships (association/dissociation) between bacteria and fungi in lower respiratory tract samples of patients with healthy respiratory tract but with antibiotic therapy for extrapulmonary infection (group 1b) calculated and depicted as odds ratios. An odds ratio above 2 was considered a positive association, an odds ratio below 0.5 was interpreted as negative association (= dissociation). (PDF) [file pone.0155033.s014.pdf]

Fungi	*Amphinema*	*Aspergillus*	*Bjerkandera*	*Cladosporium*	*Cystofilobasidium*	*Dioszegia*	*Entomocorticium*	*Fomitopsis*	*Heterobasidion*	*Hyphodontia*	*Hypholoma*	*Malassezia*	*Mensularia*	*Mrakia*	*Onnia*	*Phlebia*
Bacteria																
Alkalibacterium	0.33	0.33	2	2	0.33	0.33	0.33	7	0.5	0.33	2	2	7	0.33	7	7
Aquabacterium	7	0.33	2	35	0.33	0.33	0.33	7	8.33	0.33	35	0.12	0.33	0.33	0.33	7
Bacteroides	1.29	0.04	3	3	1.29	0.04	1.29	1.29	0.33	0.04	3	0.14	1.29	1.29	1.29	1.29
Bradyrhizobium	1.29	0.04	3	3	1.29	0.04	1.29	1.29	0.33	0.04	3	0.14	1.29	1.29	1.29	1.29
Corynebacterium	0.78	0.78	7	0.33	27	0.78	27	0.78	0.14	0.78	0.33	0.33	0.78	27	0.78	0.78
Janthinobacterium	0.78	0.78	7	7	0.78	0.78	0.78	27	3	0.78	7	0.33	0.78	0.78	0.78	27
Neisseria	0.78	27	0.33	0.33	0.78	27	0.78	0.78	3	27	0.33	7	0.78	0.78	0.78	0.78
Paracoccus	0.78	0.78	0.33	0.33	0.78	0.78	0.78	0.78	0.14	0.78	0.33	7	27	0.78	27	0.78
Prevotella	0.33	7	2	0.12	7	7	7	0.33	0.5	7	0.12	2	0.33	7	0.33	0.33
Propionibacterium	0.78	0.78	0.33	0.33	0.78	0.78	0.78	0.78	0.14	0.78	0.33	7	27	0.78	27	0.78
Pseudomonas	7	0.33	0.12	2	0.33	0.33	0.33	0.33	0.5	0.33	2	2	7	0.33	7	0.33
Ralstonia	0.14	0.14	8.33	0.5	3	0.14	3	3	0.12	0.14	0.5	0.5	3	3	3	3
Rothia	0.78	27	0.33	0.33	0.78	27	0.78	0.78	3	27	0.33	7	0.78	0.78	0.78	0.78
Staphylococcus	0.33	0.33	2	0.12	7	0.33	7	0.33	0.03	0.33	0.12	2	7	7	7	0.33
Streptococcus	0.33	7	2	0.12	7	7	7	0.33	0.5	7	0.12	2	0.33	7	0.33	0.33
