# Supplementary material for: Characterisation of Candida within the Mycobiome/Microbiome of the Lower Respiratory Tract of ICU Patients
Source: PLoS One. 2016 May 20;11(5):e0155033. doi: 10.1371/journal.pone.0155033 (PMC4874575; doi:10.1371/journal.pone.0155033)
Supplement: S9 Table — Relationships (association/dissociation) between bacteria and fungi in lower respiratory tract samples of non-neutropenic intubated and mechanically ventilated ICU patients without antibiotic therapy (group 2a) calculated and depicted as odds ratios. An odds ratio above 2 was considered a positive association, an odds ratio below 0.5 was interpreted as negative association (= dissociation). (PDF) [file pone.0155033.s015.pdf]

Fungi	*Alternaria*	*Armillaria*	*Blumeria*	*Candida*	*Cladosporium*	*Clitocybe*	*Entomocorticium*	*Kazachstania*	*Malassezia*	*Marasmiellus*	*Mycena*	*Penicillium*	*Rigidoporus*	*Wallemia*	unidentified
Bacteria															
Actinomyces	1.22	1.22	39	0.09	1.22	1.22	1.22	1.22	5.4	1.22	39	11	39	1.22	0.19
Bacteroides	0.6	0.6	11	0.02	0.6	0.6	0.6	0.6	15	11	11	4	11	11	0.67
Bradyrhizobium	1.22	1.22	39	0.09	1.22	1.22	1.22	1.22	5.4	1.22	39	11	39	1.22	0.19
Corynebacterium	1.22	39	1.22	1.67	1.22	39	1.22	39	0.33	1.22	1.22	0.6	1.22	1.22	3
Fusobacterium	0.6	11	0.6	3.57	0.6	11	0.6	11	1.5	0.6	0.6	0.28	0.6	0.6	7
Gemella	0.6	0.6	0.6	3.57	11	0.6	11	0.6	0.14	0.6	0.6	4	0.6	0.6	0.67
Granulicatella	0.6	0.6	0.6	0.25	0.6	0.6	0.6	0.6	1.5	11	0.6	0.28	0.6	11	0.67
Haemophilus	11	11	0.6	3.57	0.6	11	0.6	11	0.14	0.6	0.6	0.28	0.6	0.6	0.67
Lactobacillus	1.22	1.22	1.22	1.67	1.22	1.22	1.22	1.22	0.33	1.22	1.22	0.6	1.22	1.22	0.19
Parvimonas	0.6	11	0.6	3.57	0.6	11	0.6	11	1.5	0.6	0.6	0.28	0.6	0.6	7
Prevotella	0.6	11	0.6	3.57	0.6	11	0.6	11	1.5	0.6	0.6	0.28	0.6	0.6	7
Rothia	0.6	0.6	0.6	3.57	11	0.6	11	0.6	0.14	0.6	0.6	4	0.6	0.6	0.67
Solobacterium	1.22	39	1.22	1.67	1.22	39	1.22	39	0.33	1.22	1.22	0.6	1.22	1.22	3
Staphylococcus	1.22	1.22	39	0.09	1.22	1.22	1.22	1.22	5.4	1.22	39	11	39	1.22	0.19
Veillonella	0.6	0.6	0.6	0.25	0.6	0.6	0.6	0.6	1.5	11	0.6	0.28	0.6	11	0.67
