# Supplementary material for: Characterisation of Candida within the Mycobiome/Microbiome of the Lower Respiratory Tract of ICU Patients
Source: PLoS One. 2016 May 20;11(5):e0155033. doi: 10.1371/journal.pone.0155033 (PMC4874575; doi:10.1371/journal.pone.0155033)
Supplement: S10 Table — Relationships (association/dissociation) between bacteria and fungi in lower respiratory tract samples of non-neutropenic intubated and mechanically ventilated ICU patients with antibiotic therapy for extrapulmonary infection (group 2b) calculated and depicted as odds ratios. An odds ratio above 2 was considered a positive association, an odds ratio below 0.5 was interpreted as negative association (= dissociation). (PDF) [file pone.0155033.s016.pdf]

Fungi	*Acremonium*	*Aspergillus*	*Bjerkandera*	*Candida*	*Ceriporiopsis*	*Cladosporium*	*Malassezia*	*Resinicium*	unidentified
Bacteria									
Atopobium	1	1	33	1	1	1	4.2	1	0.24
Bacteroides	1	1	0.03	1	1	1	0.24	1	4.2
Bradyrhizobium	1	1	1	1	1	1	0.24	1	0.24
Enterococcus	1	1	1	1	1	1	0.24	1	0.24
Gemella	1	1	33	1	1	1	4.2	1	0.24
Granulicatella	1	1	33	1	1	1	4.2	1	0.24
Haemophilus	4.2	4.2	0.24	0.24	4.2	4.2	4	4.2	49
Lactobacillus	1	1	33	1	1	1	4.2	1	0.24
Mycoplasma	1	1	1	1	1	1	0.24	1	0.24
Neisseria	1	1	1	1	1	1	4.2	1	4.2
Prevotella	0.47	0.47	9	2.14	0.47	0.47	1	0.47	0.09
Pseudomonas	1	1	1	1	1	1	0.24	1	0.24
Ralstonia	1	1	1	1	1	1	0.24	1	0.24
Streptococcus	1	1	1	1	1	1	4.2	1	4.2
Veillonella	0.47	0.47	9	2.14	0.47	0.47	1	0.47	0.09
