# Supplementary material for: Characterisation of Candida within the Mycobiome/Microbiome of the Lower Respiratory Tract of ICU Patients
Source: PLoS One. 2016 May 20;11(5):e0155033. doi: 10.1371/journal.pone.0155033 (PMC4874575; doi:10.1371/journal.pone.0155033)
Supplement: S2 Table — (PDF) [file pone.0155033.s008.pdf]

Genus level Taxa	087-1A-NTS	095-1A-NTS	097-1A-NTS	098-1B-NTS	099-1B-NTS	100-1B-NTS	101-1B-NTS	102-1B-NTS	103-1B-NTS	104-1B-NTS	105-1A-NTS	106-1A-NTS	107-1A-NTS	108-1A-NTS
Actinomyces	0	0	0	0	0	0	0	3.395311356	0	0	3.591570139	0	0	2.424619198
Aeromonas	0	0	0	0	0	0	0	0	0	0	0	0	0	0
Alishewanella	0	0	2.440570354	0	0	0	0	0	0	0	0	0	0	0
Alkalibacterium	0	0	0	0	4.232804298	0	3.907234907	3.098895311	0	3.210616589	0	0	0	0
Aquabacterium	4.740904331	0	2.694136143	0	3.350969791	0	0	2.506063223	2.725502968	2.868150711	0	0	0	0
Aquitalea	0	0	0	0	0	0	0	0	0	0	0	0	0	0
Atopobium	0	0	0	0	0	0	0	0	0	0	0	0	0	0
Bacteroides	24.10878372	21.17461205	41.4896965	33.51648331	70.89946747	0	51.77716446	56.39989471	33.93899918	55.82191849	12.051054	18.06384659	15.80209827	26.3288784
Bradyrhizobium	2.756339788	5.214643478	7.606972694	16.24803734	4.232804298	0	5.293673038	3.772568226	2.141466618	17.2517128	4.838231087	0	3.328335762	0
Brevundimonas	0	0	0	0	0	0	0	0	0	0	0	0	0	0
Campylobacter	0	0	0	0	0	0	0	0	0	0	0	0	0	0
Capnocytophaga	0	0	0	0	0	0	0	2.047965527	0	0	0	0	0	0
Cloacibacterium	0	0	6.402535439	0	0	0	0	0	0	0	0	0	0	0
Corynebacterium	0	0	0	5.337519169	0	0	0	0	0	0	0	0	0	0
Dermabacter	0	0	0	0	0	0	0	0	0	0	0	0	0	0
Enterococcus	0	0	0	0	0	0	0	0	0	0	0	0	0	0
Eubacterium	0	0	0	0	0	0	0	0	0	0	0	0	0	0
Fusobacterium	0	0	0	0	0	0	0	0	0	0	6.886316299	0	4.077960968	0
Gemella	2.168320656	0	0	0	0	0	0	0	0	0	0	2.180119276	3.448275805	3.0774014
Granulicatella	0	0	0	0	0	0	0	0	0	0	0	0	3.238380671	3.232825518
Haemophilus	0	0	0	0	0	0	0	0	0	0	0	0	0	0
Helicobacter	0	0	0	0	0	0	0	0	0	0	0	0	0	0
Janthinobacterium	0	0	0	0	0	0	0	0	0	2.568493128	0	0	0	0
Lactobacillus	0	0	0	0	0	0	0	0	0	0	0	0	0	0
Lactococcus	2.535832405	0	0	0	0	0	0	0	0	0	0	0	0	0
Leptotrichia	0	0	0	0	0	0	0	0	0	0	0	0	0	0
Leucobacter	0	0	0	0	0	0	0	0	0	0	0	0	0	0
Moraxella	0	0	0	0	0	0	0	0	0	0	0	0	0	0
Mycoplasma	0	0	0	0	0	0	0	0	0	0	0	0	0	0
Neisseria	0	0	0	0	0	7.345191479	0	0	0	0	0	0	0	0
Novosphingobium	0	0	0	0	0	0	0	0	0	0	0	0	0	0
Oribacterium	0	0	0	0	0	0	0	0	0	0	2.523003817	0	0	0
Paracoccus	0	0	0	0	0	0	5.041593552	0	0	0	0	0	0	0
Parvimonas	0	0	0	0	0	0	0	0	0	0	2.582368612	0	0	0
Pasteurella	0	0	0	0	0	0	0	0	0	0	0	0	2.128935575	0
Peptoniphilus	0	0	0	0	0	0	0	0	0	0	0	0	0	0
Planococcus	0	0	0	0	0	0	0	0	0	0	0	0	0	0
Porphyromonas	0	0	0	0	0	0	0	0	0	0	0	0	3.928035975	0
Prevotella	6.100698471	2.027916908	0	5.886970043	0	6.06060648	0	0	0	0	6.826951504	3.270179033	0	13.39757538
Propionibacterium	17.16280937	0	4.881140709	0	0	0	9.301739693	3.260576725	0	0	0	2.621333838	5.457271099	0
Pseudomonas	21.86696243	0	13.59746361	0	0	0	2.873708248	0	45.87929916	0	0	5.320529461	2.968515635	0
Pyramidobacter	0	0	0	0	0	0	0	0	0	0	0	0	0	0
Ralstonia	0	0	0	2.197802067	5.820105553	0	2.72246027	2.506063223	0	2.654109716	0	0	0	0
Rothia	0	0	0	0	0	2.667984247	0	0	0	0	0	0	0	0
Schwartzia	0	0	0	0	0	0	0	0	0	0	0	0	0	0
Silanimonas	0	0	0	0	0	0	0	0	0	0	0	0	0	0
Solobacterium	0	0	0	0	0	0	0	0	0	0	0	0	0	0
Sphingomonas	0	0	0	0	0	0	0	0	0	0	0	0	0	0
Staphylococcus	0	0	0	8.241757393	0	0	4.63826561	0	0	0	0	0	4.737631321	0
Streptobacillus	0	0	0	0	0	0	0	0	0	0	0	0	3.868065834	0
Streptococcus	7.276736736	59.44166565	0	5.572998047	0	73.61660767	0	3.6917274	0	0	40.01187134	7.267064571	24.28785515	23.43798637
Treponema	0	0	0	0	0	0	0	0	0	0	0	0	0	0
Tropheryma	0	0	0	0	0	0	0	0	0	0	2.790145397	48.22216415	0	0
Undibacterium	0	0	2.75752759	0	0	0	0	0	0	0	0	0	0	2.61112833
Ureaplasma	0	0	0	0	0	0	0	0	0	0	0	0	0	0
Variovorax	0	0	0	0	0	0	0	0	0	0	0	0	0	0
Veillonella	0	0	0	0	0	0	0	0	0	0	2.285544634	0	5.96701622	0
Other	11.28261757	12.14116478	18.12995148	22.99842834	11.4638443	10.309618	14.44416523	19.32093811	15.31473064	15625	15.61294174	13.05476189	16.76161957	25.48958588

Genus level Taxa	109-1A-NTS	201-2A-NTS	202-2A-NTS	203-2A-NTS	252-2B-NTS	255-2B-NTS	256-2B-NTS	257-2B-NTS	301-3B-VAP	302-3B-VAP	303-3B-VAP	304-3B-ASP	305-3B-VAP	306-3B-VAP
Actinomyces	0	0	4.347826004	0	0	0	0	0	0	0	0	0	0	0
Aeromonas	0	0	0	0	0	0	0	0	0	0	0	0	0	0
Alishewanella	0	0	0	0	0	0	0	0	0	0	0	0	0	0
Alkalibacterium	2.951626062	0	0	0	0	0	0	0	0	0	0	0	0	0
Aquabacterium	2.541677952	0	0	0	0	0	0	0	0	0	0	0	0	0
Aquitalea	0	0	0	0	0	0	0	0	0	0	0	9.090909004	0	0
Atopobium	0	0	0	0	0	0	2.566009521	0	0	0	6.803637505	0	0	5.178090096
Bacteroides	57.55670929	0	6.434145451	0	2.363593578	34.10680389	0	8.63367939	19.00635338	0	0	18.18181801	0	0
Bradyrhizobium	10.90461826	0	2.516324043	0	0	6.802289009	0	0	6.383593082	0	0	18.18181801	0	0
Brevundimonas	0	0	0	0	0	0	0	0	0	0	0	0	0	0
Campylobacter	0	0	0	0	0	0	0	0	0	0	0	0	0	0
Capnocytophaga	0	0	0	0	0	0	0	0	0	0	0	0	0	0
Cloacibacterium	0	0	0	0	0	0	0	0	0	0	0	0	0	0
Corynebacterium	0	0	0	0	0	0	0	0	0	0	0	18.18181801	0	0
Dermabacter	0	0	0	0	0	0	0	0	0	0	0	0	0	0
Enterococcus	0	0	0	0	0	0	0	0	0	0	0	0	0	0
Eubacterium	0	0	0	0	0	0	0	0	0	0	2.728191376	0	0	0
Fusobacterium	0	0	0	0	0	0	0	0	0	0	0	9.090909004	0	0
Gemella	0	17.11131477	0	43.8413353	0	0	5.355150223	0	0	0	0	18.18181801	34.4108429	0
Granulicatella	0	3.357865572	0	0	0	0	4.202305794	0	0	0	7.005725861	0	0	12.33163738
Haemophilus	0	0	0	0	15.00470352	0	0	44.65485764	0	0	0	0	0	0
Helicobacter	0	0	0	0	0	0	0	0	0	0	0	0	0	0
Janthinobacterium	0	0	0	0	0	0	0	0	0	0	0	0	0	0
Lactobacillus	0	2.667893171	0	0	0	0	3.272592068	0	0	0	0	0	2.685088634	0
Lactococcus	0	0	0	0	0	0	0	0	0	0	0	0	0	0
Leptotrichia	0	0	0	0	0	0	0	0	0	0	0	0	0	0
Leucobacter	0	0	0	0	0	0	0	0	0	0	0	0	0	0
Moraxella	0	0	0	0	0	0	0	0	0	0	0	0	0	0
Mycoplasma	0	0	0	0	0	17.99110031	0	0	34.54650497	0	42.60693741	0	0	0
Neisseria	0	0	0	0	63.41721725	0	0	0	0	0	0	0	0	0
Novosphingobium	0	0	0	0	0	0	0	0	0	0	0	0	0	0
Oribacterium	0	0	0	0	0	0	0	0	0	0	0	0	0	0
Paracoccus	0	0	0	0	0	0	0	0	0	0	0	0	0	0
Parvimonas	0	0	0	0	0	0	0	0	0	0	4.378578663	0	0	0
Pasteurella	0	0	0	0	0	0	0	0	0	0	0	0	0	0
Peptoniphilus	0	0	0	0	0	0	0	0	0	0	3.772313833	0	0	0
Planococcus	0	0	0	0	0	0	0	0	0	0	0	0	0	0
Porphyromonas	0	0	0	0	0	0	0	0	0	0	0	0	0	2.095180988
Prevotella	0	0	0	0	0	2.256834269	21.12309265	0	5.661467075	0	22.63388252	0	0	18.79676628
Propionibacterium	6.012571812	0	0	0	0	0	0	0	2.28191781	0	0	0	0	0
Pseudomonas	3.607542992	0	0	0	0	25.58804893	0	0	0	99.77894592	0	0	0	0
Pyramidobacter	0	0	0	0	0	0	0	0	0	0	0	0	0	0
Ralstonia	2.241049528	0	0	0	0	2.129688501	0	0	0	0	0	0	0	0
Rothia	0	17.61729431	0	5.427975178	0	0	0	0	0	0	0	0	2.163712263	24.60341263
Schwartzia	0	0	0	0	0	0	0	0	2.599653244	0	0	0	0	0
Silanimonas	0	0	0	0	0	0	0	0	0	0	0	0	0	0
Solobacterium	0	0	0	0	0	0	0	0	0	0	0	0	0	0
Sphingomonas	0	0	0	0	0	0	0	0	0	0	0	0	0	0
Staphylococcus	2.978955984	0	20.06688881	0	0	0	0	0	0	0	0	0	7.586027145	0
Streptobacillus	0	0	0	0	0	0	0	0	0	0	0	0	0	0
Streptococcus	2.159059763	54.64581299	61.37919617	45.09394455	18.28551292	0	54.77872849	41.27468872	2.195262909	0	0	9.090909004	51.01668549	27.23735428
Treponema	0	0	0	0	0	0	0	0	6.008087635	0	0	0	0	0
Tropheryma	0	0	0	0	0	0	0	0	0	0	0	0	0	0
Undibacterium	0	0	0	0	0	0	0	0	0	0	0	0	0	0
Ureaplasma	0	0	0	0	0	0	0	0	0	0	0	0	0	0
Variovorax	0	0	0	0	0	0	0	0	0	0	0	0	0	0
Veillonella	0	3.265869379	0	0	0	0	4.760133743	0	0	0	3.031323671	0	0	0
Other	9.046187401	1.333946586	5.255613804	5.636743069	0.928974628	11.12523937	3.941985846	5.436774731	21.31715775	0.221050844	7.039407253	0	2.137643337	9.757557869

Genus level Taxa	309-3B-VAP	310-3B-NAP	312-3B-VAP	313-3B-NAP	314-3B-CAP	318-3B-ASP	319-3B-VAP	320-3B-ASP	321-3B-NAP	322-3B-NAP	323-3B-VAP	324-3B-ASP	325-3B-VAP	326-3B-VAP
Actinomyces	0	0	0	0	0	0	0	0	2.799552202	0	0	7.405935287	0	0
Aeromonas	0	0	0	21875	0	0	0	0	0	0	0	0	0	0
Alishewanella	0	0	0	0	0	0	0	0	0	0	0	0	0	0
Alkalibacterium	0	0	0	0	0	0	3.370786667	0	0	4.37291193	3.977000475	0	0	3.2410779
Aquabacterium	0	0	0	0	0	0	10.30608273	0	0	0	5.606132984	0	0	3.605243921
Aquitalea	0	0	0	0	0	0	0	0	0	0	0	0	0	0
Atopobium	0	0	0	0	0	0	0	0	0	0	0	0	0	0
Bacteroides	7.964059353	0	0	0	11.1153698	0	63.5025177	0	0	30.0941391	32.75035858	23.58240509	0	23.34304428
Bradyrhizobium	0	0	0	0	0	0	3.409531116	0	0	5.162465572	4.935313702	2.503974438	0	3.568827391
Brevundimonas	0	0	0	0	0	0	0	0	0	2.45976305	0	0	0	0
Campylobacter	0	0	0	0	0	0	0	0	0	0	0	0	0	2.294246197
Capnocytophaga	0	0	0	0	0	0	0	0	0	0	0	0	0	0
Cloacibacterium	0	0	0	0	0	0	0	0	0	0	0	0	0	0
Corynebacterium	0	0	0	0	0	0	0	0	0	0	0	0	0	0
Dermabacter	0	0	0	0	0	0	0	0	0	0	0	0	0	3.932993412
Enterococcus	0	0	0	0	0	0	0	0	0	0	0	0	0	0
Eubacterium	0	0	0	0	0	0	0	0	0	0	0	0	0	0
Fusobacterium	0	3.066841364	0	0	0	0	0	0	0	0	0	0	0	0
Gemella	0	34.23328781	0	0	0	0	0	0	10.86226177	0	0	3.378378391	0	0
Granulicatella	0	2.175622463	0	0	0	0	0	0	5.347144604	0	0	0	0	0
Haemophilus	0	0	0	0	0	0	0	0	0	0	0	0	0	0
Helicobacter	0	0	0	0	0	0	0	0	0	0	2.731193066	0	0	0
Janthinobacterium	0	0	0	0	0	0	0	0	0	0	0	0	0	0
Lactobacillus	4.16581583	0	0	0	0	0	0	0	0	0	0	0	0	0
Lactococcus	14.53951359	0	0	0	0	0	0	0	0	0	0	0	0	0
Leptotrichia	0	0	0	0	0	0	0	0	0	0	0	3.855325699	0	4.734158516
Leucobacter	0	0	0	0	0	0	2.28593564	0	0	0	0	0	0	0
Moraxella	0	0	0	0	0	0	0	88.89095306	0	0	0	0	0	0
Mycoplasma	0	0	0	0	0	0	0	0	0	0	0	0	76.60055542	13.80189323
Neisseria	0	0	0	0	0	0	0	0	0	0	0	0	0	0
Novosphingobium	0	0	0	0	0	0	0	0	0	31.94655228	0	0	0	0
Oribacterium	0	0	0	0	0	0	0	0	0	0	0	0	0	0
Paracoccus	0	0	0	0	0	0	0	0	0	0	0	0	0	0
Parvimonas	0	0	0	0	0	0	0	0	0	0	0	0	9.364800453	0
Pasteurella	0	0	0	0	0	0	0	0	0	0	0	0	0	0
Peptoniphilus	0	0	0	0	0	0	0	0	0	0	0	0	0	0
Planococcus	0	0	0	0	0	0	0	0	0	0	0	10.77106476	0	0
Porphyromonas	0	0	0	0	0	0	0	0	0	0	0	0	0	0
Prevotella	0	0	0	0	0	3.216494799	0	0	2.239641666	0	2.012458086	16.58717537	4.042179108	3.787326813
Propionibacterium	0	0	0	0	0	0	0	0	0	3.188581705	0	0	0	2.767662048
Pseudomonas	0	0	0	0	0	0	0	0	0	0	0	0	0	0
Pyramidobacter	0	0	0	0	0	0	0	0	0	0	2.084331512	0	0	0
Ralstonia	0	0	0	0	0	0	2.440914392	0	0	3.552991152	3.665548563	0	0	0
Rothia	0	0	0	0	0	0	0	0	7.138857841	0	0	0	0	0
Schwartzia	0	0	0	0	0	0	0	0	0	0	0	0	0	0
Silanimonas	0	0	0	0	0	0	0	0	0	0	0	0	0	2.002913237
Solobacterium	0	0	0	0	0	0	0	0	0	0	0	0	0	0
Sphingomonas	0	0	0	0	0	0	3.680743933	0	0	0	0	0	0	0
Staphylococcus	0	0	0	72.72727203	0	64.0618515	0	0	0	0	12.6257782	0	0	0
Streptobacillus	0	0	0	0	0	0	0	0	0	0	0	0	0	0
Streptococcus	65.95874786	50.17037964	99.47110748	0	80.5480957	26.98969078	0	5.637982368	66.88130188	2.550865412	16.05174828	9.989400864	2.862164259	14.31172562
Treponema	0	0	0	0	0	0	0	0	0	0	3.402012348	0	0	0
Tropheryma	0	0	0	0	0	0	0	0	0	0	0	0	0	0
Undibacterium	0	0	0	0	0	0	0	0	0	0	0	0	0	0
Ureaplasma	0	4.403669834	0	0	0	0	0	0	0	0	0	0	0	0
Variovorax	0	0	0	0	0	0	0	0	0	2.095353603	0	0	0	0
Veillonella	0	0	0	0	0	0	0	0	0	0	0	4.557498455	0	0
Other	7.371860027	5.950196743	0.528893232	5.397727013	8.336526871	5.731958389	11.00348759	5.471068382	4.731243134	14.57637405	10.15812111	17.36883926	7.13030386	18.60888481

Genus level Taxa	327-3B-ASP	328-3B-NAP	401-2A-NTS	402-2A-NTS	403-2A-NTS	405-2B-NTS	406-2A-NTS	608-3B-ASP	609-3B-ASP	610-2B-NTS	612-3B-VAP
Actinomyces	0	0	0	0	0	0	0	0	0	0	0
Aeromonas	0	0	0	0	0	0	0	0	0	0	0
Alishewanella	0	0	0	0	0	0	0	0	0	0	0
Alkalibacterium	0	3.36391449	0	0	0	0	0	0	0	0	2.700675249
Aquabacterium	0	8.395885468	0	0	0	0	0	0	0	0	0
Aquitalea	0	0	0	0	0	0	0	0	0	0	0
Atopobium	0	0	0	0	0	0	0	0	0	0	0
Bacteroides	0	69.44676208	0	13.01829338	0	2.489052773	0	0	0	15.10379314	23.7059269
Bradyrhizobium	0	0	0	0	0	0	0	0	0	0	23.0307579
Brevundimonas	0	0	0	0	0	0	0	0	0	0	0
Campylobacter	0	0	0	0	0	0	0	0	0	0	0
Capnocytophaga	0	0	0	0	0	0	0	0	0	0	0
Cloacibacterium	0	0	0	0	0	0	0	0	0	0	0
Corynebacterium	0	0	0	0	6.429096222	0	0	0	0	0	0
Dermabacter	0	0	0	0	0	0	0	0	0	0	0
Enterococcus	0	0	0	0	0	13.04447937	0	0	0	0	0
Eubacterium	0	0	0	0	0	0	0	0	0	0	0
Fusobacterium	0	0	0	0	18.18515778	0	7.082273006	0	0	0	0
Gemella	0	0	0	0	0	0	0	6.352343559	0	0	0
Granulicatella	0	0	0	2.713414669	0	0	0	0	0	0	0
Haemophilus	0	0	19.92779922	0	20.72005844	0	0	0	0	6.800286293	0
Helicobacter	0	0	0	0	0	0	0	0	0	0	0
Janthinobacterium	0	2.752293587	0	0	0	0	0	0	0	0	0
Lactobacillus	4.50061655	0	0	0	0	0	0	0	0	0	0
Lactococcus	0	0	0	0	0	0	0	0	0	0	0
Leptotrichia	0	0	0	0	0	0	0	0	0	0	0
Leucobacter	0	0	0	0	0	0	0	0	0	0	0
Moraxella	0	0	0	0	0	0	0	0	0	0	0
Mycoplasma	0	0	0	0	0	0	0	0	0	0	0
Neisseria	0	0	0	3.871951342	0	0	0	0	0	0	0
Novosphingobium	0	0	0	0	0	0	0	0	0	0	0
Oribacterium	0	0	0	0	0	0	0	0	0	0	0
Paracoccus	0	0	0	0	0	0	0	0	0	0	0
Parvimonas	0	0	0	0	12.564291	0	11.53519917	0	0	0	0
Pasteurella	0	0	0	0	0	0	0	0	0	0	0
Peptoniphilus	0	0	0	0	0	0	0	0	0	0	0
Planococcus	0	0	0	0	0	0	0	0	0	0	0
Porphyromonas	0	0	0	0	0	0	0	0	0	0	0
Prevotella	0	0	0	0	20.38941956	0	14.46140766	41.21965027	0	0	0
Propionibacterium	0	2.418682337	0	0	0	0	0	0	0	0	28.28207016
Pseudomonas	0	0	0	0	0	0	0	0	0	0	0
Pyramidobacter	0	0	0	0	0	0	0	0	0	0	0
Ralstonia	0	2.279677629	0	0	0	0	0	0	0	0	5.326331615
Rothia	0	0	0	0	0	0	0	0	0	0	0
Schwartzia	0	0	0	0	0	0	0	0	0	0	0
Silanimonas	0	0	0	0	0	0	0	0	0	0	0
Solobacterium	0	0	0	0	9.515062332	0	0	0	0	0	0
Sphingomonas	0	0	0	0	0	0	0	0	0	0	0
Staphylococcus	0	0	0	0	0	0	0	0	0	0	3.525881529
Streptobacillus	0	0	0	0	0	0	0	0	0	0	0
Streptococcus	87.44348145	0	77.32852173	66.37195587	2.0940485	78.52039337	64.92790222	50	14.73000717	77.02218628	0
Treponema	0	0	0	0	0	0	0	0	0	0	0
Tropheryma	0	0	0	0	0	0	0	0	82.09159088	0	0
Undibacterium	0	0	0	0	0	0	0	0	0	0	0
Ureaplasma	0	0	0	0	0	0	0	0	0	0	0
Variovorax	0	0	0	0	0	0	0	0	0	0	0
Veillonella	0	0	0	4.725609779	0	3.15740943	0	0	0	0	0
Other	8.055897713	11.34278584	2.743682384	9.298780441	10.10286522	2.788660765	1.993214607	2.428006887	3.178400517	1.073729396	13.42835712
